# Metabolic and transcriptional regulatory mechanisms underlying the anoxic adaptation of rice coleoptile

**DOI:** 10.1093/aobpla/plu026

**Published:** 2014-06-03

**Authors:** Meiyappan Lakshmanan, Bijayalaxmi Mohanty, Sun-Hyung Lim, Sun-Hwa Ha, Dong-Yup Lee

**Affiliations:** 1 Department of Chemical and Biomolecular Engineering, National University of Singapore, 4 Engineering Drive 4, Singapore 117585, Singapore; 2 Divison of Metabolic Engineering, National Academy of Agricultural Science, Suwon 441707, Republic of Korea; 3 Graduate School of Biotechnology and Crop Biotech Institute, Kyung Hee University, Yongin 446701, Republic of Korea; 4 Bioprocessing Technology Institute, Agency for Science, Technology and Research (A*STAR), 20 Biopolis Way, #06-01, Centros, Singapore 138668, Singapore

**Keywords:** Anoxia, *cis*-elements, flux sampling, rice, systems biology, transcription factors, transcriptional regulation.

## Abstract

The ability of rice to germinate under anoxia by extending the coleoptile is a highly unusual characteristic and a key feature underpinning the ability of rice seeds to establish in such a stressful environment. The process has been a focal point for research for many years. However, the molecular mechanisms underlying the anoxic growth of the coleoptile still remain largely unknown. To unravel the key regulatory mechanisms of rice germination under anoxic stress, we combined *in silico* modelling with gene expression data analysis. Our initial modelling analysis via random flux sampling revealed numerous changes in rice primary metabolism in the absence of oxygen. In particular, several reactions associated with sucrose metabolism and fermentation showed a significant increase in flux levels, whereas reaction fluxes across oxidative phosphorylation, the tricarboxylic acid cycle and the pentose phosphate pathway were down-regulated. The subsequent comparative analysis of the differences in calculated fluxes with previously published gene expression data under air and anoxia identified at least 37 reactions from rice central metabolism that are transcriptionally regulated. Additionally, *cis*-regulatory content analyses of these transcriptionally controlled enzymes indicate a regulatory role for transcription factors such as MYB, bZIP, ERF and ZnF in transcriptional control of genes that are up-regulated during rice germination and coleoptile elongation under anoxia.

## Abstract (precis)

Rice is a unique crop plant since it can survive under anoxia condition by flooding and also germinate and grow up to coleoptile through its distinctive adaptations. Despite several decades of research on this topic, the current knowledge on the molecular machinery of rice under anoxia is very limited. Therefore, we unraveled the possible regulatory mechanisms by resorting to systems biology approach which combines the metabolic modeling and transcriptome analysis. Such integrative analysis highlight the critical role of MYB, bZIP, ERF and ZnF transcription factors in up-regulating the fermentation and sucrose metabolism genes to generate sufficient energy for cellular growth.

## Introduction

Rice is one of the few plant species that can survive submerged conditions. In particular, it can successfully germinate by extending a coleoptile underwater in the complete absence of oxygen (Magneschi and Perata 2009). Most of the early biochemical studies reported that even under anoxia, rice is able to metabolize reserve carbohydrates via glycolysis and fermentation to generate sufficient cellular energy, i.e. ATP needed for plant growth (Mocquot *et al.* 1981; Alpi and Beevers 1983; Mohanty *et al.* 1993; Guglielminetti *et al.* 1995). Microarray data show that this is associated with a strong up-regulation of sucrose and starch metabolism, glycolysis and fermentation genes (Lasanthi-Kudahettige *et al.* 2007). This work also showed many other significantly up- and down-regulated genes associated with various metabolic and signalling pathways. These findings highlight that rice globally reconfigures its molecular machinery under anoxic stress by selectively synthesizing the necessary transcripts coding for enzymes that produce and conserve energy.

Despite past efforts to characterize the biochemical adaptations of rice under anoxia, current knowledge on how oxygen deficiency is sensed and on the regulatory cascade that fine-tunes the transcriptional and translational changes is still very limited. To date, only the induction of GA-response-free RAmy3D under anoxic conditions (Loreti *et al.* 2003*a*, *b*) is the notable trait unravelled at the molecular level. Even with the availability of abundant high-throughput data, such limitations still exist mainly due to the lack of appropriate systematic frameworks to analyse and derive a valid hypothesis from them. In this regard, constraint-based *in silico* metabolic modelling and analysis can be helpful. Not only do they allow predictions concerning the physiological behaviour and metabolic states of an organism exposed to various environmental or genetic changes but they also serve as a scaffold to contextualize multiple ‘-omics’ data, thereby enabling biologically meaningful correlations to be identified (Lewis *et al.* 2012). As a result, several frameworks are now available to integrate metabolic models and high-throughput data such as transcriptomics (Becker and Palsson 2008; Colijn *et al.* 2009; Bordel *et al.* 2010), metabolomics (Mo *et al.* 2009; Selvarasu *et al.* 2012) and proteomics (Shlomi *et al.* 2008) within the context of systems biology. One such approach compares the flux levels simulated by the metabolic model with corresponding gene expression data resulting from environmental or genetic changes. This has, for example, highlighted key transcriptional mechanisms in *Saccharomyces cerevisiae* (Famili *et al.* 2003; Bordel *et al.* 2010; Osterlund *et al.* 2013). Therefore, following a similar method, here, we identified the transcriptionally regulated reactions in rice during its anoxic germination. Our approach used initial random flux sampling using the recently published rice central metabolic network (Lakshmanan *et al.* 2013), and subsequently compared the differences in flux levels with previously published gene expression data (Lasanthi-Kudahettige *et al.* 2007) between air and anoxia. Furthermore, to gain a deeper insight into the key regulatory mechanisms during anoxic adaptation, we also found the possible transcription factors (TFs) of transcriptionally regulated enzymes by analysing the distribution of putative *cis*-elements in their promoter regions.

## Methods

### Rice central metabolic network

We used the recently published rice central metabolic model (Lakshmanan *et al.* 2013) to characterize the metabolic states of germinating rice seeds under air and anoxia. The gene–protein reaction (GPR) mappings were updated for all reactions since the original model did not include the unique genes associated with enzymes having multiple isozymes across different cellular compartments. The updated model is provided in **Supporting Information**.

### Microarray data

The raw transcriptome data generated by Lasanthi-Kudahettige *et al.* (2007) were first downloaded from the Gene Expression Omnibus (accession no. GSE6908) and normalized using the quantile method (Bolstad *et al.* 2003). Differentially expressed genes were then identified by a linear model (Wettenhall and Smyth 2004). The resulting *P* values were also corrected for multiple testing using Benjamini–Hochberg correction.

### Random flux sampling

The artificial centring hit-and-run (ACHR) Monte Carlo sampling (Thiele *et al.* 2005; Mo *et al.* 2009) was utilized to sample uniformly the metabolic flux solution space under aerobic and anaerobic conditions with appropriate flux constraints. Under both conditions, we constrained sucrose uptake rate, oxygen uptake rate and cell growth rate with experimentally measured values (Lakshmanan *et al.* 2013). To make a fair comparison between both conditions, oxygen uptake and growth rates were normalized with respect to sucrose uptake rate. The solution space was sampled with 100 000 randomly distributed points for 10 000 iterations in each simulation. In this study, the COBRA toolbox (Schellenberger *et al.* 2011) was utilized to implement the random flux sampling.

The differences in flux samples between aerobic and anaerobic conditions were quantified using a *Z*-score approach as described previously (Mo *et al.* 2009). In this approach, two random flux vectors, *v_j_*, one from each sample, i.e. aerobic and anaerobic, were chosen and the difference is calculated as follows:

$$v_{\;j,\mathrm{diff}}=|v_{\;j,\mathrm{aerobic}}-v_{\;j,\mathrm{anaerobic}}|$$

This approach was repeated 10 000 times to create new flux differences, *v_j_*_,diff_, sampled with 10 000 points. From this flux difference sample, the sample mean, *μ_j_*, and standard deviation,* σ_j_*, were computed to calculate the *Z*-score as follows:

$$Z_{j}=\frac{\mu_{j}}{\sigma_{j}/\sqrt{n}}$$

Finally, the absolute *Z*-scores were translated to *P* values using the normal cumulative distribution function and the reactions with *P* values <0.05 were deemed as statistically different between aerobic and anaerobic conditions.

### Identification of transcriptionally regulated enzymes

Using the *P* values of transcriptome data and flux sampling, we identified the reactions that are transcriptionally regulated (Bordel *et al.* 2010). Briefly, if the flux and gene expression (for both up- and down-regulated) significantly changes in the same direction, the corresponding enzyme is classified as ‘transcriptionally regulated’. On the other hand, if the values significantly change in the opposite direction, the enzyme is classified as ‘metabolically regulated’. In the case of reactions with multiple isozymes, the gene expression was considered to be up- or down-regulated based on the expression values of the majority of the transcripts. For example, if a gene has more up-regulated transcripts than down-regulated ones, then it is considered as up-regulated.

### Motif detection and identification of putative TFs

Promoter sequences [−1000, +200 nt relative to the transcription start site (TSS)] for transcriptionally up- and down-regulated genes related to anaerobic central metabolism were extracted from our in-house rice promoter sequence database. Known and novel promoter motifs were detected using the Dragon Motif Builder program with EM2 option (Huang *et al.* 2005). Thirty motifs were detected each time having a length of 8–10 nucleotides per detection at a threshold value of 0.875. Motif occurrences in over 50 % of the sequences at a threshold e value of ≤10^−3^ were considered as statistically overrepresented. Motif classes were identified by their matches in different plant Transcription Factor Binding databases such as TRANSFAC (Matys *et al.* 2003; http://www.gene-regulation.com), PLACE (Higo *et al.* 1999; http://www.dna.affrc.go.jp/htdocs/PLACE), AGRIS (Yilmaz *et al.* 2011; http://arabidopsis.med.ohio-state.edu/) and Osiris (Morris *et al.* 2008; http://www.bioinformatics2.wsu.edu/cgi-bin/Osiris/cgi/home.pl). A similar method was used for the extraction and detection of motifs in the negative sets.

## Results

### Random sampling reveals significant differences in rice central metabolism between aerobic and anaerobic conditions

We sampled the plausible metabolic states of rice coleoptile in air and under anoxia using ACHR Monte Carlo sampling to estimate the range of possible steady-state flux values through each of the reactions in the rice model (see Methods). The resulting probability distributions of individual reaction fluxes revealed significant differences in central metabolic pathways such as glycolysis, the tricarboxylic acid (TCA) cycle, the pentose phosphate pathway and oxidative phosphorylation between aerobic and anaerobic conditions **[see Supporting Information]**.

Under anoxia, the TCA cycle, the pentose phosphate pathway and oxidative phosphorylation had only a small range of fluxes, with some even having zero flux due to the imposed capacity constraints, i.e. the absence of oxygen exchange **[see Supporting Information]**. As a result, the fluxes across various amino acid and lipid synthetic pathways were also severely restricted under anoxic conditions (data not shown). On the other hand, random sampling allowed us to observe the possibility of high fluxes through cytosolic glycolysis and fermentation, mainly to produce all the ATP required for anaerobic cell growth. Other energy-producing pathways such as oxidative phosphorylation and the TCA cycle are grossly impaired under such conditions (Mohanty *et al.* 1993; Lakshmanan *et al.* 2013).

### Transcriptionally regulated reactions during anaerobic adaptation

We compared the differences in flux samples and gene expression data between air and anoxia, thereby identifying the reactions that are likely to be transcriptionally regulated (see Methods). Overall, among the 63 reactions in rice central metabolism, 37 and 5 exhibit transcriptional and metabolic regulation, respectively. The remaining 38 reactions could not be classified in any of these categories as they exhibited an insignificant change in either flux or gene expression. The complete lists of transcriptionally and metabolically regulated reactions are provided in **Supporting Information**.

**Figure 1.**
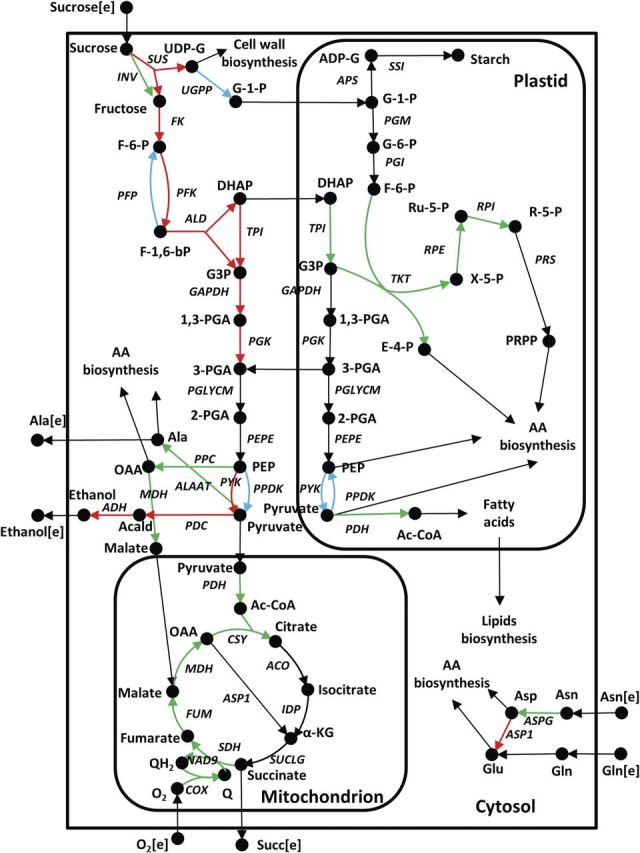
Central metabolic reactions of rice showing transcriptional and metabolic regulation under anoxia. Green, red and blue colours indicate the transcriptionally down-, up- and metabolically regulated enzymes, respectively. Reactions with black arrows represent enzymes whose regulation mechanism has not been investigated or identified in this study. Metabolite abbreviations are as follows: α-KG, α-ketoglutarate; 1,3-PGA, 1,3-diphosphoglycerate; 2-PGA, 2-phosphoglycerate; 3-PGA, 3-phosphoglycerate; AA, amino acids; Acald, acetaldehyde; Ac-CoA, acetyl-coenzyme A; ADP-G, ADP-glucose; Ala, alanine; Asn, asparagine; Asp, aspartate; DHAP, dihydroxyacetone phosphate; E-4-P, erythrose-4-phosphate; F-6-P, fructose-6-phosphate; F-1,6-bP, fructose-1,6-bisphosphate; G-1-P, glucose-1-phosphate; G3P, glyceraldehyde-3-phosphate; G-6-P, glucose-6-phosphate; Gln, glutamine; Glu, glutamate; OAA, oxaloacetate; PEP, phosphoenolpyruvate; PRPP, phosphoribosyl pyrophosphate; Q, ubiquinone; QH2, ubiquinol; R-5-P, ribose-5-phosphate; Ru-5-P, ribulose-5-phosphate; UDP-G, UDP-glucose; X-5-P, d-xylulose-5-phosphate. Enzyme abbreviations are as follows: ACO, aconitase; ADH, alcohol dehydrogenase; ALAAT, alanine aminotransferase; ALD, aldolase; APS, glucose-1-phosphate adenylyltransferase; ASP1, aspartate aminotransferase; ASPG, asparaginase; COX, cytochrome *c* oxidase; CSY, citrate synthase; FK, fructokinase; FUM, fumarase; GAPDH, glyceraldehyde phosphate dehydrogenase; IDP, isocitrate dehydrogenase (NADP-dependent); INV, invertase; MDH, malate dehydrogenase; NAD9, NADH dehydrogenase; PDC, pyruvate decarboxylase; PDH, pyruvate dehydrogenase; PEPE, phosphoenolpyruvate enolase; PFK, 6-phosphofructokinase; PFP, PPi-dependent phosphofructokinase; PGI, phosphoglucoisomerase; PGK, phosphoglycerate kinase; PGLYCM, phosphoglucomutase; PGM, phosphoglucomutase; PPC, phosphoenolpyruvate carboxylase; PPDK, pyruvate orthophosphate dikinase; PRS, ribose-phosphate diphosphokinase; PYK, pyruvate kinase; RPE, ribose-5-phosphate epimerase; TKT, transketolase; TPI, triose phosphate isomerase; SDH, succinate dehydrogenase; SSI, starch synthase; SUCLG, succinyl-CoA ligase; SUS, sucrose synthase; UGPP, UDP-glucose pyrophosphorylase.

Most of the reactions in the TCA cycle, oxidative phosphorylation and the pentose phosphate pathway show down-regulation in both flux and gene expression under anaerobic conditions while several reactions of sucrose metabolism including sucrose synthase (SUS), fructokinase and nucleoside diphosphate kinase are significantly up-regulated at the transcriptional level (Fig. 1). Only invertase in sucrose metabolism shows down-regulation in both flux and gene expression, confirming that rice preferably utilizes SUS to metabolize sucrose under anaerobic conditions. The use of SUS conserves ATP usage (Guglielminetti *et al.* 1995; Lakshmanan *et al.* 2013).

All the reactions in the fermentation pathway including pyruvate decarboxylase (PDC), alcohol dehydrogenase (ADH) and aldehyde dehydrogenase (ALDH) were up-regulated (Fig. 1). In rice, both alcohol and aldehyde dehydrogenases have multiple isozymes and a few of them such as *ADH1*, *ADH2* and *ALDH2a* showed a drastic increase in gene expression (Lasanthi-Kudahettige *et al.* 2007). Interestingly, the up-regulation of ALDH at both flux and transcriptional level is in very good agreement with earlier experimental reports on both tolerant and intolerant lines of rice under hypoxic conditions during submergence (Nakazono *et al.* 2000; Ismail *et al.* 2012; Miro *et al.* 2013), indicating that ALDH is likely to play a key role in detoxifying the excess acetaldehyde from PDC that cannot be metabolized via ADH. In this regard, Lasanthi-Kudahettige *et al.* (2007) hypothesized that the acetate resulting from ALDH can enter the TCA cycle to further fuel amino acid synthesis pathways. However, our flux analysis revealed that the acetate from ALDH primarily fuels lipid biosynthesis in plastids, as fatty acid synthesis needs sufficient carbon flux to synthesize the required saturated fatty acids under anoxia.

Unlike other central metabolic pathways, the glycolytic reactions did not show an overall up- or down-regulation in gene transcription and fluxes (Fig. 1). Out of a total of 25 reactions, only 11 showed transcriptional regulation. Among the remaining 14, 5 were oppositely correlated between flux and gene expression, indicating that these enzymes are most likely to be metabolically regulated. Plastidic pyruvate kinase, UDP-glucose pyrophosphorylase, PPi-dependent phosphofructokinase (PFP), and cytosolic and plastidic pyruvate phosphate dikinase (PPDK) are the five enzymes with metabolic regulation. Such observations are in good agreement with the earlier hypothesis by Plaxton (1996), who suggested that PK, PPDK, PFK and PFP are involved in the fine control of plant glycolysis and are most likely to be regulated based on the variation in substrate(s) and cofactor concentrations, pH and other metabolite effectors.

### TFs associated with transcriptionally regulated genes

To identify the potential TFs involved in the transcriptional control of the transcriptionally regulated reactions, we performed promoter analysis of the corresponding genes. In total, promoter sequences for 26 transcriptionally up-regulated genes and 27 down-regulated genes were used for *cis*-element detection **[see Supporting Information]**. This analysis identified several highly enriched putative *cis*-elements associated with different potential TFs for both up- and down-regulated transcriptionally controlled genes (Tables 1 and 2). A high enrichment of putative *cis*-elements such as AT-hook/PE-1-like, GT element-like, pyrimidine-box-like, GARE-like and MYb-box-like associated with MYB TFs was observed among both up- and down-regulated genes. However, the high enrichment of these *cis*-elements in the up-regulated genes signifies that MYB TFs especially play an important role in the transcriptional control of up-regulated genes under anoxia. Similarly, putative *cis*-elements such as AS-1/ocs-like and ABRE-like associated with bZIP TFs were also found in both up- and down-regulated genes but with a high percentage of occurrences only in up-regulated genes. Besides, a number of other putative *cis*-elements such as ERE-like/GCC-box-like and zinc finger binding element-like associated with ERF and ZnF TFs were also more overrepresented in the up-regulated genes albeit being present in both up- and down-regulated genes. Furthermore, we noticed a moderate enrichment of other ERE like-elements such as JA response element-like elements among the up-regulated genes (Table 1). Collectively, these results indicate that all these TFs, i.e. MYB, bZIP, ERF and ZnF, may work together in the transcriptional control of the up-regulated genes in rice central metabolism. Interestingly, we also noticed the enrichment of MBF1C binding element and CRT/DRE-like elements associated with MBF1C and CBF/DREB TFs only in down-regulated genes. This highlights the possibility that this TF could be involved in transcriptional control of down-regulated genes under anoxia (Table 2).

**Table 1.** Potential *cis*-elements identified in the promoters of transcriptionally controlled up-regulated genes of rice seeds germinated under anoxia.

*Cis*-elements	Motifs	Associated TFs	% (TIC), e value
AT-hook/PE1-like	TTTTTTCA	MYB (PF1)	73 (12.78), 2e−004
	AATTTTTTT	MYB (PF1)	65 (16.05), 3e−005
	ATAAAAAAAA	MYB (PF1)	58 (16.67), 0e+000
	AAGAAAAAG	MYB (PF1)	58 (13.91), 1e−004
	AAAAATAC	MYB (PF1)	54 (13.32), 1e−004
	TTTTTTCTTT	MYB (PF1)	50 (16.74), 3e−005
GT-element-like	TGGTTTGT	MYB (GT-1/GT-3b)	81 (12.15), 1e−004
	TTTTTTCA	MYB (GT-1/GT-3b)	73 (12.78), 2e−004
	GGCTTGTG	MYB (GT-1/GT-3b)	69 (11.62), 2e−000
	AGGAAAAAG	MYB (GT-1/GT-3b)	58 (13.91), 1e−004
	AAATCATA	MYB (GT-1)	62 (12.80), 8e−005
	AAATCAAAT	MYB (GT-1)	62 (13.69), 1e−004
	TTTTTTCTTT	MYB (GT-1)	50 (16.74), 3e−005
Pyrimidine-box-like	TTTTTTCA	MYB (R1, R2R3)	73 (12.78), 2e−004
	CTTTTGCT	MYB (R1, R2R3)	65 (12.33), 9e−005
GARE-like	AAAACAAA	MYB (R1, R2R3)	58 (12.66), 2e−004
MYB-box-like	TGGTTTAT	MYB (R2R3)	81 (12.15), 1e−004
	TGGTTTGT	MYB (R2R3)	81 (12.15), 1e−004
	AACTTGTT	MYB (R2R3)	54 (13.18), 3e−005
As-1/ocs-like	TTTTTTCA	bZIP (Gr. D, I, S)	73 (12.78), 2e−004
	AAATCATA	bZIP (Gr. D, I, S)	62 (12.80), 8e−005
	ATGAAAAAG	bZIP (Gr. D, I, S)	58 (13.91), 1e−004
ABRE-like	AAATCAAAT	bZIP (Gr. A)	62 (12.80), 8e−005
	CTTTGCCA	bZIP (Gr. A)	58 (13.43), 1e−004
	GAGCGCCA	bZIP (Gr. A)	54 (12.18), 3e−004
RSG binding element-like	AACTTGTT	bZIP	54 (13.18), 3e−005
CAMTA3 binding site-like	GAAGAAAA	bZIP	63 (14.30), 2e−004
RISbZ1 binding site-like	AAAACAAA	bZIP (RISbZ1)	58 (12.66), 2e−004
CAMTA3 binding site-like	GAGAAAGAA	bZIP	58 (14.52), 1e−004
	AAGAAGAG	bZIP	50 (13.41), 2e–004
Zinc finger binding element-like	AAGAAGAG	ZnF (ZCT1, ZCT2, ZCT3)	62 (13.69), 1e−004
	AAATCATA	ZnF (ZCT1, ZCT2, ZCT3)	62 (12.80), 8e−005
	AAATCAAAT	ZnF (ZCT1, ZCT2, ZCT3)	50 (13.41), 2e−004
ERE-like (JA response element-like)	AAATCATA	ERF (Gr. VI, VIII, IX)	62 (12.80), 8e−005
	AAATCAAAT	ERF (Gr. VI, VIII, IX)	50 (13.41), 2e−004
GCC-box-like	CTCCGCCGC	ERF (I, IV, VII, X)	50 (15.38), 2e−005
AuxRe-like	CTTTTGCT	ARF	65 (12.33), 9e−005
	CTTTGCCA	ARF	58 (13.43), 1e−004
AAAAG/element-like	CTTTTGCT	DOF (Dof1/4/11/22)	65 (12.33), 9e−005
	AAGAAAAAG	DOF (Dof1/4/11/22)	58 (13.43), 1e−004
MYC-box-like	TGCTACTC	bHLH (JAMYC2)	65 (11.96), 1e−004
ARR10 binding element-like	AAATCATA	ARR-B (ARR10)	62 (13.69), 1e−004
TATA-box-like	TATAAATT	TBP	96 (12.32), 3e−005
DBP element-like	AAAAATAC	DBP	54 (13.32), 1e−004
DBP1 element-like	AATATATTA	DBP1	50 (15.09), 8e−005

**Table 2.** Potential *cis*-elements identified in the promoters of transcriptionally controlled down-regulated genes of rice seeds germinated under anoxia.

*Cis*-elements	Motifs	Associated TFs	% (TIC), e value
AT-hook/PE1-like	ATATTTTTAT	MYB (PF1)	59 (16.39), 6e−005
	TTTAAAAAA	MYB (PF1)	59 (16.42), 2e−005
GT-element-like	ATTGGCTA	MYB (GT-1)	56 (12.42), 2e−004
MYB-box-like	AAAATCCA	MYB (R2R3, MCB1/2)	70 (13.11), 2e−004
As-1/ocs-like	TCGTCGCG	bZIP (Gr. D, I, S)	63 (13.21), 0e+000
	ACGTGTCA	bZIP (Gr. D, I, S)	59 (11.79), 3e−004
	AGACGTTG	bZIP (Gr. D, I, S)	56 (11.91), 3e−005
ABRE-like	ACGTGACA	bZIP (Gr. A)	59 (11.79), 3e−004
	TCGCCGGC	bZIP (Gr. A)	59 (13.20), 5e−005
ER stress RE-like	ATTGGCTA	bZIP (Gr. D)	56 (12.42), 2e−004
RISbZ1 binding site-like	AAAACAAA	bZIP (RISbZ1)	58 (12.66), 2e−004
AuxRe-like	ACTACTAT	ARF1	67 (12.28), 9e−005
	TCGTCGCG	ARF1	63 (13.21), 0e+000
	ACGTGACA	ARF1	59 (11.79), 3e−004
	AATCCTTT	ARF1	56 (13.21), 9e−005
GAGA element-like	CTCCTCTC	GAGA-binding factor BBR/BPC2	63 (14.39), 7e−004
	TCCTCTAT	GAGA-binding factor BBR	52 (13.62), 4e−004
	GGGAGAGGG	GAGA-binding factor BBR	52 (15.63), 3e−005
DBP1 element-like	TTTATTTT	DBP1	85 (13.63), 2e−004
	ACATTAAA	DBP1	78 (12.88), 2e−004
	AAATAATA	DBP1	62 (13.44), 9e−005
GCC-box-like	GGCGGCGGC	ERF (I, IV, VII, X)	70 (15.92), 1e−004
	CCGCCGCC	ERF (I, IV, VII, X)	56 (13.95), 3e−004
ARR10 binding element-like	AAAATCCA	ARR-B (ARR10)	70 (13.11), 2e−004
	AATCCTTT	ARR-B (ARR10, ARR5, ARR1)	56 (13.21), 9e−005
CRT/DRE-like	TCGTCGCG	CBF1/DREB	63 (13.21), 0e+000
AAAGG element-like	AATCCTTT	DOF	56 (13.21), 9e−005
	ATTTAAAGA	DOF (Dof1/4/11/22)	52 (14.11), 9e−005
Zinc finger binding element-like	GAGGAGGAG	ZnF	56 (16.04), 6e−005
MBF1C binding element-like	GAGGAGGAG	MBF1C	56 (16.04), 6e−005
TATA-box-like	TTTTATATA	TBP	63 (15.28), 2e−004
DBP element-like	ATATTTTTAT	DBP	59 (16.39), 6e−005

### Motif detection in negative sets

To ensure that our *cis*-element analysis did not identify an excessive number of false positives, we sought motifs from the promoters of a similar number of genes that are not anoxia specific using the same protocol (see Methods). For this purpose, we used two negative datasets: (i) randomly selected genes from the non-differentially expressed gene list under anoxia (Lasanthi-Kudahettige *et al.* 2007) (negative Set 1) and (ii) up-regulated genes associated with drought response in rice (Zhang *et al.* 2012) (negative Set 2). The list of genes from the negative sets and the results of this analysis are provided in **Supplementary Information**. Using the negative control Set 1, we identified a few common motifs such as MYB, bZIP and DOF, and their total enrichment was much lower compared with the motifs detected in transcriptionally up- or down-regulated genes of anoxia (Tables 1, 2 and **Supplementary Information**). Similarly, the motif analysis of negative Set 2 also revealed a significantly different *cis*-element enrichment pattern where most of the identified TFs such as MYB, bZIP, ERF, NAC and MYC have been experimentally confirmed to play a regulatory role in drought stress response (Shinozaki and Yamaguchi-Shinozaki 2007; Zhang *et al.* 2012). Collectively, these results clearly demonstrate that our *cis*-element method identifies reasonably precise motifs that are specific to anoxic stress.

## Discussion

Our study analysed the plausible metabolic states of rice under aerobic and anaerobic conditions using random sampling and compared their differences. We also compared the changes in flux levels of individual enzymes in the central metabolism of rice grown in the two conditions with that of gene expression and identified the transcriptionally controlled reactions. Accordingly, the major contribution of the current work is the demonstration of the utility of random flux sampling and gene expression data for elucidating metabolic differences and the identification of corresponding putative TFs responsible for such changes under the stress conditions.

Monte Carlo flux sampling has been shown to be an effective tool to analyse and compare the plausible solution space of a metabolic network across different environmental/genetic conditions (Famili *et al.* 2003; Bordel *et al.* 2010; Osterlund *et al.* 2013). When compared with the commonly used constraint-based analysis methods such as flux balance analysis (FBA) and minimization of metabolic adjustment, which predict just a single flux distribution based on a particular objective function, random sampling allows us to account for uncertainty in the flux distributions as it uniformly samples the possible solution space in an unbiased manner and, thus, potentially minimizes the possibility of overestimating the actual flux differences between the two conditions. This can be clearly exemplified by comparing the results of previous work (Lakshmanan *et al.* 2013), which utilized FBA for simulating flux differences between air and anoxia, with our current results. Although both studies show that most of the central metabolic reactions possess different flux levels in aerobic and anaerobic conditions, flux sampling can provide confidence scores to the reactions, thus allowing us to consider the reactions with the most plausible fluxes. Furthermore, by comparing the significant flux differences with gene expression data, we determined the reactions that are most probably regulated at the transcriptional level. Most importantly, we revealed that although the glycolytic reactions are not transcriptionally regulated, genes coding for reactions upstream and downstream of glycolysis, i.e. sucrose metabolism and fermentation, respectively, are regulated transcriptionally. It will be important now to compare and contrast such findings with anoxia-intolerant higher plants such as *Arabidopsis thaliana*, wheat and maize to analyse whether the corresponding gene orthologues are commonly regulated at a similar level.

In recent years, *in silico* promoter analysis of differentially expressed genes from microarray data has improved our understanding of the transcriptional regulation of gene expression markedly. Several *in silico* analyses on the promoter *cis*-elements and their cognate TFs have been experimentally verified for predicted functions, highlighting its reliability (Meier *et al.* 2008; Yun *et al.* 2010; Stamm *et al.* 2012). Therefore, in this study, we analysed the *cis*-regulatory content of transcriptionally controlled reactions through such a method and unravelled the possible involvement of a number of TFs in transcriptional control of sucrose metabolism and fermentation under anoxia. Among all the putative *cis*-elements identified, the elements associated with MYB family TFs were found to be highly enriched in transcriptionally up-regulated genes. Coincident with these observations a number of MYB family genes are also up-regulated under anoxia (Table 3). MYB proteins represent important plant TFs and are found to be involved in various developmental and physiological processes including transcriptional activation, kinase activity, protein binding and transcription repressor activation under abiotic and biotic stresses (Dubos *et al.* 2010). In rice, a recent analysis highlighted that 98.70 % of total MYB proteins are fully involved in transcriptional activation (Katiyar *et al.* 2012). Furthermore, the role of MYB TFs that bind specifically to MYB box/GT element during hypoxic and anoxic responses has already been reported through promoter analysis, both computationally and experimentally (Dolferus *et al.* 2003; Mohanty *et al.* 2005, 2012). Specifically, the *AtMYB2* in Arabidopsis has been shown to be a key regulator of the *ADH1* promoter under low oxygen conditions (Hoeren *et al.* 1998). When this *AtMYB2* was driven by a constitutive promoter, it was able to transactivate *ADH1* expression not only in Arabidopsis but also in *Nicotiana plumbaginifolia* and *Pisum sativum*. Collectively, these results fully support the hypothesis that MYB TFs play an important role in the up-regulation of sucrose metabolism and fermentation enzymes at the transcriptional level.

**Table 3.** List of anoxia-stressed up-regulated TFs with potential significance to the pattern of *cis*-element enrichment among the up-regulated genes.

Family	Locus_ID (Annotation)	Fold increase
MYB/MYB-related	Os02g0706400 (Myb-related, similar to Radialis)	9.0
	Os06g0728700 (Homeodomain-like protein)	7.0
	Os08g0151000 (Myb-like, SHAQKYF class)	7
	Os01g0524500 (Myb-like, SHAQKYF class)	6
	Os01g0863300 (similar to MCB2 protein)	4
	Os08g0549000 (similar to MybHv5)	3
	Os05g0459000 (c-Myb protein)	2
	Os04g0480300 (Myb-like protein)	2
bZIP	Os09g0306400 (bZIP-1 domain protein)	16.0
	Os03g0336200 (RF2b transcription factor)	6.0
	Os06g0662200 (bZIP-1 domain protein)	4.0
	Os01g0867300 (G-box binding factor)	3.0
	Os05g0489700 (similar to BZO2H3)	2.0
	Os05g0129300 (bZIP protein)	2.0
	Os05g0569300 (G-box binding factor)	2.0
ERF	Os03g0341000 (similar to RAP2.2)	29.0
	Os01g0131600 (similar to PTI6, pathogenesis-related)	3.0
	Os06g0604000 (similar to ERF1 and ERF3)	3.0
ZnF	Os05g0525900 (similar to Zinc finger transcription factor PEI1)	21.0
	Os09g0560900 (zinc finger, C2H2-like domain containing protein)	2.0
	Zinc finger, CCCH-type domain containing protein. (Os04t0663200-01) (similar to OSIGBa0099L20.3 protein)	2.0
	Os02g0672100 (zinc finger, C2H2-type domain containing protein)	2.0
	Os09g0560900 (zinc finger, C2H2-like domain containing protein)	2.0
ARF	Os04g0671900 (similar to auxin response factor)	2.0
	Os06g0677800 (similar to auxin response factor)	2.0
DOF	Os05g0112200 (Dof domain, zinc finger family protein, expressed)	2.0
bHLH	Os11t0523700 (similar to transcription factor ICE1 (Inducer of CBF expression 1) (basic helix–loop–helix protein 116) (bHLH116)	3.0
	Os02t0433600 (helix–loop–helix DNA-binding domain containing protein)	2.0
Pseudo-ARR-B	Os11t0157600 (similar to timing of CAB expression)	3.0

As mentioned above, our promoter analysis also highlighted significant overrepresentation of several other motifs associated with bZIP, ERF and ZnF TFs in the transcriptionally up-regulated genes. In this regard, an increase in transcript levels of bZIP TF (*AtbZIP50*) in anoxia-exposed root cultures of Arabidopsis (Klok *et al.* 2002), the up-regulation of an ABRE-binding bZIP TF (*OsABF1*) in rice shoot and root under anoxic treatment (Amir Hossain *et al.* 2010) and the up-regulation of a number of bZIP TFs in rice anoxic coleoptile (Table 3) support the view that bZIP could orchestrate the transcription of up-regulated genes upon anoxic stress. The major role of ERF TF has been identified as a positive regulator of Sub1A expression in a flood-tolerant rice variety during hypoxic conditions caused by submergence (Xu *et al.* 2006). However, the anoxia-tolerant rice variety ‘*Nipponbare*’ that germinates and elongates under anoxia does not have this gene. Surprisingly, the presence of highly enriched putative GCC-box-like/ERE-like *cis*-elements in the promoters of all anoxia up-regulated genes in this variety of rice highlights the possiblity that these TFs might also control the transcription of relevant genes as it is in Arabidopsis under hypoxic conditions (Hinz *et al.* 2010; Licausi *et al.* 2010, 2011). Unlike the ERF TFs, the role of ZnF TF in response to submergence/anoxia is not yet known. However, matching with our identification of ZnF binding site-like elements in transcriptionally up-regulated genes, the increase in expression of ZnF TF genes (Table 3) in response to hypoxia/anoxia in both rice and Arabidopsis allows us to speculate on its potential regulatory role in transcriptional control under oxygen stress (Loreti *et al.* 2005; Pandey and Kim 2012).

**Figure 2.**
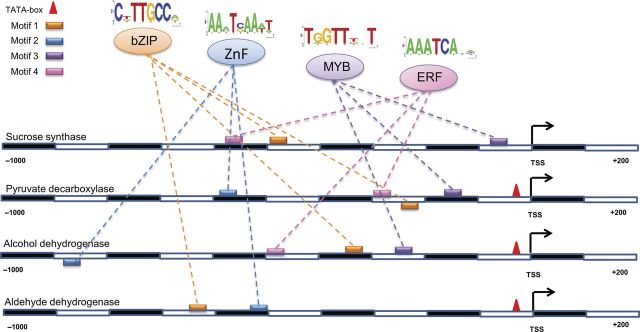
Presence of common putative *cis*-elements in the key transcriptionally regulated genes. The presence of potential putative *cis*-elements and their cognate known TFs are shown in different strands of the promoter region (−1000, +200 nt relative to TSS) of the key genes. Each putative *cis*-element/motif is represented by its consensus logo. TATA boxes are located between 25 and 30 nt upstream from the TSS.

As mentioned earlier, we utilized the promoter sequences of 26 transcriptionally up-regulated and 27 down-regulated genes for the *cis*-element detection in this study. When compared with the normal promoter analysis method that considers the whole set of differentially expressed genes, the current study takes into account relatively few genes. Therefore, in order to argue that this small set of genes is sufficient to generate biologically meaningful results, we first compared the TFs identified in this study with previous work that considered the whole list of up-regulated (842) and down-regulated (1794) genes during anoxic adaptation (Mohanty *et al.* 2012). Interestingly, the comparison result revealed that the TFs associated with up- and down-regulated genes are the same between two studies where the motif enrichment scores of certain TFs such as MYB, bZIP, ZnF and ERF are much higher in the current study, highlighting the possible role of these TFs in anoxic adaptation. To further confirm our findings, we also analysed the individual promoter sequences of four of the key transcriptionally regulated enzymes, i.e. SUS, PDC, ADH and ALDH, and identified the same TFs as fully responsible for their transcription (Fig. 2). Therefore, based on these motif analysis results and experimental evidence from the literature, we hypothesize a positive role of MYB together with bZIP, ERF and ZnF in the transcriptional control of sucrose metabolism and fermentation during germination and coleoptile elongation of rice under anoxia.

The current methods we used successfully identified several transcriptionally regulated reactions and their related TFs, some of which are experimentally confirmed. However, the overall results of this approach await experimental validation since the current prediction relies on several assumptions concerning model completeness, constraints used during simulation and the statistical cut-off values chosen for comparative analysis. In our work, we utilized the central model to simulate the differences in metabolic fluxes between air and anoxia. It should be noted that although this model predicts the overall cellular phenotype quite accurately (Lakshmanan *et al.* 2013), it may not capture the global changes in cellular metabolism. This reservation is needed since the model does not take account of the secondary metabolic pathways. Moreover, we have utilized only a few external fluxes, i.e. sucrose uptake, O_2_ uptake and growth yield, as the sole constraints to identify the internal flux distributions during flux sampling simulations. In such cases, the internal fluxes of some reactions are determined with statistically low confidence scores due to the possibility of multiple flux solutions. Such limitations can be overcome by the use of C_13_ flux measurements as constraints to the internal reactions. Therefore, the list of transcriptionally regulated reactions and the TFs identified in this study need further confirmation by direct experiments.

## Conclusions

In this study, we presented a combined *in silico* modelling and gene expression data analysis framework to identify transcriptionally regulated reactions in rice central metabolism. Random flux sampling simulations highlighted significant changes in flux levels across oxidative phosphorylation, the TCA cycle, the pentose phosphate pathway, glycolysis, sucrose metabolism and fermentation. The subsequent comparative analysis of changes in flux levels and gene expression between aerobic conditions identified 37 reactions from these pathways that are regulated at the transcriptional level. The motif enrichment analysis of transcriptionally regulated enzymes highlighted the potential involvement of TFs MYB, bZIP, ERF and ZnF in controlling the transcription of sucrose metabolism and fermentation genes under anaerobic conditions. Thus, the current study successfully demonstrated how condition-specific gene expression data can be exploited to narrow down the metabolic changes in rice under anaerobic adaptations and *in silico* analysis to help us unravel the relevant transcriptional mechanisms.

## Sources of Funding

This work was supported by the National University of Singapore, the Competitive Research Programme of the National Research Foundation of Singapore (NRF-CRP5-2009-03), the Biomedical Research Council of A*STAR (Agency for Science, Technology and Research), Singapore, and a grant from the Next-Generation BioGreen 21 Program (SSAC, No. PJ009520), Rural Development Administration, Republic of Korea.

## Contributions by the Authors

M.L., B.M. and D.Y.L. conceived and designed the study. M.L. carried out flux sampling and gene expression data analysis. B.M. carried out the *ab initio* analysis of *cis*-regulatory elements. M.L., B.M. and D.Y.L. wrote the manuscript. S.H.L. and S.H.H. provided critical comments and helped in revising the manuscript. All authors read and approved the final manuscript.

## Conflicts of Interest Statement

None declared.

## Supporting Information

The following Supporting Information is available in the online version of this article –

**File S1.** Table. Rice central model with updated GPR associations.

**File S2.** Diagram. Sampling histogram of glycolysis, pentose phosphate pathway, TCA cycle, oxidative phosphorylation and sucrose metabolism under aerobic and anaerobic conditions.

**File S3.** Table. List of rice central metabolic reactions and their flux differences with *P* values. Expression patterns of corresponding gene louses and type of regulation, i.e. transcriptional or metabolic, are also listed.

**File S4.** Table. List of genes used in negative sets.

**File S5.** Table. Potential *cis*-elements identified in the promoters of negative sets.

Additional Information
