# Supplementary material for: Metabolic and transcriptional regulatory mechanisms underlying the anoxic adaptation of rice coleoptile
Source: AoB Plants. 2014 Jun 3;6:plu026. doi: 10.1093/aobpla/plu026 (PMC4077593; doi:10.1093/aobpla/plu026)
Supplement: Additional Information [file supp_6_plu026_index.html]

Metabolic and transcriptional regulatory mechanisms underlying the anoxic adaptation of rice coleoptile — Additional Information 

# Metabolic and transcriptional regulatory mechanisms underlying the anoxic adaptation of rice coleoptile

## Additional Information

Additional Information

**Files in this Data Supplement:**

- Supplemental File S1 - doc file
- Supplemental File S2 - doc file
- Supplemental File S3 - doc file
- Supplemental File S4 - doc file
- Supplemental File S5 - doc file
