# Supplementary material for: Metabolic and transcriptional regulatory mechanisms underlying the anoxic adaptation of rice coleoptile
Source: AoB Plants. 2014 Jun 3;6:plu026. doi: 10.1093/aobpla/plu026 (PMC4077593; doi:10.1093/aobpla/plu026)
Supplement: Additional Information [file supp_plu026_plu026supp_data1.doc]

**Supplemental File S1**

# Combined in silico metabolic flux sampling and microarray data analysis reveals key transcriptional mechanisms in anoxic adaptation of rice coleoptile

Meiyappan Lakshmanan, Bijayalaxmi Mohanty, Sun-Hyung Lim, Sun-Hwa H3 and Dong-Yup Lee

Department of Chemical and Biomolecular Engineering, National University of Singapore, Singapore.

**Updated central metabolic model of Rice**

**Reactions List**

| **Reaction ID** | **Reaction Name** | **Reaction Equation** | **Updated Gene Locus** | **Compartment** | **EC Number** | **Subsystem** |
| --- | --- | --- | --- | --- | --- | --- |
| **Coleoptile_Biomass** | Coleoptile Biomass reaction | 0.0914 Ala_c + 0.0711 Arg_c + 0.0985 Asp_c + 0.0126Cys_c + 0.1758 Glu_c + 0.0940 Gly_c + 0.0209 His_c + 0.0450 Ile_c + 0.0901 Leu_c + 0.0409 Lys_c + 0.0251 Met_c + 0.0410 Phe_c + 0.0638 Pro_c + 0.0699 Ser_c + 0.0478 Thr_c + 0.0120 Trp_c + 0.0386 Tyr_c + 0.0750 Val_c + 0.0283 a-Glc_c + 0.5716 Sucrose_c + 0.0181 Fruct_c + 1.9304 Starch_c + 0.7401 cellulose_c + 0.0540 UDP-L-arab_c + 0.2641 UDP-Xyl_c + 0.0099 UDP-Gal_c + 0.1728 UDP-Glc_c + 0.2165 UDP-Glucur_c + 0.11494 TAG_c + 40.98 ATP_c -> Biomass_c + 40.98 ADP_c + 40.98 pi_c + 0.6953 UDP_c + 0.6953 H+_c |  |  |  | Biomass reactions |
| **Straw_Biomass** | Straw Biomass reaction | 0.0321 Ala[c] + 0.0123 Arg[c] + 0.0417 Asp[c] + 0.0033 Cys[c] + 0.0385 Glu[c] + 0.0282 Gly[c] + 0.057 His[c] + 0.0140 Ile[c] + 0.0234 Leu[c] + 0.0153 Lys[c] + 0.0076 Met[c] + 0.0133 Phe[c] + 0.0225 Pro[c] + 0.0219 Ser[c] + 0.0192 Thr[c] + 0.0016 Trp[c] + 0.0077 Tyr[c] + 0.0225 Val[c] + 0.0546 a-Glc[c] + 0.1049 Sucrose[c] + 0.0643 Fruct[c] + 0.3249 Starch[p] + 2.0789 cellulose[c] + 0.1256 UDP-L-arab[c] + 0.5720 UDP-Xyl[c] + 0.0229 UDP-Gal[c] + 0.4018 UDP-Glc[c] + 0.5033 UDP-Glucur[c] + 0.2065 Coum-Alc[c] + 0.1721 Conalc[c] + 0.1475 SinapAlc[c] + 0.01625 TAG[c] + 41.75 ATP[c] -> 41.75 ADP[c] + 41.75 pi[c] + 1.6256 UDP[c] + 1.6256 H+[c] |  |  |  | Biomass reactions |
| **Ex-CO2** | Carbon dioxide exchange | CO2[e] <-> CO2[c] |  |  |  | Exchange Reactions |
| **Ex-H2O** | Water exchange | H2O[e] <-> H2O[c] |  |  |  | Exchange Reactions |
| **Ex-photon** | Photon exchange | photon[e] -> photon[p] |  |  |  | Exchange Reactions |
| **Ex-H+** | Proton exchange | H+[e] <-> H+[c] |  |  |  | Exchange Reactions |
| **Ex-O2** | Oxygen exchange | O2[e] <-> O2[c] |  |  |  | Exchange Reactions |
| **Ex-pp** | Phosphate exchange | pi[e] <-> pi[c] |  |  |  | Exchange Reactions |
| **Ex-Sucrose** | Sucrose exchange | Sucrose[e] <-> Sucrose[c] |  |  |  | Exchange Reactions |
| **Ex-Glucose** | Glucose Exchange | a-Glc[e] <-> a-Glc[c] |  |  |  | Exchange Reactions |
| **Ex-Starch** | Starch exchange | Starch[e] <-> Starch[p] |  |  |  | Exchange Reactions |
| **Ex-Sulfate** | Sulfate exchange | sulfate[e] <-> sulfate[c] |  |  |  | Exchange Reactions |
| **Ex-Asparagine** | Asparagine exchange | Asn[e] <-> Asn[c] |  |  |  | Exchange Reactions |
| **Ex-Glutamine** | Glutamine exchange | Gln[e] <-> Gln[c] |  |  |  | Exchange Reactions |
| **Ex-ethanol** | Ethanol exchange | Ethanol[c] <-> Ethanol[e] |  |  |  | Exchange Reactions |
| **Ex-Lactate** | Lactate exchange | Lactate[c] <-> Lactate[e] |  |  |  | Exchange Reactions |
| **Ex-Alanine** | Alanine exchange | Ala[c] <-> Ala[e] |  |  |  | Exchange Reactions |
| **Ex-Succinate** | Succinate exchange | Succ[c] <-> Succ[e] |  |  |  | Exchange Reactions |
| **IPP[c]** | Inorganic pyrophosphatase | H2O[c] + ppi[c] -> 2 pi[c] + H+[c] | (LOC_Os01g64670 or LOC_Os02g47600 or LOC_Os04g59040 or LOC_Os05g02310 or LOC_Os05g36260 or LOC_Os10g26600) | cytosol | 3.6.1.1 | Sucrose metabolism; Oxidative phosphorylation |
| **IPP[m]** | Inorganic pyrophosphatase | H2O[m] + ppi[m] -> 2 pi[m] + H+[m] | (LOC_Os01g64670 or LOC_Os02g47600 or LOC_Os04g59040 or LOC_Os05g02310 or LOC_Os05g36260 or LOC_Os10g26600) | mitochondrion | 3.6.1.1 | Sucrose metabolism; Oxidative phosphorylation |
| **IPP[p]** | Inorganic pyrophosphatase | H2O[p] + ppi[p] -> 2 pi[p] + H+[p] | LOC_Os02g52940 | plastid | 3.6.1.1 | Sucrose metabolism; Oxidative phosphorylation |
| **ALS1[p]** | Acetolactate synthase | 2 Pyr[p] + H+[p] -> 2Acelac[p] + CO2[p] | (LOC_Os02g30630 or LOC_Os02g39570) | plastid | 2.2.1.6 | Valine biosynthesis |
| **CAT[c]** | Catalase | 2 H2O2[c] -> 2 H2O[c] + O2[c] | (LOC_Os08g43560 or LOC_Os04g14680 or LOC_Os02g02400 or LOC_Os06g51150) | cytosol | 1.11.1.6 | Photorespiration |
| **GS[p]** | Glutamate synthase (ferredoxin) | 2 redferr[p] + aKG[p] + Gln[p] + 2 H+[p] -> 2 oxiferr[p] + 2 Glu[p] | LOC_Os07g46460 | plastid | 1.4.7.1 | GS-GOGAT Cycle |
| **RBCS-C[p]** | Ribulose-bisphosphate carboxylase | RuBP[p] + CO2[p] + H2O[p] -> 2 3PG[p] + 2 H+[p] | (LOC_Os01g58020 and LOC_Os05g35330 and LOC_Os11g32770 and LOC_Os12g10580 and LOC_Os10g21280) and (LOC_Os12g19394 and LOC_Os02g05830 and LOC_Os12g17600 and LOC_Os12g19381 and LOC_Os12g19470) | plastid | 4.1.1.39 | Calvin cycle |
| **COX[m]** | Cytochrome c oxidase (complex IV) | 4 Cyto-Red[m] + O2[m] + 6 H+[m] -> 4 Cyto-Oxi[m] + 2 H2O[m] + 6 H+[c] | (LOC_Osm1g00550 and LOC_Osm1g00330 and LOC_Osm1g00110 and LOC_Os01g42650 and LOC_Os08g38720 and LOC_Os03g50940) | mitochondrion | 1.9.3.1 | Oxidative phosphorylation |
| **ATPPH[c]** | ATP phosphohydrolase | ATP[c] + H2O[c] -> ADP[c] + pi[c] + H+[c] | (LOC_Os01g49000 or LOC_Os06g03940) | cytosol | 3.6.1.3 | Purine metabolism |
| **ATPS[m]** | ATP synthase (complex V) | ADP[m] + pi[m] + 3 H+[c] -> ATP[m] + H2O[m] + 2 H+[m] | (((LOC_Os05g47980 and LOC_Os07g31300 and LOC_Os08g15170 and LOC_Os08g37320 and LOC_Os10g17280) and (LOC_Osm1g00580 and LOC_Osm1g00370 and LOC_Osm1g00430)) | mitochondrion | 3.6.3.14 | Oxidative phosphorylation |
| **GLT[p]** | Glutamate synthase (NADH) | NADH[p] + aKG[p] + Gln[p] + H+[p] -> NAD+[p] + 2 Glu[p] | LOC_Os01g48960 | plastid | 1.4.1.14 | GS-GOGAT Cycle |
| **GRC2[p]** | Glutathione reductase (NADPH) | GludiS[p] + NADPH[p] + H+[p] -> 2 Gluta[p] + NADP+[p] | (LOC_Os03g06740 or LOC_Os10g28000) | plastid | 1.8.1.7 | Sulfate assimilation |
| **ADNK[c]** | Adenylate kinase | AMP[c] + ATP[c] -> 2 ADP[c] | (None or LOC_Os12g13380) | cytosol | 2.7.4.3 | Purine metabolism |
| **ADNK[p]** | Adenylate kinase | AMP[p] + ATP[p] -> 2 ADP[p] | (LOC_Os03g03820 or LOC_Os07g22950 or LOC_Os08g01770 or LOC_Os08g19140) | plastid | 2.7.4.3 | Purine metabolism |
| **NDKR1[c]** | nucleoside-diphosphate kinase | UDP[c] + ATP[c] <-> UTP[c] + ADP[c] | (LOC_Os10g41410 or LOC_Os02g35700 or LOC_Os12g36194 or LOC_Os05g51700) | cytosol | 2.7.4.6 | Pyrimidine metabolism |
| **NDKR1[p]** | nucleoside-diphosphate kinase | UDP[p] + ATP[p] <-> UTP[p] + ADP[p] | (LOC_Os07g30970 or LOC_Os12g36194 or LOC_Os05g51700) | plastid | 2.7.4.6 | Pyrimidine metabolism |
| **ATPT[p]** | ATP:UMP phosphotransferase | ATP[p] + UMP[p] -> ADP[p] + UDP[p] |  | plastid | 2.7.4.4 | Pyrimidine metabolism |
| **SAM[c]** | Methionine adenosyltransferase | ATP[c] + Met[c] + H2O[c] -> pi[c] + ppi[c] + S-Ade-L-meth[c] | (LOC_Os01g18860 or LOC_Os07g29440 or LOC_Os06g11180 or LOC_Os01g22010 or LOC_Os05g04510 or LOC_Os01g10940) | cytosol | 2.5.1.6 | Methionine biosynthesis |
| **AMPP[c]** | Adenosine 5'-monophosphate phosphohydrolase | AMP[c] + H2O[c] -> Adenosine[c] + pi[c] | (LOC_Os01g51280 or LOC_Os03g44660 or LOC_Os07g10460) | cytosol | 3.1.3.5 | Purine metabolism |
| **ADK[c]** | Adenosine kinase | Adenosine[c] + ATP[c] -> AMP[c] + ADP[c] | (LOC_Os02g41590 or LOC_Os04g43750) | cytosol | 2.7.1.20 | Purine metabolism |
| **SAHH[c]** | Adenosylhomocysteinase | S-Ade-L-H[c] + H2O[c] -> HomoCys[c] + Adenosine[c] | LOC_Os11g26850 | cytosol | 3.3.1.1 | Methionine biosynthesis |
| **PPS[c]** | Pyruvate,water dikinase | H2O[c] + Pyr[c] + ATP[c] -> pi[c] + PEP[c] + AMP[c] |  | cytosol | 2.7.9.2 | Glycolysis/Gluconeogensis |
| **PPS[p]** | Pyruvate,water dikinase | H2O[p] + Pyr[p] + ATP[p] -> pi[p] + PEP[p] + AMP[p] |  | plastid | 2.7.9.2 | Glycolysis/Gluconeogensis |
| **PYK[c]** | Pyruvate kinase | ADP[c] + PEP[c] + H+[c] -> ATP[c] + Pyr[c] | (LOC_Os01g16960 or LOC_Os03g20880 or LOC_Os11g10980) | cytosol | 2.7.1.40 | Glycolysis/Gluconeogensis |
| **PYK[p]** | Pyruvate kinase | ADP[p] + PEP[p] + H+[p] -> ATP[p] + Pyr[p] | (LOC_Os01g16960 or LOC_Os01g47080 or LOC_Os03g20880 or LOC_Os03g46910 or LOC_Os07g08340 or LOC_Os10g42100 or LOC_Os11g05110 or LOC_Os11g10980 or LOC_Os12g05110) | plastid | 2.7.1.40 | Glycolysis/Gluconeogensis |
| **PPDK1[p]** | Pyruvate phosphate dikinase | ppi[p] + PEP[p] + AMP[p] <-> ATP[p] + Pyr[p] + pi[p] | LOC_Os03g31750 | plastid | 2.7.9.1 | Glycolysis/Gluconeogensis |
| **PPDK2[c]** | Pyruvate phosphate dikinase | ppi[c] + PEP[c] + AMP[c] <-> ATP[c] + Pyr[c] + pi[c] | LOC_Os05g33570 | cytosol | 2.7.9.1 | Glycolysis/Gluconeogensis |
| **PFL[c]** | Formate C-acetyltransferase | CoA[c] + Pyr[c] -> Formate[c] + Ace-CoA[c] |  | cytosol | 2.3.1.54 | Fermentation |
| **ME6[p]** | Malate dehydrogenase (oxaloacetate decarboxylating) (NADP+) | NADP+[p] + Malate[p] -> NADPH[p] + CO2[p] + Pyr[p] | (LOC_Os01g52500 or LOC_Os01g54030 or LOC_Os01g09320 or LOC_Os05g09440) | plastid | 1.1.1.40 | Photorespiration |
| **PDC[c]** | Pyruvate decarboxylase | Pyr[c] + H+[c] -> Acetald[c] + CO2[c] | (LOC_Os01g06660 or LOC_Os05g39310 or LOC_Os03g18220 or LOC_Os05g39320 or LOC_Os07g49250) | cytosol | 4.1.1.1 | Fermentation |
| **ACDH[c]** | Acetaldehyde dehydrogenase (acetylating) | NAD+[c] + CoA[c] + Acetald[c] <-> NADH[c] + Ace-CoA[c] + H+[c] | (LOC_Os11g08300 or LOC_Os09g26880 or LOC_Os04g45720 or LOC_Os02g43280 or LOC_Os02g43194) | cytosol | 1.2.1.10 | Fermentation |
| **ACS[c]** | acetate--CoA ligase | CoA[c] + Acetate[c] + ATP[c] -> Ace-CoA[c] + ppi[c] + AMP[c] | (LOC_Os02g32490 or LOC_Os04g33190) | cytosol | 6.2.1.1 | Fermentation |
| **ACS[m]** | acetate--CoA ligase | CoA[m] + Acetate[m] + ATP[m] -> Ace-CoA[m] + ppi[m] + AMP[m] |  | mitochondrion | 6.2.1.1 | Fermentation |
| **ACS[p]** | acetate--CoA ligase | CoA[p] + Acetate[p] + ATP[p] -> Ace-CoA[p] + ppi[p] + AMP[p] | LOC_Os02g32490 | plastid | 6.2.1.1 | Fermentation |
| **P5CS1[c]** | Glutamate 5-kinase | Glu[c] + ATP[c] -> L-Glut-5P[c] + ADP[c] |  | cytosol | 2.7.2.11 | Proline metabolism |
| **P5CS1[p]** | Glutamate 5-kinase | Glu[p] + ATP[p] -> L-Glut-5P[p] + ADP[p] | (LOC_Os01g55890 or LOC_Os01g73450) | plastid | 2.7.2.11 | Proline metabolism |
| **GDH1[m]** | Glutamate dehydrogenase (NADP+) | ammonia[m] + aKG[m] + NADPH[m] + H+[m] -> Glu[m] + H2O[m] + NADP+[m] | LOC_Os01g37760 | mitochondrion | 1.4.1.4 | Glutamine/Glutamate metabolism |
| **GDH2[m]** | Glutamate dehydrogenase (NAD+) | Glu[m] + H2O[m] + NAD+[m] -> ammonia[m] + aKG[m] + NADH[m] + H+[m] | (LOC_Os02g43470 or LOC_Os03g58040 or LOC_Os04g45970) | mitochondrion | 1.4.1.2 | Glutamine/Glutamate metabolism |
| **GLN1[c]** | Glutamate--ammonia ligase | ammonia[c] + Glu[c] + ATP[c] -> Gln[c] + ADP[c] + pi[c] | (LOC_Os02g50240 or LOC_Os03g12290 or LOC_Os03g50490) | cytosol | 6.3.1.2 | Glutamine/Glutamate metabolism |
| **GLN2[p]** | Glutamate--ammonia ligase | ammonia[p] + Glu[p] + ATP[p] -> Gln[p] + ADP[p] + pi[p] | LOC_Os04g56400 | plastid | 6.3.1.2 | Photorespiration |
| **ALAAT[c]** | Alanine aminotransferase | aKG[c] + Ala[c] <-> Glu[c] + Pyr[c] | (LOC_Os09g26380 or LOC_Os10g25130 or LOC_Os10g25140) | cytosol | 2.6.1.2 | Alanine biosynthesis |
| **GAT[c]** | Amino-acid N-acetyltransferase | Glu[c] + Ace-CoA[c] -> N-Ace-L-glutt[c] + CoA[c] | (LOC_Os03g31690 or LOC_Os07g39690) | cytosol | 2.3.1.1 | Arginine biosynthesis |
| **GAD[c]** | Glutamate decarboxylase | Glu[c] + H+[c] -> CO2[c] + GABA[c] | LOC_Os03g13300 | cytosol | 4.1.1.15 | GABA metabolism |
| **IDP[c]** | Isocitrate dehydrogenase (NADP+) | Isocit[c] + NADP+[c] -> NADPH[c] + aKG[c] + CO2[c] | (LOC_Os01g46610 or LOC_Os04g42920 or LOC_Os05g49760) | cytosol | 1.1.1.42 | TCA Cycle |
| **IDP[m]** | Isocitrate dehydrogenase (NADP+) | Isocit[m] + NADP+[m] -> NADPH[m] + aKG[m] + CO2[m] | (LOC_Os02g38200 or LOC_Os04g40310) | mitochondrion | 1.1.1.42 | TCA Cycle |
| **GPX[p]** | glutathione peroxidase | H2O2[p] + 2 Gluta[p] -> GludiS[p] + 2 H2O[p] | (LOC_Os04g46960 or LOC_Os02g44500 or LOC_Os06g08670) | plastid | 1.11.1.9 | Sulfate assimilation |
| **UGD[c]** | UDP-glucose 6-dehydrogenase | UDP-Glc[c] + H2O[c] + 2 NAD+[c] -> UDP-Glucur[c] + 2 NADH[c] + 3 H+[c] | (LOC_Os03g55070 or LOC_Os12g25700 or LOC_Os12g25690) | cytosol | 1.1.1.22 | Cell wall metabolism |
| **UGP[c]** | UTP--glucose-1-phosphate uridylyltransferase | a-Glc1P[c] + UTP[c] <-> UDP-Glc[c] + ppi[c] | (LOC_Os02g02560 or LOC_Os09g38030) | cytosol | 2.7.7.9 | Sucrose metabolism |
| **GALE[c]** | UDP-glucose 4-epimerase | UDP-Glc[c] <-> UDP-Gal[c] | (LOC_Os05g51670 or LOC_Os08g28730 or LOC_Os09g15420 or LOC_Os09g35800) | cytosol | 5.1.3.2 | Cell wall metabolism |
| **NDKR2[c]** | nucleoside-diphosphate kinase | GDP[c] + ATP[c] <-> GTP[c] + ADP[c] | (LOC_Os10g41410 or LOC_Os02g35700 or LOC_Os12g36194 or LOC_Os05g51700) | cytosol | 2.7.4.6 | Purine metabolism |
| **NDKR2[p]** | nucleoside-diphosphate kinase | GDP[p] + ATP[p] <-> GTP[p] + ADP[p] | (LOC_Os07g30970 or LOC_Os12g36194 or LOC_Os05g51700) | plastid | 2.7.4.6 | Purine metabolism |
| **PCKA[c]** | Phosphoenolpyruvate carboxykinase (ATP) | OAA[c] + ATP[c] -> CO2[c] + PEP[c] + ADP[c] | (LOC_Os03g15050 or LOC_Os10g13700 or LOC_Os04g50208) | cytosol | 4.1.1.49 | Glycolysis/Gluconeogensis |
| **MDH[c]** | Malate dehydrogenase | Malate[c] + NAD+[c] + H+[c] <-> OAA[c] + NADH[c] | LOC_Os10g33800 | cytosol | 1.1.1.37 | Glycolysis/Gluconeogensis |
| **MDH[m]** | Malate dehydrogenase | Malate[m] + NAD+[m] + H+[m] <-> OAA[m] + NADH[m] | (LOC_Os05g49880 or LOC_Os01g61380 or LOC_Os01g46070 or LOC_Os07g43700 or LOC_Os08g33720) | mitochondrion | 1.1.1.37 | TCA Cycle |
| **PPC[c]** | Phosphoenolpyruvate carboxylase | pi[c] + OAA[c] <-> PEP[c] + HCO3[c] | (LOC_Os01g02050 or LOC_Os08g43710 or LOC_Os01g55350 or LOC_Os01g11054 or LOC_Os09g14670 or LOC_Os08g27840 or LOC_Os02g14770 or LOC_Os01g73970) | cytosol | 4.1.1.31 | Glycolysis/Gluconeogensis |
| **CSY[m]** | Citrate synthase | OAA[m] + Ace-CoA[m] + H2O[m] -> Citrate[m] + CoA[m] + H+[m] | (LOC_Os02g10070 or LOC_Os11g33240) | mitochondrion | 2.3.3.1 | TCA Cycle |
| **ASP1[c]** | Aspartate aminotransferase | Asp[c] + aKG[c] <-> Glu[c] + OAA[c] | LOC_Os01g55540 | cytosol | 2.6.1.1 | Aspartate biosynthesis |
| **ASP1[m]** | Aspartate aminotransferase | Asp[m] + aKG[m] <-> Glu[m] + OAA[m] | LOC_Os06g35540 | mitochondrion | 2.6.1.1 | Aspartate biosynthesis |
| **ASP1[p]** | Aspartate aminotransferase | Asp[p] + aKG[p] <-> Glu[p] + OAA[p] | (LOC_Os01g65090 or LOC_Os02g55420) | plastid | 2.6.1.1 | Aspartate biosynthesis |
| **GGAT[c]** | Glycine aminotransferase | Glu[c] + Glyoxylate[c] -> aKG[c] + Gly[c] | LOC_Os07g01760 | cytosol | 2.6.1.4 | Photorespiration |
| **SUCLG[m]** | Succinate--CoA ligase (ADP-forming) | Succ[m] + CoA[m] + ATP[m] <-> Suc-CoA[m] + ADP[m] + pi[m] | LOC_Os02g40830 | mitochondrion | 6.2.1.5 | TCA Cycle |
| **LYSA[p]** | Diaminopimelate decarboxylase | mPime[p] + H+[p] -> CO2[p] + Lys[p] | LOC_Os05g46360 | plastid | 4.1.1.20 | Lysine biosynthesis |
| **MLS[p]** | Malate synthase | Ace-CoA[p] + H2O[p] + Glyoxylate[p] -> Malate[p] + CoA[p] + H+[c] | LOC_Os04g40990 | plastid | 2.3.3.9 | Photorespiration |
| **GOX[c]** | glycolate oxidase | Glycolate[c] + O2[c] -> Glyoxylate[c] + H2O2[c] | (LOC_Os03g57220 or LOC_Os04g53210 or LOC_Os04g53214 or LOC_Os07g05820) | cytosol | 1.1.3.15 | Photorespiration |
| **ICL[c]** | Isocitrate lyase | Isocit[c] -> Glyoxylate[c] + Succ[c] | LOC_Os07g34520 | cytosol | 4.1.3.1 | Glyoxylate Cycle |
| **AK[p]** | Aspartate kinase | Asp[p] + ATP[p] -> Asp4P[p] + ADP[p] | (LOC_Os07g20544 or LOC_Os09g12290 or LOC_Os01g70300 or LOC_Os03g63330 or LOC_Os08g25390) | plastid | 2.7.2.4 | Threonine biosynthesis |
| **ASN1[p]** | Aspartate--ammonia ligase | ammonia[p] + Asp[p] + ATP[p] -> Asn[p] + ppi[p] + AMP[p] | LOC_Os03g18130 | plastid | 6.3.1.1 | Asparagine biosynthesis |
| **ASPG[c]** | Asparaginase | Asn[c] + H2O[c] -> ammonia[c] + Asp[c] + H+[c] | (LOC_Os04g58600 or LOC_Os04g46370) | cytosol | 3.5.1.1 | Asparagine biosynthesis |
| **FDH[c]** | Formate dehydrogenase | Formate[c] + NAD+[c] -> CO2[c] + NADH[c] | (LOC_Os06g29180 or LOC_Os06g29220) | cytosol | 1.2.1.2 | Folates metabolism |
| **SADN[p]** | Sulfate adenylyltransferase | sulfate[p] + ATP[p] + H+[p] -> Ade-5P[p] + ppi[p] | (LOC_Os04g02050 or LOC_Os03g53230) | plastid | 2.7.7.4 | Sulfate assimilation |
| **ARGD[c]** | Arginine deiminase | H2O[c] + Arg[c] -> ammonia[c] + citrulline[c] | LOC_Os11g44860 | cytosol | 3.5.3.6 | Proline metabolism |
| **CPA[c]** | carbamoyl-phosphate synthetase (glutamine-hydrolysing) | 2 ATP[c] + Gln[c] + HCO3[c] + H2O[c] -> Glu[c] + 2 ADP[c] + pi[c] + CarmteP[c] | (LOC_Os01g68320 or LOC_Os02g47850) | cytosol | 6.3.5.5 | Arginine biosynthesis |
| **ASN2[p]** | asparagine synthetase (glutamine-hydrolysing) | Gln[p] + Asp[p] + ATP[p] + H2O[p] -> Glu[p] + Asn[p] + ppi[p] + AMP[p] + H+[p] | LOC_Os06g15420 | plastid | 6.3.5.4 | Asparagine biosynthesis |
| **PSP[p]** | Phosphoserine phosphatase | 3PhosSer[p] + H2O[p] -> Ser[p] + pi[p] | (LOC_Os12g31820 or LOC_Os11g41160) | plastid | 3.1.3.3 | Serine biosynthesis |
| **SAT[p]** | Serine O-acetyltransferase | Ser[p] + Ace-CoA[p] -> O-Ace-L-ser[p] + CoA[p] | (LOC_Os03g08660 or LOC_Os03g10050) | plastid | 2.3.1.30 | Cysteine biosynthesis |
| **SGAT[c]** | Serine--glyoxylate aminotransferase | Glyoxylate[c] + Ser[c] -> Hydpyr[c] + Gly[c] | LOC_Os08g39300 | cytosol | 2.6.1.45 | Photorespiration |
| **MMT[c]** | Methionine S-methyltransferase | Met[c] + S-Ade-L-meth[c] -> S-Ade-L-H[c] + SMLM[c] | LOC_Os05g01470 | cytosol | 2.1.1.12 | Methionine biosynthesis |
| **HMT1[c]** | Homocysteine S-methyltransferase | HomoCys[c] + S-Ade-L-meth[c] -> S-Ade-L-H[c] + Met[c] | (LOC_Os12g41390 or LOC_Os01g56610 or LOC_Os03g12110 or LOC_Os10g28630) | cytosol | 2.1.1.10 | Methionine biosynthesis |
| **HMT2[c]** | Homocysteine S-methyltransferase | SMLM[c] + HomoCys[c] -> 2 Met[c] | (LOC_Os12g41390 or LOC_Os01g56610 or LOC_Os03g12110 or LOC_Os10g28630) | cytosol | 2.1.1.10 | Methionine biosynthesis |
| **ENO1[c]** | Phosphopyruvate hydratase | 2PG[c] <-> PEP[c] + H2O[c] | (LOC_Os03g15950 or LOC_Os03g14450 or LOC_Os06g04510 or LOC_Os10g08550) | cytosol | 4.2.1.11 | Glycolysis/Gluconeogensis |
| **ENO1[p]** | Phosphopyruvate hydratase | 2PG[p] <-> PEP[p] + H2O[p] | LOC_Os09g20820 | plastid | 4.2.1.11 | Glycolysis/Gluconeogensis |
| **CTL[c]** | Citrullinase | citrulline[c] + H2O[c] + H+[c] -> Orn[c] + CO2[c] + ammonia[c] |  | cytosol | 3.5.1.20 | Proline metabolism |
| **OAT[c]** | Ornithine--oxo-acid aminotransferase | Orn[c] + aKG[c] <-> Glu[c] + L-Glut-gsAld[c] | LOC_Os03g44150 | cytosol | 2.6.1.13 | Proline metabolism |
| **TSB2[p]** | Tryptophan synthase | Indole[p] + Ser[p] -> Trp[p] + H2O[p] | (LOC_Os03g58260 or LOC_Os07g08430) and (LOC_Os08g04180 or LOC_Os06g42560) | plastid | 4.2.1.20 | Tryptophan biosynthesis |
| **ADT[p]** | arogenate dehydratase | Arogenate[p] + H+[p] -> Phe[p] + H2O[p] + CO2[p] | (LOC_Os03g17730 or LOC_Os04g33390 or LOC_Os07g49390 or LOC_Os09g39230) | plastid | 4.2.1.91 | Phenylalanine biosynthesis |
| **LDH[c]** | L-lactate dehydrogenase | NADH[c] + Pyr[c] + H+[c] -> NAD+[c] + Lactate[c] | (LOC_Os02g01510 or LOC_Os06g01590) | cytosol | 1.1.1.27 | Fermentation |
| **MMSDH[c]** | malonate-semialdehyde dehydrogenase (acetylating) | NADP+[c] + Mal-sAld[c] + CoA[c] -> Ace-CoA[c] + NADPH[c] + CO2[c] | LOC_Os07g09060 | cytosol | 1.2.1.18 | Propanoate Metabolism |
| **IDH[m]** | Isocitrate dehydrogenase (NAD+) | Isocit[m] + NAD+[m] -> aKG[m] + CO2[m] + NADH[m] | (LOC_Os04g40320 or LOC_Os02g38200 or LOC_Os01g16900) | mitochondrion | 1.1.1.41 | TCA Cycle |
| **ALDH1[c]** | aldehyde dehydrogenase | Acetald[c] + NAD+[c] + H2O[c] -> NADH[c] + Acetate[c] + 2 H+[c] | (LOC_Os11g08300 or LOC_Os09g26880 or LOC_Os04g45720 or LOC_Os02g43280 or LOC_Os02g43194) | cytosol | 1.2.1.3 | Fermentation |
| **ALDH1[p]** | aldehyde dehydrogenase | Acetald[p] + NAD+[p] + H2O[p] -> NADH[p] + Acetate[p] + 2 H+[c] | (LOC_Os06g15990 or LOC_Os11g08300) | plastid | 1.2.1.3 | Fermentation |
| **SSADH1[m]** | Succinate-semialdehyde dehydrogenase | H2O[m] + NAD+[m] + SuccsAld[m] -> NADH[m] + Succ[m] + 2 H+[c] | LOC_Os02g07760 | mitochondrion | 1.2.1.24 | GABA metabolism |
| **TYRAAT[p]** | arogenate dehydrogenase | Arogenate[p] + NADP+[p] -> Tyr[p] + NADPH[p] + CO2[p] | (LOC_Os03g17730 or LOC_Os04g33390 or LOC_Os07g49390 or LOC_Os09g39230) | plastid | 1.3.1.78 | Tyrosine biosynthesis |
| **THRALD[p]** | L-threonine aldolase | Thr[p] -> Gly[p] + Acetald[p] | LOC_Os04g43650 | plastid | 4.1.2.5 | Glycine biosynthesis |
| **ADH[c]** | alcohol dehydrogenase | Acetald[c] + NADH[c] + H+[c] <-> Ethanol[c] + NAD+[c] | (LOC_Os02g42520 or LOC_Os02g57040 or LOC_Os03g08999 or LOC_Os03g09020 or LOC_Os07g42924 or LOC_Os10g07229 or LOC_Os11g10480 or LOC_Os11g10510 or LOC_Os11g10520) | cytosol | 1.1.1.1 | Fermentation |
| **FBP1[c]** | Fructose-bisphosphatase | Fruct16bP[c] + H2O[c] -> Fruct6P[c] + pi[c] | (LOC_Os01g64660 or LOC_Os03g18310) | cytosol | 3.1.3.11 | Glycolysis/Gluconeogensis |
| **FBP1[p]** | Fructose-bisphosphatase | Fruct16bP[p] + H2O[p] -> Fruct6P[p] + pi[p] | LOC_Os03g16050 | plastid | 3.1.3.11 | Glycolysis/Gluconeogensis |
| **FBP2[c]** | Fructose-bisphosphatase | Fruct26bP[c] + H2O[c] -> Fruct6P[c] + pi[c] | (LOC_Os01g64660 or LOC_Os03g18310) | cytosol | 3.1.3.11 | Glycolysis/Gluconeogensis |
| **FBP2[p]** | Fructose-bisphosphatase | Fruct26bP[p] + H2O[p] -> Fruct6P[p] + pi[p] | LOC_Os03g16050 | plastid | 3.1.3.11 | Glycolysis/Gluconeogensis |
| **SPS[c]** | Sucrose-phosphate synthase | UDP-Glc[c] + Fruct6P[c] -> Sucr6P[c] + UDP[c] | (LOC_Os11g12810 or LOC_Os02g09170 or LOC_Os08g20660 or LOC_Os01g69030 or LOC_Os06g43630) | cytosol | 2.4.1.14 | Sucrose metabolism |
| **PGI[c]** | Glucose-6-phosphate isomerase | b-Glc6P[c] <-> Fruct6P[c] | (LOC_Os03g56460 or LOC_Os06g14510) | cytosol | 5.3.1.9 | Sucrose metabolism |
| **PGI[p]** | Glucose-6-phosphate isomerase | b-Glc6P[p] <-> Fruct6P[p] | (LOC_Os08g37380 or LOC_Os09g29070) | plastid | 5.3.1.9 | Glycolysis/Gluconeogensis |
| **CIN[c]** | Beta-fructofuranosidase | Sucrose[c] + H2O[c] -> Fruct[c] + a-Glc[c] | (LOC_Os04g56920 or LOC_Os02g01590 or LOC_Os01g73580 or LOC_Os04g45290 or LOC_Os02g33110 or LOC_Os11g07440 or LOC_Os03g20020 or LOC_Os02g34560 or LOC_Os02g03320 or LOC_Os01g22900) | cytosol | 3.2.1.26 | Sucrose metabolism |
| **SPP[c]** | Sucrose-phosphatase | Sucr6P[c] + H2O[c] -> Sucrose[c] + pi[c] | (LOC_Os01g27880 or LOC_Os02g05030 or LOC_Os05g05270) | cytosol | 3.1.3.24 | Sucrose metabolism |
| **SUS[c]** | Sucrose synthase | UDP-Glc[c] + Fruct[c] <-> Sucrose[c] + UDP[c] | (LOC_Os03g22120 or LOC_Os02g58480 or LOC_Os04g17650 or LOC_Os04g24430) | cytosol | 2.4.1.13 | Sucrose metabolism |
| **INO1[c]** | myo-inositol-1-phosphate synthase | b-Glc6P[c] -> D-myo3mP[c] | (LOC_Os10g22450 or LOC_Os03g09250) | cytosol | 5.5.1.4 | Cell wall metabolism |
| **SIR[p]** | Sulfite reductase (ferredoxin) | sulfite[p] + 3 redferr[p] + 8 H+[p] -> sulfide[p] + 3 oxiferr[p] + 3 H2O[p] | LOC_Os05g42350 | plastid | 1.8.7.1 | Sulfate assimilation |
| **FK[c]** | Fructokinase | Fruct[c] + ATP[c] -> Fruct6P[c] + ADP[c] + H+[c] | (LOC_Os01g66940 or LOC_Os08g02120) | cytosol | 2.7.1.4 | Sucrose metabolism |
| **RCS[p]** | Cysteine synthase | O-Ace-L-ser[p] + sulfide[p] -> Cys[p] + Acetate[p] + H+[p] | (LOC_Os01g59920 or LOC_Os06g05690 or LOC_Os04g08350 or LOC_Os03g50510) | plastid | 2.5.1.47 | Cysteine biosynthesis |
| **FTHFL[c]** | Formate--tetrahydrofolate ligase | ATP[c] + Formate[c] + THF[c] -> ADP[c] + pi[c] + N10FormTHF[c] |  | cytosol | 6.3.4.3 | Folates metabolism |
| **FTHFL[m]** | Formate--tetrahydrofolate ligase | ATP[m] + Formate[m] + THF[m] -> ADP[m] + pi[m] + N10FormTHF[m] |  | mitochondrion | 6.3.4.3 | Folates metabolism |
| **FTHFL[p]** | Formate--tetrahydrofolate ligase | ATP[p] + Formate[p] + THF[p] -> ADP[p] + pi[p] + N10FormTHF[p] | LOC_Os09g27420 | plastid | 6.3.4.3 | Folates metabolism |
| **PURU[c]** | Formyltetrahydrofolate deformylase | H2O[c] + N10FormTHF[c] -> THF[c] + Formate[c] + H+[c] | LOC_Os01g49330 | cytosol | 3.5.1.10 | Folates metabolism |
| **PURU[m]** | Formyltetrahydrofolate deformylase | H2O[m] + N10FormTHF[m] -> THF[m] + Formate[m] + H+[m] | LOC_Os03g01222 | mitochondrion | 3.5.1.10 | Folates metabolism |
| **PURU[p]** | Formyltetrahydrofolate deformylase | H2O[p] + N10FormTHF[p] -> THF[p] + Formate[p] + H+[p] | (LOC_Os05g18790 or LOC_Os08g39160 or LOC_Os06g22560) | plastid | 3.5.1.10 | Folates metabolism |
| **SHM1[c]** | Glycine hydroxymethyltransferase | 510MeteTHF[c] + Gly[c] + H2O[c] <-> Ser[c] + THF[c] |  | cytosol | 2.1.2.1 | Glycine biosynthesis |
| **SHM1[m]** | Glycine hydroxymethyltransferase | 510MeteTHF[m] + Gly[m] + H2O[m] <-> Ser[m] + THF[m] | (LOC_Os01g70380 or LOC_Os01g70370) | mitochondrion | 2.1.2.1 | Glycine biosynthesis |
| **SHM1[p]** | Glycine hydroxymethyltransferase | 510MeteTHF[p] + Gly[p] + H2O[p] <-> Ser[p] + THF[p] | (LOC_Os03g52840 or LOC_Os11g26860 or LOC_Os01g65410 or LOC_Os12g22030 or LOC_Os05g35440) | plastid | 2.1.2.1 | Glycine biosynthesis |
| **APS[p]** | Glucose-1-phosphate adenylyltransferase | a-Glc1P[p] + ATP[p] + H+[p] -> ADP-D-Glu[p] + ppi[p] | (LOC_Os05g50380 or LOC_Os03g52460 or LOC_Os09g12660 or LOC_Os01g44220 or LOC_Os07g13980 or LOC_Os08g25734) | plastid | 2.7.7.27 | Starch metabolism |
| **UMPS[p]** | Orotidine-5'-phosphate decarboxylase | O5P[p] + H+[p] -> CO2[p] + UMP[p] | (LOC_Os01g72250 or LOC_Os01g72240) | plastid | 4.1.1.23 | Pyrimidine metabolism |
| **ASA[p]** | Anthranilate synthase | Chorismate[p] + Gln[p] -> Glu[p] + Anth[p] + Pyr[p] + H+[p] | ((LOC_Os03g61120 and LOC_Os06g48620) and (LOC_Os04g38950 and LOC_Os03g50880)) | plastid | 4.1.3.27 | Tryptophan biosynthesis |
| **TD[p]** | Threonine dehydratase | Thr[p] -> 2oxoBut[p] + ammonia[p] + H+[p] | LOC_Os03g50510 | plastid | 4.3.1.19 | Isoleucine biosynthesis |
| **TPI[c]** | Triosephosphate isomerase | G3P[c] <-> DHAP[c] | (LOC_Os01g05490 or LOC_Os01g62420) | cytosol | 5.3.1.1 | Glycolysis/Gluconeogensis |
| **TPI[p]** | Triosephosphate isomerase | G3P[p] <-> DHAP[p] | LOC_Os09g36450 | plastid | 5.3.1.1 | Glycolysis/Gluconeogensis; Calvin cycle |
| **PRS[p]** | ribose-phosphate diphosphokinase | ATP[p] + R5P[p] -> PRpi[p] + AMP[p] + H+[p] | LOC_Os02g03540 | plastid | 2.7.6.1 | Purine metabolism |
| **RPI[c]** | Ribose 5-phosphate epimerase | R5P[c] <-> Ru5P[c] | (LOC_Os04g24140 or LOC_Os01g36090) | cytosol | 5.3.1.6 | Pentose Phosphate Pathway |
| **RPI[p]** | Ribose 5-phosphate epimerase | R5P[p] <-> Ru5P[p] | (LOC_Os03g56869 or LOC_Os07g08030) | plastid | 5.3.1.6 | Pentose Phosphate Pathway; Calvin Cycle |
| **GAPN[c]** | Glyceraldehyde-3-phosphate dehydrogenase (NADP+) | G3P[c] + NADP+[c] + H2O[c] -> 3PG[c] + NADPH[c] + 2 H+[c] | LOC_Os03g03720 | cytosol | 1.2.1.9 | Glycolysis/Gluconeogensis |
| **GAPDH[c]** | Glyceraldehyde 3-phosphate dehydrogenase (phosphorylating) | G3P[c] + pi[c] + NAD+[c] <-> 1,3-DPGA[c] + NADH[c] + H+[c] | (LOC_Os08g03290 or LOC_Os04g40950 or LOC_Os02g38920) | cytosol | 1.2.1.12 | Glycolysis/Gluconeogensis |
| **GAPDH[p]** | Glyceraldehyde 3-phosphate dehydrogenase (phosphorylating) | G3P[p] + pi[p] + NAD+[p] <-> 1,3-DPGA[p] + NADH[p] + H+[p] | (LOC_Os04g38600 or LOC_Os02g38920) | plastid | 1.2.1.12 | Glycolysis/Gluconeogensis; Calvin cycle |
| **ALD[c]** | Fructose-bisphosphate aldolase | Fruct16bP[c] <-> DHAP[c] + G3P[c] | (LOC_Os05g33380 or LOC_Os01g02880) | cytosol | 4.1.2.13 | Glycolysis/Gluconeogensis |
| **ALD[p]** | Fructose-bisphosphate aldolase | Fruct16bP[p] <-> DHAP[p] + G3P[p] | (LOC_Os11g07020 or LOC_Os08g02700) | plastid | 4.1.2.13 | Glycolysis/Gluconeogensis |
| **ATPPRT[p]** | ATP phosphoribosyltransferase | ATP[p] + PRpi[p] -> Pr-ATP[p] + ppi[p] | LOC_Os03g04169 | plastid | 2.4.2.17 | Histidine biosynthesis |
| **AMPRT[p]** | Anthranilate phosphoribosyltransferase | Anth[p] + PRpi[p] -> N5PAnth[p] + ppi[p] | (LOC_Os06g41090 or LOC_Os02g03850 or LOC_Os04g39680 or LOC_Os03g03450 or LOC_Os05g30750) | plastid | 2.4.2.18 | Tryptophan biosynthesis |
| **FUM[m]** | Fumarate hydratase | Malate[m] <-> Fumr[m] + H2O[m] | LOC_Os03g21950 | mitochondrion | 4.2.1.2 | TCA Cycle |
| **ADNSL[p]** | Adenylosuccinate lyase | Ade-Succ[p] -> Fumr[p] + AMP[p] | (LOC_Os03g19280 or LOC_Os03g19930) | plastid | 4.3.2.2 | Purine metabolism |
| **ARGSL[c]** | Argininosuccinate lyase | ArgSucc[c] -> Arg[c] + Fumr[c] |  | cytosol | 4.3.2.1 | Arginine biosynthesis |
| **BCAT1[p]** | Branched-chain amino acid aminotransferase | Leu[p] + aKG[p] <-> Glu[p] + 2kiCapr[p] | (LOC_Os02g17330 or LOC_Os03g01600 or LOC_Os10g40200 or LOC_Os03g12890 or LOC_Os03g24460 or LOC_Os05g48450 or LOC_Os04g47190) | plastid | 2.6.1.42 | Leucine biosynthesis |
| **MTHFD1[c]** | Methylenetetrahydrofolate dehydrogenase (NAD+) | 510MeteTHF[c] + NAD+[c] -> 510MetTHF[c] + NADH[c] |  | cytosol | 1.5.1.15 | Folates metabolism |
| **MTHFD1[m]** | Methylenetetrahydrofolate dehydrogenase (NAD+) | 510MeteTHF[m] + NAD+[m] -> 510MetTHF[m] + NADH[m] |  | mitochondrion | 1.5.1.15 | Folates metabolism |
| **MTHFD1[p]** | Methylenetetrahydrofolate dehydrogenase (NAD+) | 510MeteTHF[p] + NAD+[p] -> 510MetTHF[p] + NADH[p] | LOC_Os09g15810 | plastid | 1.5.1.15 | Folates metabolism |
| **MTHFD2[c]** | Methylenetetrahydrofolate dehydrogenase (NADP+) | 510MeteTHF[c] + NADP+[c] -> NADPH[c] + 510MetTHF[c] | LOC_Os03g60090 | cytosol | 1.5.1.5 | Folates metabolism |
| **MTHFD2[m]** | Methylenetetrahydrofolate dehydrogenase (NADP+) | 510MeteTHF[m] + NADP+[m] -> NADPH[m] + 510MetTHF[m] |  | mitochondrion | 1.5.1.5 | Folates metabolism |
| **MTHFD2[p]** | Methylenetetrahydrofolate dehydrogenase (NADP+) | 510MeteTHF[p] + NADP+[p] -> NADPH[p] + 510MetTHF[p] |  | plastid | 1.5.1.5 | Folates metabolism |
| **IMPC[p]** | IMP cyclohydrolase | Pr-FormCarb[p] -> IMP[p] + H2O[p] | LOC_Os08g10570 | plastid | 3.5.4.10 | Purine metabolism |
| **PURA1[p]** | adenylosuccinate synthetase | Asp[p] + IMP[p] + GTP[p] -> Ade-Succ[p] + pi[p] + GDP[p] + 2 H+[p] | (LOC_Os03g07840 or LOC_Os03g49220) | plastid | 6.3.4.4 | Purine metabolism |
| **AMETHM[c]** | S-adenosyl-L-methionine:L-histidine N-methyltransferase | S-Ade-L-meth[c] <-> S-Ade-L-H[c] |  | cytosol | 2.1.1.- | Methionine biosynthesis |
| **HDH1[p]** | Histidinol dehydrogenase | His-al[p] + NAD+[p] + H2O[p] -> His[p] + NADH[p] + 2 H+[p] | LOC_Os01g13190 | plastid | 1.1.1.23 | Histidine biosynthesis |
| **MIOX[c]** | Myo-inositol oxygenase | O2[c] + m-Ino[c] -> Glucuronate[c] + H2O[c] + H+[c] | LOC_Os06g36560 | cytosol | 1.13.99.1 | Cell wall metabolism |
| **MIOMP[c]** | Myo-inositol-1(or 4)-monophosphatase | D-myo3mP[c] + H2O[c] -> m-Ino[c] + pi[c] | (LOC_Os02g07350 or LOC_Os03g39000) | cytosol | 3.1.3.25 | Cell wall metabolism |
| **FNR[p]** | Ferredoxin--NADP(+) reductase | 2 redferr[p] + NADP+[p] + H+[p] <-> 2 oxiferr[p] + NADPH[p] | (LOC_Os02g01340 or LOC_Os06g01850 or LOC_Os07g05400) | plastid | 1.18.1.2 | GS-GOGAT Cycle |
| **AKGDH[m]** | 2-oxoglutarate dehydrogenase | aKG[m] + CoA[m] + NAD+[m] -> Suc-CoA[m] + CO2[m] + NADH[m] | (LOC_Os07g49520 or LOC_Os04g32020) | mitochondrion | 1.2.7.3 | TCA Cycle |
| **DADH[p]** | Dihydroxy-acid dehydratase | 23dhiVal[p] -> 2kiVal[p] + H2O[p] | LOC_Os03g01030 | plastid | 4.2.1.9 | Valine biosynthesis |
| **IPMS[p]** | 2-isopropylMalate synthase | 2kiVal[p] + Ace-CoA[p] + H2O[p] -> 2iPropMal[p] + CoA[p] + H+[p] | (LOC_Os11g04670 or LOC_Os12g04440) | plastid | 2.3.3.13 | Leucine biosynthesis |
| **BCAT2[p]** | Branched-chain amino acid aminotransferase | Val[p] + aKG[p] <-> Glu[p] + 2kiVal[p] | (LOC_Os02g17330 or LOC_Os03g01600 or LOC_Os10g40200 or LOC_Os03g12890 or LOC_Os03g24460 or LOC_Os05g48450 or LOC_Os04g47190) | plastid | 2.6.1.42 | Valine biosynthesis |
| **GDC[m]** | Ser hydroxymethyl transferase | Gly[m] + THF[m] + NAD+[m] -> ammonia[m] + 510MeteTHF[m] + CO2[m] + NADH[m] + H+[m] |  | mitochondrion | 1.4.4.2, 1.8.1.4, 2.1.2.10 | Photorespiration |
| **PROC1[c]** | Pyrroline-5-carboxylate reductase | Pyrr5Carb[c] + NADH[c] + 2 H+[c] -> Pro[c] + NAD+[c] | LOC_Os01g71990 | cytosol | 1.5.1.2 | Proline metabolism |
| **PROC1[p]** | Pyrroline-5-carboxylate reductase | Pyrr5Carb[p] + NADH[p] + 2 H+[p] -> Pro[p] + NAD+[p] |  | plastid | 1.5.1.2 | Proline metabolism |
| **PROC2[c]** | Pyrroline-5-carboxylate reductase | Pyrr5Carb[c] + NADPH[c] + 2 H+[c] -> Pro[c] + NADP+[c] | LOC_Os01g71990 | cytosol | 1.5.1.2 | Proline metabolism |
| **PROC2[p]** | Pyrroline-5-carboxylate reductase | Pyrr5Carb[p] + NADPH[p] + 2 H+[p] -> Pro[p] + NADP+[p] |  | plastid | 1.5.1.2 | Proline metabolism |
| **CBL[p]** | Cystathionine beta-lyase | cysttn[p] + H2O[p] -> ammonia[p] + Pyr[p] + HomoCys[p] + H+[p] | LOC_Os06g07860 | plastid | 4.4.1.8 | Methionine biosynthesis |
| **ACO2[c]** | citrate hydrolyase | c-Aco[c] + H2O[c] <-> Isocit[c] | LOC_Os08g09200 | cytosol | 4.2.1.3 | Glyoxylate Cycle |
| **ACO2[m]** | citrate hydrolyase | c-Aco[m] + H2O[m] <-> Isocit[m] | (LOC_Os02g03260 or LOC_Os06g19960 or LOC_Os03g04410) | mitochondrion | 4.2.1.3 | TCA Cycle |
| **GADH[c]** | Glycolaldehyde dehydrogenase | H2O[c] + NAD+[c] + Glycoald[c] -> NADH[c] + Glycolate[c] + 2 H+[c] | LOC_Os07g48920 | cytosol | 1.2.1.21 | Folates metabolism |
| **PGP[p]** | Phosphoglycolate phosphatase | H2O[p] + 2PhosGlyco[p] -> Glycolate[p] + pi[p] | LOC_Os04g41340 | plastid | 3.1.3.18 | Photorespiration |
| **GPUT[c]** | Glucuronate-1-phosphate uridylyltransferase | a-D-Glucur-1-P[c] + UTP[c] + H+[c] -> UDP-Glucur[c] + ppi[c] | LOC_Os06g48760 | cytosol | 2.7.7.44 | Cell wall metabolism |
| **UDPGDC[c]** | UDP-glucuronate decarboxylase | UDP-Glucur[c] + H+[c] -> UDP-Xyl[c] + CO2[c] | (LOC_Os01g21320 or LOC_Os03g17230) | cytosol | 4.1.1.35 | Cell wall metabolism |
| **HPR[c]** | Hydroxypyruvate reductase | Hydpyr[c] + NADH[c] + H+[c] -> Glycerate[c] + NAD+[c] | LOC_Os02g01150 | cytosol | 1.1.1.29 | Photorespiration |
| **ATC[p]** | Aspartate carbamoyltransferase | Asp[p] + CarmteP[p] -> N-CarbL-Asp[p] + pi[p] + H+[p] | (LOC_Os08g15030 or LOC_Os02g47590) | plastid | 2.1.3.2 | Pyrimidine metabolism |
| **OTC[c]** | Ornithine carbamoyltransferase | Orn[c] + CarmteP[c] <-> citrulline[c] + pi[c] + H+[c] |  | cytosol | 2.1.3.3 | Proline metabolism |
| **TS[p]** | Threonine synthase | O-P-L-hmser[p] + H2O[p] -> pi[p] + Thr[p] | (LOC_Os03g50510 or LOC_Os05g47640 or LOC_Os03g11660 or LOC_Os01g49890) | plastid | 4.2.3.1 | Threonine biosynthesis |
| **UEL[c]** | UDP-arabinose 4-epimerase | UDP-Xyl[c] -> UDP-L-arab[c] | LOC_Os07g04690 | cytosol | 5.1.3.5 | Cell wall metabolism |
| **GLCAK[c]** | Glucuronokinase | Glucuronate[c] + ATP[c] -> a-D-Glucur-1-P[c] + ADP[c] + H+[c] | LOC_Os11g11060 | cytosol | 2.7.1.43 | Cell wall metabolism |
| **PGK[c]** | Phosphoglycerate kinase | 3PG[c] + ATP[c] <-> 1,3-DPGA[c] + ADP[c] | (LOC_Os01g58610 or LOC_Os02g07260 or LOC_Os05g41640 or LOC_Os06g45710 or LOC_Os10g30550) | cytosol | 2.7.2.3 | Glycolysis/Gluconeogensis |
| **PGK[p]** | Phosphoglycerate kinase | 3PG[p] + ATP[p] <-> 1,3-DPGA[p] + ADP[p] |  | plastid | 2.7.2.3 | Glycolysis/Gluconeogensis; Calvin cycle |
| **PGDH[p]** | Phosphoglycerate dehydrogenase | 3PG[p] + NAD+[p] -> 3Phoshpyr[p] + NADH[p] + H+[p] | (LOC_Os06g44460 or LOC_Os08g34720 or LOC_Os04g55720) | plastid | 1.1.1.95 | Serine biosynthesis |
| **GLYK[p]** | Glycerate kinase | Glycerate[p] + ATP[p] -> 3PG[p] + ADP[p] + H+[p] | LOC_Os01g48990 | plastid | 2.7.1.31 | Photorespiration |
| **PGLYCM[c]** | Phosphoglycerate mutase | 3PG[c] <-> 2PG[c] | (LOC_Os01g60190 or LOC_Os03g21260 or LOC_Os05g40420) | cytosol | 5.4.2.1 | Glycolysis/Gluconeogensis |
| **PGLYCM[p]** | Phosphoglycerate mutase | 3PG[p] <-> 2PG[p] | (LOC_Os11g05260 or LOC_Os08g37140) | plastid | 5.4.2.1 | Glycolysis/Gluconeogensis |
| **PRK[p]** | Phosphoribulokinase | Ru5P[p] + ATP[p] -> RuBP[p] + ADP[p] + H+[p] | (LOC_Os02g47020 or LOC_Os04g50880 or LOC_Os08g41790) | plastid | 2.7.1.19 | Calvin cycle |
| **G6PGH1[c]** | Phosphogluconate dehydrogenase (decarboxylating) | 6-P-gluco[c] + NADP+[c] -> Ru5P[c] + CO2[c] + NADPH[c] | LOC_Os06g02144 | cytosol | 1.1.1.44 | Pentose Phosphate Pathway |
| **G6PGH2[p]** | Phosphogluconate dehydrogenase (decarboxylating) | 6-P-gluco[p] + NADP+[p] -> Ru5P[p] + CO2[p] + NADPH[p] | LOC_Os11g29400 | plastid | 1.1.1.44 | Pentose Phosphate Pathway |
| **RPE[c]** | ribulose phosphate 3-epimerase | Ru5P[c] <-> Xu5P[c] | LOC_Os09g32810 | cytosol | 5.1.3.1 | Pentose Phosphate Pathway |
| **RPE[p]** | ribulose phosphate 3-epimerase | Ru5P[p] <-> Xu5P[p] | LOC_Os03g07300 | plastid | 5.1.3.1 | Pentose Phosphate Pathway; Calvin Cycle |
| **HPDH[c]** | 3-hydroxypropionate dehydrogenase | NAD+[c] + 3hprpnte[c] -> NADH[c] + Mal-sAld[c] + H+[c] |  | cytosol | 1.1.1.59 | Propanoate Metabolism |
| **TKT1[c]** | Transketolase | R5P[c] + Xu5P[c] <-> D-Sed-7P[c] + G3P[c] |  | cytosol | 2.2.1.1 | Pentose Phosphate Pathway |
| **TKT1[p]** | Transketolase | R5P[p] + Xu5P[p] <-> D-Sed-7P[p] + G3P[p] | (LOC_Os04g19740 or LOC_Os06g04270) | plastid | 2.2.1.1 | Pentose Phosphate Pathway; Calvin Cycle |
| **GABA-TK[m]** | 4-aminobutyrate aminotransferase (GABA-TK) | GABA[m] + aKG[m] -> Glu[m] + SuccsAld[m] | (LOC_Os08g10510 or LOC_Os03g44150 or LOC_Os08g41990) | mitochondrion | 2.6.1.19 | GABA metabolism |
| **GABA-TP[m]** | 4-aminobutyrate aminotransferase (GABA-TP) | GABA[m] + Pyr[m] -> Ala[m] + SuccsAld[m] | (LOC_Os08g10510 or LOC_Os03g44150 or LOC_Os08g41990) | mitochondrion | 2.6.1.19 | GABA metabolism |
| **LB1[p]** | leucine biosynthesis | 2iProp3Succ[p] -> 2kiCapr[p] + CO2[p] |  | plastid |  | Leucine biosynthesis |
| **MTHFC[m]** | Methenyltetrahydrofolate cyclohydrolase | H2O[m] + 510MetTHF[m] <-> N10FormTHF[m] + H+[m] |  | mitochondrion | 3.5.4.9 | Folates metabolism |
| **PDHE1[m]** | Pyruvate dehydrogenase (lipoamide) | Pyr[m] + pyrdeh1[m] + H+[m] -> pyrdeh2[m] + CO2[m] | ((LOC_Os09g33500 and LOC_Os08g42410) and (LOC_Os06g13720 and LOC_Os02g50620)) | mitochondrion | 1.2.4.1 | Pyruvate dehydrogenase complex |
| **PDHE1[p]** | Pyruvate dehydrogenase (lipoamide) | Pyr[p] + pyrdeh1[p] + H+[p] -> pyrdeh2[p] + CO2[p] | (LOC_Os04g02900 and (LOC_Os03g44300 and LOC_Os12g42230)) | plastid | 1.2.4.1 | Pyruvate dehydrogenase complex |
| **EMB[p]** | Chorismate synthase | 5EnoShiki3P[p] -> pi[p] + Chorismate[p] | LOC_Os03g14990 | plastid | 4.2.3.5 | Tryptophan biosynthesis |
| **CM[p]** | Chorismate mutase | Chorismate[p] -> Prep[p] | (LOC_Os08g34290 or LOC_Os02g08410 or LOC_Os12g38900 or LOC_Os01g55870) | plastid | 5.4.99.5 | Phenylalanine biosynthesis |
| **HSK[p]** | Homoserine kinase | HomoSer[p] + ATP[p] -> O-P-L-hmser[p] + ADP[p] + H+[p] | (LOC_Os04g58720 or LOC_Os02g58510) | plastid | 2.7.1.39 | Threonine biosynthesis |
| **HSDH1[p]** | Homoserine dehydrogenase | L-Asp-sAld[p] + NADH[p] + H+[p] -> HomoSer[p] + NAD+[p] | (LOC_Os09g12290 or LOC_Os08g25390) | plastid | 1.1.1.3 | Threonine biosynthesis |
| **HSDH2[p]** | Homoserine dehydrogenase | L-Asp-sAld[p] + NADPH[p] + H+[p] -> HomoSer[p] + NADP+[p] | (LOC_Os09g12290 or LOC_Os08g25390) | plastid | 1.1.1.3 | Threonine biosynthesis |
| **HXK[c]** | hexokinase | a-Glc[c] + ATP[c] -> a-Glc6P[c] + ADP[c] + H+[c] | (LOC_Os07g26540 or LOC_Os05g45590 or LOC_Os05g09500 or LOC_Os01g71320 or LOC_Os01g09460) | cytosol | 2.7.1.1 | Sucrose metabolism; Glycolysis/Gluconeogensis |
| **HXK[p]** | hexokinase | a-Glc[p] + ATP[p] -> a-Glc6P[p] + ADP[p] + H+[p] | (LOC_Os01g52450 or LOC_Os01g53930 or LOC_Os05g44760 or LOC_Os07g09890) | plastid | 2.7.1.1 | Starch metabolism; Glycolysis/Gluconeogensis |
| **PHS[p]** | starch phosphorylase | glucan[p] + pi[p] -> a-Glc1P[p] | (LOC_Os01g63270 or LOC_Os03g55090) | plastid | 2.4.1.1 | Starch metabolism |
| **DAHPS[p]** | 2-dehydro-3-deoxyphosphoheptonate aldolase | PEP[p] + D-ery4P[p] + H2O[p] -> 3DODara7P[p] + pi[p] | (LOC_Os10g41480 or LOC_Os08g37790 or LOC_Os03g27230 or LOC_Os07g42960) | plastid | 2.5.1.54 | Tryptophan biosynthesis |
| **TALA[c]** | Transaldolase | G3P[c] + D-Sed-7P[c] -> Fruct6P[c] + D-ery4P[c] |  | cytosol | 2.2.1.2 | Pentose Phosphate Pathway |
| **TALA[p]** | Transaldolase | G3P[p] + D-Sed-7P[p] -> Fruct6P[p] + D-ery4P[p] | (LOC_Os08g05830 or LOC_Os01g70170) | plastid | 2.2.1.2 | Pentose Phosphate Pathway |
| **SBPGL[p]** | Sedoheptulose 1,7-bisphosphate D-glyceraldehyde-3-phosphate-lyase | DHAP[p] + D-ery4P[p] -> D-Sed-17bP[p] |  | plastid | 4.1.2.- | Calvin cycle |
| **TKT2[c]** | Transketolase | D-ery4P[c] + Xu5P[c] <-> Fruct6P[c] + G3P[c] |  | cytosol | 2.2.1.1 | Pentose Phosphate Pathway |
| **TKT2[p]** | Transketolase | D-ery4P[p] + Xu5P[p] <-> Fruct6P[p] + G3P[p] | (LOC_Os04g19740 or LOC_Os06g04270) | plastid | 2.2.1.1 | Pentose Phosphate Pathway; Calvin Cycle |
| **SBP[p]** | Sedoheptulose-bisphosphatase | H2O[p] + D-Sed-17bP[p] -> pi[p] + D-Sed-7P[p] | LOC_Os04g16680 | plastid | 3.1.3.37 | Calvin cycle |
| **OMPD[p]** | Orotate phosphoribosyltransferase | PRpi[p] + Orot[p] -> O5P[p] + ppi[p] | (LOC_Os08g15030 or LOC_Os02g47590) | plastid | 2.4.2.10 | Pyrimidine metabolism |
| **ACO1[c]** | Aconitate hydratase | Citrate[c] <-> c-Aco[c] + H2O[c] | LOC_Os01g72240 | cytosol | 4.2.1.3 | Glyoxylate Cycle |
| **ACO1[m]** | isocitrate hydrolyase | Citrate[m] <-> c-Aco[m] + H2O[m] | (LOC_Os02g03260 or LOC_Os06g19960 or LOC_Os03g04410) | mitochondrion | 4.2.1.3 | TCA Cycle |
| **ASS[c]** | argininosuccinate synthetase | Asp[c] + citrulline[c] + ATP[c] -> ArgSucc[c] + ppi[c] + AMP[c] + H+[c] | (LOC_Os11g19770 or LOC_Os12g13320) | cytosol | 6.3.4.5 | Arginine biosynthesis |
| **DHO[p]** | Dihydroorotase | N-CarbL-Asp[p] + H+[p] -> DHooro[p] + H2O[p] | LOC_Os01g54370 | plastid | 3.5.2.3 | Pyrimidine metabolism |
| **6PGL[c]** | 6-phosphogluconolactonase | D-Glu-d-Lac6P[c] + H2O[c] -> 6-P-gluco[c] + H+[c] | (LOC_Os07g41280 or LOC_Os03g30300) | cytosol | 3.1.1.31 | Pentose Phosphate Pathway |
| **6PGL[p]** | 6-phosphogluconolactonase | D-Glu-d-Lac6P[p] + H2O[p] -> 6-P-gluco[p] + H+[p] | (LOC_Os09g35970 or LOC_Os08g43370) | plastid | 3.1.1.31 | Pentose Phosphate Pathway |
| **PFP[c]** | Diphosphate--fructose-6-phosphate 1-phosphotransferase | Fruct6P[c] + ppi[c] <-> pi[c] + Fruct16bP[c] + H+[c] | ((LOC_Os06g22060 or LOC_Os08g25720 or LOC_Os02g48360 or LOC_Os09g12650) and LOC_Os06g13810) | cytosol | 2.7.1.90 | Glycolysis/Gluconeogensis |
| **SBE[p]** | 1,4-alpha-glucan branching enzyme | glucan[p] -> Starch[p] + H2O[p] | (LOC_Os06g26234 or LOC_Os06g51084 or LOC_Os02g32660 or LOC_Os08g40930 or LOC_Os09g29404) | plastid | 2.4.1.18 | Starch metabolism |
| **CCOR[m]** | Q-cytochrome c oxidoreductase (complex III) | 2 Cyto-Oxi[m] + QH2[m] + 1.5 H+[m] -> 2 Cyto-Red[m] + Q[m] + 1.5 H+[c] | ((LOC_Os03g59220 or LOC_Os08g14860 or LOC_Os07g10500) and (LOC_Os06g07969 or LOC_Os06g07869)) | mitochondrion | 1.10.2.2 | Oxidative phosphorylation |
| **NAD9[m]** | NADH-coenzyme Q oxidoreductase (complex I) | Q[m] + NADH[m] + H+[m] -> QH2[m] + NAD+[m] | (LOC_Os02g57180 and LOC_Os03g03770 and LOC_Os03g09210 and LOC_Os03g18420 and LOC_Os03g19890 and LOC_Os03g22950 and LOC_Os03g50540 and LOC_Os03g56300 and LOC_Os04g24520 and LOC_Os05g40300 and LOC_Os05g43360 and LOC_Os05g45730 and LOC_Os06g50000 and LOC_Os07g12150 and LOC_Os07g39710 and LOC_Os07g44650 and LOC_Os08g06430 and LOC_Os08g44250 and LOC_Os10g42840 and LOC_Osm1g00210 and LOC_Osm1g00640 and LOC_Osm1g00280 and LOC_Osm1g00320 and LOC_Osm1g00460 and LOC_Osm1g00190 and LOC_Osm1g00230 and LOC_Osm1g00590) | mitochondrion | 1.6.5.3 | Oxidative phosphorylation |
| **SDH[m]** | Succinate-Q oxidoreductase (complex II) | Q[m] + Succ[m] -> QH2[m] + Fumr[m] | (LOC_Os07g04240 and LOC_Os07g33680 and LOC_Os09g20440 and LOC_Os02g02940) | mitochondrion | 1.3.5.1 | TCA Cycle |
| **BCAT3[p]** | Branched-chain amino acid aminotransferase | Ile[p] + aKG[p] <-> Glu[p] + 2k3MetVal[p] | (LOC_Os02g17330 or LOC_Os03g01600 or LOC_Os10g40200 or LOC_Os03g12890 or LOC_Os03g24460 or LOC_Os05g48450 or LOC_Os04g47190) | plastid | 2.6.1.42 | Isoleucine biosynthesis |
| **AGS[c]** | Glutamate N-acetyltransferase | Glu[c] + NAce-L-Orn[c] -> N-Ace-L-glutt[c] + Orn[c] | LOC_Os03g17120 | cytosol | 2.3.1.35 | Arginine biosynthesis |
| **AOAT[c]** | Acetylornithine aminotransferase | Glu[c] + N-A-L-gluttsemald[c] -> NAce-L-Orn[c] + aKG[c] | (LOC_Os05g03830 or LOC_Os07g27780) | cytosol | 2.6.1.11 | Arginine biosynthesis |
| **ASAD[p]** | Aspartate-semialdehyde dehydrogenase | NADPH[p] + Asp4P[p] + H+[p] -> NADP+[p] + pi[p] + L-Asp-sAld[p] | (LOC_Os03g42110 or LOC_Os10g35170 or LOC_Os03g55280) | plastid | 1.2.1.11 | Threonine biosynthesis |
| **DHDPS[p]** | dihydrodipicolinate synthase | Pyr[p] + L-Asp-sAld[p] -> 2 H2O[p] + L-23-DHDC[p] + H+[p] | (LOC_Os04g18200 or LOC_Os04g48540) | plastid | 4.3.3.7 | Lysine biosynthesis |
| **TSA[p]** | Indole-3-glycerol-phosphate lyase | ind3Ace-GP[p] -> Indole[p] + G3P[p] | LOC_Os03g58300 | plastid | 4.1.2.8 | Tryptophan biosynthesis |
| **SK[p]** | shikimate-kinase | Shikimate[p] + ATP[p] -> Shikm3P[p] + ADP[p] + H+[p] | (LOC_Os10g42700 or LOC_Os02g46220 or LOC_Os10g41580 or LOC_Os02g51410 or LOC_Os06g12150 or LOC_Os04g54800 or LOC_Os01g01302) | plastid | 2.7.1.71 | Tryptophan biosynthesis |
| **SKDH[p]** | Shikimate 5-dehydrogenase | NADPH[p] + 3DHShiki[p] + H+[p] -> NADP+[p] + Shikimate[p] | (LOC_Os01g27750 or LOC_Os12g34874) | plastid | 1.1.1.25 | Tryptophan biosynthesis |
| **SS[p]** | Starch synthase | ADP-D-Glu[p] -> ADP[p] + glucan[p] + H+[p] | (LOC_Os04g56920 or LOC_Os11g07440 or LOC_Os02g01590 or LOC_Os01g73580 or LOC_Os03g20020 or LOC_Os02g34560 or LOC_Os04g45290 or LOC_Os02g03320 or LOC_Os01g22900 or LOC_Os02g33110) | plastid | 2.4.1.21 | Starch metabolism |
| **PDHE2[m]** | Dihydrolipoamide S-acetyltransferase | CoA[m] + pyrdeh2[m] -> Ace-CoA[m] + pyrdeh3[m] | (LOC_Os06g30460 or LOC_Os06g01630 or LOC_Os02g01500 or LOC_Os07g22720) | mitochondrion | 2.3.1.12 | Pyruvate dehydrogenase complex |
| **PDHE2[p]** | Dihydrolipoamide S-acetyltransferase | CoA[p] + pyrdeh2[p] -> Ace-CoA[p] + pyrdeh3[p] | (LOC_Os01g23610 or LOC_Os05g06460) | plastid | 2.3.1.12 | Pyruvate dehydrogenase complex |
| **NAGK[c]** | Acetylglutamate kinase | N-Ace-L-glutt[c] + ATP[c] -> NAceGlutP[c] + ADP[c] | (LOC_Os04g46460 or LOC_Os03g31690 or LOC_Os07g39690 or LOC_Os02g44000) | cytosol | 2.7.2.8 | Arginine biosynthesis |
| **MFP[c]** | 3-hydroxyacyl-CoA dehydrogenase | AcrCoA[c] + H2O[c] -> 3HOPropCoA[c] | LOC_Os02g17390 | cytosol | 4.2.1.17 | Propanoate Metabolism |
| **DAPF[p]** | Diaminopimelate epimerase | LLdapime[p] -> mPime[p] | (LOC_Os01g51280 or LOC_Os03g44660 or LOC_Os07g10460) | plastid | 5.1.1.7 | Lysine biosynthesis |
| **G6PDH1[c]** | Glucose-6-phosphate 1-dehydrogenase | b-Glc6P[c] + NADP+[c] -> D-Glu-d-Lac6P[c] + NADPH[c] + H+[c] | LOC_Os12g37960 | cytosol | 1.1.1.49 | Pentose Phosphate Pathway |
| **G6PDH1[p]** | Glucose-6-phosphate 1-dehydrogenase | b-Glc6P[p] + NADP+[p] -> D-Glu-d-Lac6P[p] + NADPH[p] + H+[p] | (LOC_Os04g40874 or LOC_Os02g38840) | plastid | 1.1.1.49 | Pentose Phosphate Pathway |
| **EPI[c]** | Glucose-6 phosphate 1-epimerase | a-Glc6P[c] <-> b-Glc6P[c] | (LOC_Os01g46950 or LOC_Os04g56290 or LOC_Os08g14330) | cytosol | 5.1.3.15 | Sucrose metabolism |
| **EPI[p]** | Glucose-6 phosphate 1-epimerase | a-Glc6P[p] <-> b-Glc6P[p] |  | plastid | 5.1.3.15 | Starch metabolism |
| **CESA1[c]** | Cellulose synthase (UDP-forming) | UDP-Glc[c] -> cellulose[c] + UDP[c] + H+[c] | (LOC_Os01g56130 or LOC_Os02g09930 or LOC_Os05g43530 or LOC_Os09g25900 or LOC_Os02g51060) | cytosol | 2.4.1.12 | Cell wall metabolism |
| **HDH2[p]** | Histidinol dehydrogenase | His-ol[p] + NAD+[p] -> His-al[p] + NADH[p] + H+[p] | LOC_Os01g13190 | plastid | 1.1.1.23 | Histidine biosynthesis |
| **IMPL2[p]** | Histidinol-phosphatase | L-HisP[p] + H2O[p] -> His-ol[p] + pi[p] | LOC_Os01g13190 | plastid | 3.1.3.15 | Histidine biosynthesis |
| **KARI[p]** | Ketol-acid reductoisomerase | 2Acelac[p] + NADPH[p] + H+[p] -> 23dhiVal[p] + NADP+[p] | (LOC_Os05g49800 or LOC_Os01g46380) | plastid | 1.1.1.86 | Valine biosynthesis |
| **DHPS[m]** | Dihydropteroate synthase | p-Abenz[m] + 2a4h6hm78dP[m] -> 78dhPte[m] + ppi[m] | LOC_Os10g35250 | mitochondrion | 2.5.1.15 | Folates metabolism |
| **DHQS[p]** | 3-dehydroquinate synthase | 3DODara7P[p] -> 3DHQuin[p] + pi[p] | LOC_Os09g36800 | plastid | 4.2.3.4 | Tryptophan biosynthesis |
| **DHQDH[p]** | 3-dehydroquinate dehydratase | 3DHQuin[p] -> H2O[p] + 3DHShiki[p] | (LOC_Os12g34874 or LOC_Os01g27750) | plastid | 4.2.1.10 | Tryptophan biosynthesis |
| **RBCS-O[p]** | Ribulose-bisphosphate oxygenase | RuBP[p] + O2[p] -> 2PhosGlyco[p] + 3PG[p] + 2 H+[p] | (LOC_Os01g58020 or LOC_Os05g35330 or LOC_Os11g32770 or LOC_Os12g10580 or LOC_Os10g21280) and (LOC_Os12g19394 or LOC_Os02g05830 or LOC_Os12g17600 or LOC_Os12g19381 or LOC_Os12g19470) | plastid | 4.1.1.39 | photorespiration |
| **CHY[c]** | 3-hydroxyisobutyryl-CoA hydrolase | 3HOPropCoA[c] + pi[c] + ADP[c] -> 3hprpnte[c] + CoA[c] + ATP[c] | LOC_Os01g54860 | cytosol | 3.1.2.4 | Propanoate Metabolism |
| **HPA[p]** | Histidinol-phosphate aminotransferase | iaP[p] + Glu[p] -> L-HisP[p] + aKG[p] | (LOC_Os11g41900 or LOC_Os01g70570 or LOC_Os05g38350 or LOC_Os01g57360 or LOC_Os07g34730 or LOC_Os11g45400 or LOC_Os05g28960 or LOC_Os01g63580 or LOC_Os05g37600 or LOC_Os10g35390 or LOC_Os05g42270 or LOC_Os02g02340 or LOC_Os06g49790 or LOC_Os04g32010 or LOC_Os03g53650 or LOC_Os04g57150) | plastid | 2.6.1.9 | Histidine biosynthesis |
| **P5CS2[c]** | Glutamate-5-semialdehyde dehydrogenase | L-Glut-5P[c] + NADPH[c] + H+[c] -> L-Glut-gsAld[c] + pi[c] + NADP+[c] | (LOC_Os05g38150 or LOC_Os01g62900) | cytosol | 1.2.1.41 | Proline metabolism |
| **P5CS2[p]** | Glutamate-5-semialdehyde dehydrogenase | L-Glut-5P[p] + NADPH[p] + H+[p] -> L-Glut-gsAld[p] + pi[p] + NADP+[p] |  | plastid | 1.2.1.41 | Proline metabolism |
| **PR2[c]** | Proline biosynthesis | L-Glut-gsAld[c] <-> H2O[c] + Pyrr5Carb[c] |  | cytosol |  | Proline metabolism |
| **PR1[p]** | Proline biosynthesis | L-Glut-gsAld[p] <-> H2O[p] + Pyrr5Carb[p] |  | plastid |  | Proline metabolism |
| **AGPR[c]** | N-acetyl-gamma-glutamyl-phosphate reductase | NAceGlutP[c] + NADPH[c] + H+[c] -> N-A-L-gluttsemald[c] + NADP+[c] + pi[c] | (LOC_Os03g42110 or LOC_Os10g35170) | cytosol | 1.2.1.38 | Arginine biosynthesis |
| **IGPD[p]** | Imidazoleglycerol-phosphate dehydratase | D-eryigP[p] -> iaP[p] + H2O[p] | LOC_Os04g52710 | plastid | 4.2.1.19 | Histidine biosynthesis |
| **EPSPS[p]** | 3-phosphoshikimate 1-carboxyvinyltransferase | Shikm3P[p] + PEP[p] -> 5EnoShiki3P[p] + pi[p] | LOC_Os06g04280 | plastid | 2.5.1.19 | Tryptophan biosynthesis |
| **IGPS[p]** | indole-3-glycerol-phosphate synthase | 1-ocdoP[p] + H+[p] -> ind3Ace-GP[p] + CO2[p] + H2O[p] | LOC_Os09g08130 | plastid | 4.1.1.48 | Tryptophan biosynthesis |
| **PAI[p]** | Phosphoribosylanthranilate isomerase | N5PAnth[p] -> 1-ocdoP[p] | LOC_Os02g16630 | plastid | 5.3.1.24 | Tryptophan biosynthesis |
| **IIL[p]** | 3-isopropylMalate dehydratase | 2iPropMal[p] + H2O[p] <-> 3iPropMal[p] | (LOC_Os02g43830 or LOC_Os02g03260 or LOC_Os03g04410) | plastid | 4.2.1.33 | Leucine biosynthesis |
| **PRAPH[p]** | Phosphoribosyl-ATP pyrophosphatase | Pr-ATP[p] + H2O[p] -> Pr-AMP[p] + ppi[p] + H+[p] | LOC_Os01g16940 | plastid | 3.6.1.31 | Histidine biosynthesis |
| **PRACH[p]** | Phosphoribosyl-AMP cyclohydrolase | Pr-AMP[p] + H2O[p] -> Pr-FormCarbP[p] | LOC_Os01g16940 | plastid | 3.5.4.19 | Histidine biosynthesis |
| **PSAT[p]** | Phosphoserine aminotransferase | Glu[p] + 3Phoshpyr[p] -> 3PhosSer[p] + aKG[p] | LOC_Os03g06200 | plastid | 2.6.1.52 | Serine biosynthesis |
| **DHPR1[p]** | Dihydrodipicolinate reductase | L-23-DHDC[p] + NADH[p] + H+[p] -> Thdpico[p] + NAD+[p] | (LOC_Os02g24020 or LOC_Os03g14120) | plastid | 1.3.1.26 | Lysine biosynthesis |
| **DHPR2[p]** | Dihydrodipicolinate reductase | L-23-DHDC[p] + NADPH[p] + H+[p] -> Thdpico[p] + NADP+[p] | (LOC_Os02g24020 or LOC_Os03g14120) | plastid | 1.3.1.26 | Lysine biosynthesis |
| **IMDH[p]** | 3-isopropylMalate dehydrogenase | 3iPropMal[p] + NAD+[p] -> 2iProp3Succ[p] + NADH[p] + H+[p] | LOC_Os03g45320 | plastid | 1.1.1.85 | Leucine biosynthesis |
| **PCOR[c]** | Propanoyl-CoA: 2,3-oxidoreductase | PropCoA[c] + NAD+[c] -> AcrCoA[c] + NADH[c] | LOC_Os07g47820 | cytosol | 1.3.99.3 | Propanoate Metabolism |
| **IGPS2[p]** | Imidazole glycerol phosphate synthase | Pr-BulSylFormP[p] + Gln[p] -> Glu[p] + D-eryigP[p] + AICAR[p] + H+[p] | (LOC_Os05g33260 or LOC_Os03g15120) | plastid | 2.4.2.- | Histidine biosynthesis |
| **PRACFT[p]** | Phosphoribosyl aminoimidazolecarboxamide formyltransferase | N10FormTHF[p] + AICAR[p] -> THF[p] + Pr-FormCarb[p] | LOC_Os09g29190 | plastid | 2.1.2.3 | Purine metabolism |
| **HIS1[p]** | N-(5'-phospho-D-ribosylformimino)-5-amino-1- (5''-phosphoribosyl)-4- imidazole carboxamide isomerase | Pr-FormCarbP[p] -> Pr-BulSylFormP[p] | LOC_Os05g33260 | plastid | 5.3.1.16 | Histidine biosynthesis |
| **PFK1[c]** | 6-phosphofructokinase | ATP[c] + Fruct6P[c] -> ADP[c] + Fruct16bP[c] + H+[c] | (LOC_Os04g39420 or LOC_Os08g34050 or LOC_Os09g30240 or LOC_Os01g09570 or LOC_Os09g24910 or LOC_Os05g44922) | cytosol | 2.7.1.11 | Glycolysis/Gluconeogensis |
| **PFK2[c]** | 6-phosphofructokinase | ATP[c] + Fruct6P[c] -> ADP[c] + Fruct26bP[c] + H+[c] | (LOC_Os04g39420 or LOC_Os08g34050 or LOC_Os09g30240 or LOC_Os01g09570 or LOC_Os09g24910 or LOC_Os05g44922) | cytosol | 2.7.1.11 | Glycolysis/Gluconeogensis |
| **PFK1[p]** | 6-phosphofructokinase | ATP[p] + Fruct6P[p] -> ADP[p] + Fruct16bP[p] + H+[p] | LOC_Os10g26570 | plastid | 2.7.1.11 | Glycolysis/Gluconeogensis |
| **PFK2[p]** | 6-phosphofructokinase | ATP[p] + Fruct6P[p] -> ADP[p] + Fruct26bP[p] + H+[p] | LOC_Os10g26570 | plastid | 2.7.1.11 | Glycolysis/Gluconeogensis |
| **KARI2[p]** | Ketol-acid reductoisomerase | 2a2hButyr[p] + NADPH[p] + H+[p] -> 23dh3MetVal[p] + NADP+[p] | (LOC_Os05g49800 or LOC_Os01g46380) | plastid | 1.1.1.86 | Isoleucine biosynthesis |
| **ILVD[p]** | Dihydroxy-acid dehydratase | 23dh3MetVal[p] -> 2k3MetVal[p] + H2O[p] | LOC_Os08g44530 | plastid | 4.2.1.9 | Isoleucine biosynthesis |
| **DPE[p]** | disproportionating enzyme (D-enzyme) | glucan[p] + H2O[p] -> a-Glc[p] | (LOC_Os07g43390 or LOC_Os07g46790) | plastid | 2.4.1.25 | Starch metabolism |
| **APR1[p]** | Adenylyl-sulfate reductase (glutathione) | Ade-5P[p] + 2 Gluta[p] -> sulfite[p] + AMP[p] + GludiS[p] + 2 H+[p] | LOC_Os07g32570 | plastid | 1.8.4.9 | Sulfate assimilation |
| **FRDR[p]** | Ferredoxin--NADP(+) reductase | 2 redferr[p] + NAD+[p] + H+[p] <-> 2 oxiferr[p] + NADH[p] |  | plastid | 1.18.1.3 | GS-GOGAT Cycle |
| **AMY[p]** | alpha-amylase | Starch[p] + H2O[p] -> glucan[p] | (LOC_Os01g51754 or LOC_Os02g52700 or LOC_Os02g52710 or LOC_Os04g33040 or LOC_Os05g32710 or LOC_Os06g26234 or LOC_Os06g49970 or LOC_Os08g36900 or LOC_Os08g36910 or LOC_Os09g28400 or LOC_Os09g28420 or LOC_Os09g28430 or LOC_Os09g29404 or LOC_Os04g08270) | plastid | 3.2.1.1;3.2.1.41 | Starch metabolism |
| **MTHFR2[c]** | Methylenetetrahydrofolate reductase (NADPH) | 510MeteTHF[c] + NADH[c] + H+[c] <-> 5MetTHF[c] + NAD+[c] | LOC_Os03g60090 | cytosol | 1.5.1.20 | Folates metabolism |
| **MTHFR2[p]** | Methylenetetrahydrofolate reductase (NADPH) | 510MeteTHF[p] + NADPH[p] + H+[p] <-> 5MetTHF[p] + NADP+[p] |  | plastid | 1.5.1.20 | Folates metabolism |
| **PAT[p]** | Prephenate aminotransferase | Prep[p] + Glu[p] -> Arogenate[p] + aKG[p] |  | plastid | 2.6.1.79 | Phenylalanine biosynthesis |
| **AGD2[p]** | LL-2,6-diaminoheptanedioate aminotransferase | Thdpico[p] + Glu[p] + H2O[p] + H+[p] -> LLdapime[p] + aKG[p] | LOC_Os03g09910 | plastid | 2.6.1.83 | Lysine biosynthesis |
| **CPS[p]** | carbamoyl-phosphate synthetase (glutamine-hydrolysing) | 2 ATP[p] + Gln[p] + CO2[p] + 2 H2O[p] -> Glu[p] + 2 ADP[p] + pi[p] + CarmteP[p] + 2 H+[p] |  | plastid | 6.3.5.5 | Pyrimidine metabolism |
| **PDHE3[m]** | dihydrolipoyl dehydrogenase | pyrdeh3[m] + NAD+[m] -> pyrdeh1[m] + NADH[m] + H+[m] | (LOC_Os01g22520 or LOC_Os05g06750) | mitochondrion | 1.8.1.4 | Pyruvate dehydrogenase complex |
| **PDHE3[p]** | dihydrolipoyl dehydrogenase | pyrdeh3[p] + NAD+[p] -> pyrdeh1[p] + NADH[p] + H+[p] | LOC_Os12g08170 | plastid | 1.8.1.4 | Pyruvate dehydrogenase complex |
| **PGM[c]** | Phosphoglucomutase | a-Glc1P[c] <-> a-Glc6P[c] | LOC_Os03g50480 | cytosol | 5.4.2.2 | Sucrose metabolism |
| **PGM[p]** | Phosphoglucomutase | a-Glc1P[p] <-> a-Glc6P[p] | (LOC_Os06g28194 or LOC_Os07g26610 or LOC_Os10g11140) | plastid | 5.4.2.2 | Starch metabolism |
| **ALS2[p]** | Acetolactate synthase | Pyr[p] + 2oxoBut[p] + H+[p] -> 2a2hButyr[p] + CO2[p] | (LOC_Os06g51450 or LOC_Os02g34630 or LOC_Os11g14950 or LOC_Os02g30630 or LOC_Os04g31960 or LOC_Os02g39570 or LOC_Os03g52690 or LOC_Os03g21080 or LOC_Os04g32010) | plastid | 2.2.1.6 | Isoleucine biosynthesis |
| **ILEDG[c]** | isoleuciene degradation, lumped | CoA[c] + aKG[c] + Ile[c] -> PropCoA[c] + Ace-CoA[c] + Glu[c] |  | cytosol |  | isoleuciene degradation |
| **PSLR[p]** | Photosynthesis light reaction | 8 photon[p] + 2 H2O[p] + 3 ADP[p] + 3 pi[p] + 2 NADP+[p] -> O2[p] + 3 ATP[p] + 2 H+[p] + 2 NADPH[p] |  | plastid |  | Photosynthesis, light reaction |
| **CS[p]** | Cystathionine gamma-synthase | Cys[p] + O-P-L-hmser[p] -> cysttn[p] + pi[p] + H+[p] |  | plastid | 2.5.1.- | Methionine biosynthesis |
| **DHOX[m]** | Dihydroorotate oxidase | O2[m] + DHooro[m] -> H2O2[m] + Orot[m] |  | mitochondrion | 1.3.3.1 | Pyrimidine metabolism |
| **DHOD[c]** | Dihydroorotate dehydrogenase | NAD+[c] + DHooro[c] -> NADH[c] + Orot[c] + H+[c] | LOC_Os04g57950 | cytosol | 1.3.5.2 | Pyrimidine metabolism |
| **CA[p]** | carbonic anhydrase | CO2[p] + H2O[p] -> HCO3[p] | (LOC_Os01g45274 or LOC_Os02g33030 or LOC_Os08g32750 or LOC_Os08g32840 or LOC_Os09g28910 or LOC_Os12g05730) | plastid | 4.2.1.1 | Lipid Biosynthesis |
| **CA[c]** | carbonic anhydrase | CO2[c] + H2O[c] -> HCO3[c] | (LOC_Os04g33660 or LOC_Os08g32750 or LOC_Os08g32840 or LOC_Os08g36630 or LOC_Os11g05510 or LOC_Os12g05730) | cytosol | 4.2.1.1 | Lipid Biosynthesis |
| **ACC[p]** | Acetyl-CoA carboxylase | ATP[p] + Ace-CoA[p] + HCO3[p] -> ADP[p] + pi[p] + Mal-CoA[p] + H+[p] | LOC_Osp1g00440 | plastid | 6.4.1.2 | Lipid Biosynthesis |
| **GPD1[c]** | Glycerol-3-phosphate dehydrogenase (NAD(P)+) | DHAP[c] + NADH[c] + H+[c] <-> SGly3P[c] + NAD+[c] | LOC_Os01g74000 | cytosol | 1.1.1.8/1.1.1.94 | Lipid Biosynthesis |
| **GPD2[c]** | Glycerol-3-phosphate dehydrogenase (NAD(P)+) | DHAP[c] + NADPH[c] + H+[c] <-> SGly3P[c] + NADP+[c] |  | cytosol | 1.1.1.8/1.1.1.94 | Lipid Biosynthesis |
| **FAS160[p]** | Fatty acid synthase (n-C16:0), lumped | 21 H+[p] + 7 Mal-CoA[p] + 14 NADPH[p] + Ace-CoA[p] -> 7 CO2[p] + 7 CoA[p] + 7 H2O[p] + 14 NADP+[p] + Palmt[p] |  | plastid |  | Lipid Biosynthesis |
| **FAS180[p]** | Fatty acid synthase (n-C18:0), lumped | 24 H+[p] + 8 Mal-CoA[p] + 16 NADPH[p] + Ace-CoA[p] -> 8 CO2[p] + 8 CoA[p] + 8 H2O[p] + 16 NADP+[p] + Steat[p] |  | plastid |  | Lipid Biosynthesis |
| **FASL181[p]** | Fatty acid synthase (n-C18:1), lumped | 25 H+[p] + 8 Mal-CoA[p] + 17 NADPH[p] + Ace-CoA[p] -> 8 CO2[p] + 8 CoA[p] + 8 H2O[p] + 17 NADP+[p] + Olet[p] |  | plastid |  | Lipid Biosynthesis |
| **FASL182[p]** | Fatty acid synthase (n-C18:2), lumped | 26 H+[p] + 8 Mal-CoA[p] + 18 NADPH[p] + Ace-CoA[p] -> 8 CO2[p] + 8 CoA[p] + 8 H2O[p] + 18 NADP+[p] + Lolet[p] |  | plastid |  | Lipid Biosynthesis |
| **FAS183[p]** | Fatty acid synthase (n-C18:3), lumped | 27 H+[p] + 8 Mal-CoA[p] + 19 NADPH[p] + Ace-CoA[p] -> 8 CO2[p] + 8 CoA[p] + 8 H2O[p] + 19 NADP+[p] + Lolnt[p] |  | plastid |  | Lipid Biosynthesis |
| **FACOAL160[c]** | fatty-acid--CoA ligase (n-C16:0) | Palmt[c] + ATP[c] + CoA[c] <-> Palm-CoA[c] + AMP[c] + ppi[c] |  | cytosol |  | Lipid Biosynthesis |
| **FACOAL180[c]** | fatty-acid--CoA ligase (n-C18:0) | Steat[c] + ATP[c] + CoA[c] <-> Stea-CoA[c] + AMP[c] + ppi[c] |  | cytosol |  | Lipid Biosynthesis |
| **FACOAL181[c]** | fatty-acid--CoA ligase (n-C18:1) | Olet[c] + ATP[c] + CoA[c] <-> Ole-CoA[c] + AMP[c] + ppi[c] |  | cytosol |  | Lipid Biosynthesis |
| **FACOAL182[c]** | fatty-acid--CoA ligase (n-C18:2) | Lolet[c] + ATP[c] + CoA[c] <-> Lole-CoA[c] + AMP[c] + ppi[c] |  | cytosol |  | Lipid Biosynthesis |
| **FACOAL183[c]** | fatty-acid--CoA ligase (n-C18:3) | Lolnt[c] + ATP[c] + CoA[c] <-> Loln-CoA[c] + AMP[c] + ppi[c] |  | cytosol |  | Lipid Biosynthesis |
| **TAGS[c]** | Triglyceride synthesis | SGly3P[c] + 0.668 Palm-CoA[c] + 0.045 Stea-CoA[c] + 0.976 Ole-CoA[c] + 1.261 Lole-CoA[c] + 0.048 Loln-CoA[c] + H2O[c] -> TAG[c] + 3 CoA[c] + pi[c] |  | cytosol |  | Lipid Biosynthesis |
| **PAL[c]** | Phenylalanine ammonia-lyase | Phe[c] -> ammonia[c] + t-Cinn[c] + H+[c] | (LOC_Os02g41630 or LOC_Os04g43760) | cytosol | 4.3.1.25 | Lignin biosynthesis |
| **TAL[c]** | Tyrosine ammonia-lyase | Tyr[c] -> ammonia[c] + 4Coum[c] + H+[c] | (LOC_Os04g43760 or LOC_Os02g41630) | cytosol | 4.3.1.25 | Lignin biosynthesis |
| **CCR1[c]** | cinnamoyl-CoA reductase | 4CoumCoA[c] + NADPH[c] + H+[c] -> CoumAld[c] + NADP+[c] + CoA[c] | (LOC_Os09g31518 or LOC_Os09g31502 or LOC_Os09g31490 or LOC_Os03g60380 or LOC_Os03g60279) | cytosol | 1.2.1.44 | Lignin biosynthesis |
| **4CL[c]** | 4-coumarate--CoA ligase | CoA[c] + 4Coum[c] + ATP[c] -> 4CoumCoA[c] + ppi[c] + AMP[c] | (LOC_Os08g34790 or LOC_Os08g04770 or LOC_Os06g44620 or LOC_Os03g05780 or LOC_Os02g08100 or LOC_Os08g14760 or LOC_Os02g46970) | cytosol | 6.2.1.12 | Lignin biosynthesis |
| **CAMT[c]** | Caffeoyl-CoA O-methyltransferase | Caff-CoA[c] + S-Ade-L-meth[c] -> S-Ade-L-H[c] + Fer-CoA[c] | (LOC_Os09g30360 or LOC_Os06g06980 or LOC_Os08g38900) | cytosol | 2.1.1.104 | Lignin biosynthesis |
| **CCQT[c]** | Caffeoyl-CoA: quinate O-(3,4-dihydroxycinnamoyl)transferase | CaffQuin[c] + CoA[c] -> Caff-CoA[c] + Quinate[c] | (LOC_Os02g39850 or LOC_Os04g42251) | cytosol | 3.1.2.- | Lignin biosynthesis |
| **FCR[c]** | feruloyl-CoA reductase | Fer-CoA[c] + NADPH[c] + H+[c] -> Conald[c] + NADP+[c] + CoA[c] | (LOC_Os09g31518 or LOC_Os09g31502 or LOC_Os09g31490 or LOC_Os03g60380 or LOC_Os03g60279) | cytosol | 1.2.1.44 | Lignin biosynthesis |
| **C4H[c]** | Trans-cinnamate 4-monooxygenase | t-Cinn[c] + O2[c] + NADPH[c] + H+[c] -> H2O[c] + NADP+[c] + 4Coum[c] | (LOC_Os01g60450 or LOC_Os05g25640) | cytosol | 1.14.13.11 | Lignin biosynthesis |
| **HCT[c]** | Shikimate O-hydroxycinnamoyltransferase | 4CoumCoA[c] + Shikimate[c] -> 4CoumShiki[c] + CoA[c] | LOC_Os04g42250 | cytosol | 2.3.1.133 | Lignin biosynthesis |
| **CAD1[c]** | cinnamyl-alcohol dehydrogenase | Conald[c] + NADPH[c] + H+[c] -> Conalc[c] + NADP+[c] | LOC_Os02g09490 | cytosol | 1.1.1.195 | Lignin biosynthesis |
| **CAD2[c]** | cinnamyl-alcohol dehydrogenase | SinaAld[c] + NADPH[c] + H+[c] -> SinapAlc[c] + NADP+[c] | LOC_Os02g09490 | cytosol | 1.1.1.195 | Lignin biosynthesis |
| **CQMOX[c]** | coumaroylquinate 3'-monooxygenase | 4CoumQuin[c] + NADPH[c] + O2[c] + H+[c] -> CaffQuin[c] + NADP+[c] + H2O[c] | LOC_Os05g41440 | cytosol | 1.14.13.36 | Lignin biosynthesis |
| **FH[c]** | ferulate 5-hydroxylase | Conald[c] + NADPH[c] + O2[c] + H+[c] -> 5hConif[c] + NADP+[c] + H2O[c] | (LOC_Os03g02180 or LOC_Os10g36848) | cytosol | 1.14.13.- | Lignin biosynthesis |
| **AMETH[c]** | S-adenosyl-L-methionine: 3,4-dihydroxy-trans-cinnamate 3-O-methyltransferase | 5hConif[c] + S-Ade-L-meth[c] -> S-Ade-L-H[c] + SinaAld[c] + H+[c] | (LOC_Os08g06100 or LOC_Os02g57760 or LOC_Os04g01470 or LOC_Os12g13800) | cytosol | 2.1.1.68 | Lignin biosynthesis |
| **CSMOX[c]** | coumaroylshikimate 3'-monooxygenase | 4CoumShiki[c] + NADPH[c] + O2[c] + H+[c] -> CaffShiki[c] + NADP+[c] + H2O[c] | LOC_Os05g41440 | cytosol | 1.14.13.36 | Lignin biosynthesis |
| **CCQH[c]** | p-coumaroyl-CoA:quinate hydroxycinnamoyltransferase | 4CoumCoA[c] + Quinate[c] -> 4CoumQuin[c] + CoA[c] | LOC_Os04g42250 | cytosol | 2.3.1.133 | Lignin biosynthesis |
| **HCCST[c]** | Hydroxycinnamoyl-CoA:shikimate hydroxycinnamoyltransferase | CaffShiki[c] + CoA[c] -> Caff-CoA[c] + Shikimate[c] | (LOC_Os02g39850 or LOC_Os04g42251) | cytosol | 3.1.2.- | Lignin biosynthesis |
| **CAD3[c]** | cinnamyl-alcohol dehydrogenase | CoumAld[c] + NADPH[c] + H+[c] -> Coum-Alc[c] + NADP+[c] | LOC_Os02g09490 | cytosol | 1.1.1.195 | Lignin biosynthesis |
| **TCM01** | THF transporter | THF[m] -> THF[c] |  | transport |  | transport |
| **TCM02** | Formate transporter | Formate[c] <-> Formate[m] |  | transport |  | transport |
| **TCM03** | AMP transporter | AMP[m] + ATP[c] + 2 H+[c] -> AMP[c] + ATP[m] + 2 H+[m] |  | transport |  | transport |
| **TCM04** | Pyruvate transporter | Pyr[c] + H+[c] -> Pyr[m] + H+[m] |  | transport |  | transport |
| **TCM05** | Aspartate transporter | Asp[m] + H+[m] <-> Asp[c] + H+[c] |  | transport |  | transport |
| **TCM06** | Dihydroorotate transporter | Dhooro[c] -> Dhooro[m] |  | transport |  | transport |
| **TCM07** | Orotate transporter | Orot[m] -> Orot[c] |  | transport |  | transport |
| **TCM08** | Alanine transporter | Ala[m] + H+[m] <-> Ala[c] + H+[c] |  | transport |  | transport |
| **TCM09** | Water transporter | H2O[c] <-> H2O[m] |  | transport |  | transport |
| **TCM10** | Carbon dioxide transporter | CO2[c] <-> CO2[m] |  | transport |  | transport |
| **TCM11** | Ammonia transporter | ammonia[c] <-> ammonia[m] |  | transport |  | transport |
| **TCM12** | Oxygen transporter | O2[c] <-> O2[m] |  | transport |  | transport |
| **TCM13** | Proton/Phosphate transporter | pi[c] + H+[c] <-> pi[m] + H+[m] |  | transport |  | transport |
| **TCM14** | Malate/Oxaloacetate transporter | Malate[m] + OAA[c] <-> Malate[c] + OAA[m] |  | transport |  | transport |
| **TCM15** | Hydrogen peroxide transporter | H2O2[c] <-> H2O2[m] |  | transport |  | transport |
| **TCM16** | ATP/ADP transporter | ATP[m] + ADP[c] + H+[c] -> ATP[c] + ADP[m] + H+[m] |  | transport |  | transport |
| **TCM17** | GABA/proton transporter | GABA[c] + H+[m] <-> GABA[m] + H+[c] |  | transport |  | transport |
| **TCM18** | Succinate/Fumarate transporter | Succ[m] + Fumr[c] <-> Fumr[m] + Succ[c] |  | transport |  | transport |
| **TCM19** | Glycine transporter | Gly[m] + H+[m] <-> Gly[c] + H+[c] |  | transport |  | transport |
| **TCM20** | Serine transporter | Ser[m] + H+[m] <-> Ser[c] + H+[c] |  | transport |  | transport |
| **TCM21** | Succinate/Malate transporter | Succ[c] + Malate[m] <-> Succ[m] + Malate[c] |  | transport |  | transport |
| **TCM22** | Citrate/Malate transporter | Citrate[m] + Malate[c] <-> Citrate[c] + Malate[m] |  | transport |  | transport |
| **TCM23** | Malate/phosphate transporter | Malate[c] + pi[m] <-> pi[c] + Malate[m] |  | transport |  | transport |
| **TCM24** | Malate/alpha-Ketoglutarate transporter | Malate[c] + aKG[m] -> Malate[m] + aKG[c] |  | transport |  | transport |
| **TCM25** | CoA transporter | CoA[c] -> CoA[m] |  | transport |  | transport |
| **TCM26** | Acetate transporter | Acetate[c] -> Acetate[m] |  | transport |  | transport |
| **TCM27** | Aspartate/Glutamate transporter | Asp[m] + Glu[c] -> Asp[c] + Glu[m] |  | transport |  | transport |
| **TCP01** | Aspartate transporter | Asp[p] + H+[p] <-> Asp[c] + H+[c] |  | transport |  | transport |
| **TCP02** | Cysteine transporter | Cys[p] + H+[p] <-> Cys[c] + H+[c] |  | transport |  | transport |
| **TCP03** | Glycine transporter | Gly[p] + H+[p] <-> Gly[c] + H+[c] |  | transport |  | transport |
| **TCP04** | Histidine transporter | His[p] + H+[p] <-> His[c] + H+[c] |  | transport |  | transport |
| **TCP05** | Isoleucine transporter | Ile[p] + H+[p] <-> Ile[c] + H+[c] |  | transport |  | transport |
| **TCP06** | Leucine transporter | Leu[p] + H+[p] <-> Leu[c] + H+[c] |  | transport |  | transport |
| **TCP07** | Lysine transporter | Lys[p] + H+[p] <-> Lys[c] + H+[c] |  | transport |  | transport |
| **TCP08** | Methionine transporter | Met[p] + H+[p] <-> Met[c] + H+[c] |  | transport |  | transport |
| **TCP09** | Phenylalanine transporter | Phe[p] + H+[p] <-> Phe[c] + H+[c] |  | transport |  | transport |
| **TCP10** | Proline transporter | Pro[p] + H+[p] <-> Pro[c] + H+[c] |  | transport |  | transport |
| **TCP11** | Serine transporter | Ser[p] + H+[p] <-> Ser[c] + H+[c] |  | transport |  | transport |
| **TCP12** | Threonine transporter | Thr[p] + H+[p] <-> Thr[c] + H+[c] |  | transport |  | transport |
| **TCP13** | Tryptophan transporter | Trp[p] + H+[p] <-> Trp[c] + H+[c] |  | transport |  | transport |
| **TCP14** | Tyrosine transporter | Tyr[p] + H+[p] <-> Tyr[c] + H+[c] |  | transport |  | transport |
| **TCP15** | Valine transporter | Val[p] + H+[p] <-> Val[c] + H+[c] |  | transport |  | transport |
| **TCP16** | p-aminobenzoate transporter | p-Abenz[p] -> p-Abenz[c] |  | transport |  | transport |
| **TCP17** | Tetrahydrofolate transporter | THF[c] -> THF[p] |  | transport |  | transport |
| **TCP18** | Formate transporter | Formate[c] <-> Formate[p] |  | transport |  | transport |
| **TCP19** | Glucose transporter | a-Glc[p] -> a-Glc[c] |  | transport |  | transport |
| **TCP20** | Hexose Phosphate/Phosphate translocator (GPT) | a-Glc1P[c] + pi[p] <-> a-Glc1P[p] + pi[c] |  | transport |  | transport |
| **TCP21** | Hexose Phosphate/Phosphate translocator (GPT) | a-Glc6P[c] + pi[p] <-> a-Glc6P[p] + pi[c] |  | transport |  | transport |
| **TCP22** | Triose phosphate-3-phosphoglycerate-phosphate translocator (TPT) | DHAP[c] + pi[p] <-> DHAP[p] + pi[c] |  | transport |  | transport |
| **TCP23** | Triose phosphate-3-phosphoglycerate-phosphate translocator (TPT) | 3PG[p] + pi[c] <-> 3PG[c] + pi[p] |  | transport |  | transport |
| **TCP24** | Phosphoenolpyruvate/Phosphate translocator (PPT) | PEP[c] + pi[p] -> PEP[p] + pi[c] |  | transport |  | transport |
| **TCP25** | AMP transporter | AMP[p] + ATP[c] + 2 H+[c] -> AMP[c] + ATP[p] + 2 H+[p] |  | transport |  | transport |
| **TCP26** | Dihydroorotate transporter | DHooro[p] -> DHooro[c] |  | transport |  | transport |
| **TCP27** | Orotate transporter | Orot[c] -> Orot[p] |  | transport |  | transport |
| **TCP28** | UTP transporter | UTP[p] <-> UTP[c] |  | transport |  | transport |
| **TCP29** | Water transporter | H2O[c] <-> H2O[p] |  | transport |  | transport |
| **TCP30** | Carbon dioxide transporter | CO2[c] <-> CO2[p] |  | transport |  | transport |
| **TCP31** | Glutamate/Glutamine transporter | Gln[p] + Glu[c] -> Glu[p] + Gln[c] |  | transport |  | transport |
| **TCP32** | Ammonia transporter | ammonia[c] <-> ammonia[p] |  | transport |  | transport |
| **TCP33** | Oxygen transporter | O2[c] <-> O2[p] |  | transport |  | transport |
| **TCP34** | Phosphate transporter | pi[c] + H+[c] <-> pi[p] + H+[p] |  | transport |  | transport |
| **TCP35** | Sulfate transporter | sulfate[c] -> sulfate[p] |  | transport |  | transport |
| **TCP36** | Homocysteine transporter | HomoCys[p] -> HomoCys[c] |  | transport |  | transport |
| **TCP37** | ATP/ADP transporter | ATP[p] + ADP[c] + H+[c] <-> ATP[c] + ADP[p] + H+[p] |  | transport |  | transport |
| **TCP38** | Glycolate/Glycerate transporter | 2 Glycolate[p] + Glycerate[c] <-> 2 Glycolate[c] + Glycerate[p] |  | transport |  | transport |
| **TCP39** | Malate/Fumarate transporter | Malate[p] + Fumr[c] <-> Fumr[p] + Malate[c] |  | transport |  | transport |
| **TCP40** | Oxaloacetate/Malate transporter | Malate[p] + OAA[c] -> OAA[p] + Malate[c] |  | transport |  | transport |
| **TCP41** | alpha-Ketoglutarate/Malate transporter | Malate[p] + aKG[c] -> aKG[p] + Malate[c] |  | transport |  | transport |
| **TCP42** | Glutamate/Malate transporter | Malate[c] + Glu[p] -> Glu[c] + Malate[p] |  | transport |  | transport |
| **TCP43** | FA transporter | Palmt[p] -> Palmt[c] |  | transport |  | transport |
| **TCP44** | FA transporter | Steat[p] -> Steat[c] |  | transport |  | transport |
| **TCP45** | FA transporter | Olet[p] -> Olet[c] |  | transport |  | transport |
| **TCP46** | FA transporter | Lolet[p] -> Lolet[c] |  | transport |  | transport |
| **TCP47** | FA transporter | Lolnt[p] -> Lolnt[c] |  | transport |  | transport |
| **TCP48** | CoA transporter | CoA[c] -> CoA[p] |  | transport |  | transport |

**Metabolites List**

| **Abbreviation** | **Metabolite** | **KEGG ID** | **Compartment** |
| --- | --- | --- | --- |
| **1,3-DPGA[c]** | 1,3-diphosphoglycerate | C00236 | Cytosol |
| **1,3-DPGA[p]** | 1,3-diphosphoglycerate | C00236 | Plastid |
| **1-ocdoP[p]** | 1-(o-carboxyphenylamino)-1'-deoxyribulose-5'-phosphate | C01302 | Plastid |
| **23dh3MetVal[p]** | 2,3-dihydroxy-3-methylvalerate | C06007 | Plastid |
| **23dhiVal[p]** | 2,3-dihydroxy-isovalerate | C04272 | Plastid |
| **2a2hButyr[p]** | 2-aceto-2-hydroxy-butyrate | C06006 | Plastid |
| **2a4h6hm78dP[m]** | 2-amino-4-hydroxy-6-hydroxymethyl-7,8-dihydropteridine diphosphate | C04807 | Mitochondrion |
| **2Acelac[p]** | 2-acetolactate | C06010 | Plastid |
| **2iProp3Succ[p]** | 2-isopropyl-3-oxosuccinate | C04236 | Plastid |
| **2iPropMal[p]** | 2-isopropylmalate | C02504 | Plastid |
| **2k3MetVal[p]** | 2-keto-3-methyl-valerate | C00671 | Plastid |
| **2kiCapr[p]** | 2-ketoisocaproate | C00233 | Plastid |
| **2kiVal[p]** | 2-keto-isovalerate | C00141 | Plastid |
| **2oxoBut[p]** | 2-oxobutanoate | C00109 | Plastid |
| **2PG[c]** | 2-phosphoglycerate | C00631 | Cytosol |
| **2PG[p]** | 2-phosphoglycerate | C00631 | Plastid |
| **2PhosGlyco[p]** | 2-phosphoglycolate | C00988 | Plastid |
| **3DHQuin[p]** | 3-dehydroquinate | C00944 | Plastid |
| **3DHShiki[p]** | 3-dehydro-shikimate | C02637 | Plastid |
| **3DODara7P[p]** | 3-deoxy-D-arabino-heptulosonate-7-phosphate | C04691 | Plastid |
| **3HOPropCoA[c]** | 3-hydroxypropionyl-CoA | C05668 | Cytosol |
| **3hprpnte[c]** | 3-Hydroxypropanoate | C01013 | Cytosol |
| **3iPropMal[p]** | 3-isopropylmalate | C04411 | Plastid |
| **3PG[c]** | 3-phosphoglycerate | C00197 | Cytosol |
| **3PG[p]** | 3-phosphoglycerate | C00197 | Plastid |
| **3Phoshpyr[p]** | 3-phospho-hydroxypyruvate | C03232 | Plastid |
| **3PhosSer[p]** | 3-phospho-serine | C01005 | Plastid |
| **4Coum[c]** | 4-coumarate | C00811 | Cytosol |
| **4CoumCoA[c]** | 4-coumaroyl-CoA | C00223 | Cytosol |
| **4CoumQuin[c]** | 4-coumaroylquinate | C12208 | Cytosol |
| **4CoumShiki[c]** | 4-coumaroylshikimate | C02947 | Cytosol |
| **510MeteTHF[c]** | 5,10-methylene-THF | C00143 | Cytosol |
| **510MeteTHF[m]** | 5,10-methylene-THF | C00143 | Mitochondrion |
| **510MeteTHF[p]** | 5,10-methylene-THF | C00143 | Plastid |
| **510MetTHF[c]** | 5,10-methenyl-THF | C00445 | Cytosol |
| **510MetTHF[m]** | 5,10-methenyl-THF | C00445 | Mitochondrion |
| **510MetTHF[p]** | 5,10-methenyl-THF | C00445 | Plastid |
| **5EnoShiki3P[p]** | 5-enolpyruvyl-shikimate-3-phosphate | C01269 | Plastid |
| **5hConif[c]** | 5-hydroxy-coniferaldehyde | C12204 | Cytosol |
| **5MetTHF[c]** | 5-methyl-THF | C00440 | Cytosol |
| **5MetTHF[p]** | 5-methyl-THF | C00440 | Plastid |
| **6-P-gluco[c]** | 6-phospho-D-gluconate | C00345 | Cytosol |
| **6-P-gluco[p]** | 6-phospho-D-gluconate | C00345 | Plastid |
| **78dhPte[m]** | 7,8-dihydropteroate | C00921 | Mitochondrion |
| **Ace-CoA[c]** | acetyl-CoA | C00024 | Cytosol |
| **Ace-CoA[m]** | acetyl-CoA | C00024 | Mitochondrion |
| **Ace-CoA[p]** | acetyl-CoA | C00024 | Plastid |
| **Acetald[c]** | acetaldehyde | C00084 | Cytosol |
| **Acetald[p]** | acetaldehyde | C00084 | Plastid |
| **Acetate[c]** | acetate | C00033 | Cytosol |
| **Acetate[m]** | acetate | C00033 | Mitochondrion |
| **Acetate[p]** | acetate | C00033 | Plastid |
| **AcrCoA[c]** | Acrylyl-CoA | C00894 | Cytosol |
| **Ade-5P[p]** | adenosine 5'-phosphosulfate | C00224 | Plastid |
| **Adenosine[c]** | adenosine | C00212 | Cytosol |
| **Ade-Succ[p]** | adenylo-succinate | C03794 | Plastid |
| **a-D-Glucur-1-P[c]** | alpha-D-glucuronate 1-phosphate | C05385 | Cytosol |
| **ADP[c]** | ADP | C00008 | Cytosol |
| **ADP[m]** | ADP | C00008 | Mitochondrion |
| **ADP[p]** | ADP | C00008 | Plastid |
| **ADP-D-Glu[p]** | ADP-D-glucose | C00498 | Plastid |
| **a-Glc[c]** | alpha-D-glucose | C00267 | Cytosol |
| **a-Glc[e]** | alpha-D-glucose | C00267 | Extracellular |
| **a-Glc[p]** | alpha-D-glucose | C00267 | Plastid |
| **a-Glc1P[c]** | alpha-D-glucose 1-phosphate | C00103 | Cytosol |
| **a-Glc1P[p]** | alpha-D-glucose 1-phosphate | C00103 | Plastid |
| **a-Glc6P[c]** | alpha-D-glucose 6-phosphate | C00668 | Cytosol |
| **a-Glc6P[p]** | alpha-D-glucose 6-phosphate | C00668 | Plastid |
| **AICAR[p]** | AICAR | C04677 | Plastid |
| **aKG[c]** | alpha-ketoglutarate | C00026 | Cytosol |
| **aKG[m]** | alpha-ketoglutarate | C00026 | Mitochondrion |
| **aKG[p]** | alpha-ketoglutarate | C00026 | Plastid |
| **Ala[c]** | L-alanine | C00041 | Cytosol |
| **Ala[e]** | L-alanine | C00041 | Extracellular |
| **Ala[m]** | L-alanine | C00041 | Mitochondrion |
| **ammonia[c]** | ammonia | C00014 | Cytosol |
| **ammonia[m]** | ammonia | C00014 | Mitochondrion |
| **ammonia[p]** | ammonia | C00014 | Plastid |
| **AMP[c]** | AMP | C00020 | Cytosol |
| **AMP[m]** | AMP | C00020 | Mitochondrion |
| **AMP[p]** | AMP | C00020 | Plastid |
| **Anth[p]** | anthranilate | C00108 | Plastid |
| **Arg[c]** | L-arginine | C00062 | Cytosol |
| **ArgSucc[c]** | L-arginino-succinate | C03406 | Cytosol |
| **Arogenate[p]** | Arogenate | C00826 | Plastid |
| **Asn[c]** | L-asparagine | C00152 | Cytosol |
| **Asn[e]** | L-asparagine | C00152 | Extracellular |
| **Asn[p]** | L-asparagine | C00152 | Plastid |
| **Asp[c]** | L-aspartate | C00049 | Cytosol |
| **Asp[m]** | L-aspartate | C00049 | Mitochondrion |
| **Asp[p]** | L-aspartate | C00049 | Plastid |
| **Asp4P[p]** | L-aspartyl-4-phosphate | C03082 | Plastid |
| **ATP[c]** | ATP | C00002 | Cytosol |
| **ATP[m]** | ATP | C00002 | Mitochondrion |
| **ATP[p]** | ATP | C00002 | Plastid |
| **b-Glc6P[c]** | beta-D-glucose-6-phosphate | C01172 | Cytosol |
| **b-Glc6P[p]** | beta-D-glucose-6-phosphate | C01172 | Plastid |
| **c-Aco[c]** | cis-aconitate | C00417 | Cytosol |
| **c-Aco[m]** | cis-aconitate | C00417 | Mitochondrion |
| **Caff-CoA[c]** | caffeoyl-CoA | C00323 | Cytosol |
| **CaffQuin[c]** | caffeoylquinate | C00852 | Cytosol |
| **CaffShiki[c]** | caffeoylshikimate | C10434 | Cytosol |
| **CarmteP[c]** | carbamoyl-phosphate | C00169 | Cytosol |
| **CarmteP[p]** | carbamoyl-phosphate | C00169 | Plastid |
| **cellulose[c]** | cellulose | C00760 | Cytosol |
| **Chorismate[p]** | chorismate | C00251 | Plastid |
| **Citrate[c]** | citrate | C00158 | Cytosol |
| **Citrate[m]** | citrate | C00158 | Mitochondrion |
| **citrulline[c]** | citrulline | C00327 | Cytosol |
| **CO2[c]** | CO2 | C00011 | Cytosol |
| **CO2[e]** | CO2 | C00011 | Extracellular |
| **CO2[m]** | CO2 | C00011 | Mitochondrion |
| **CO2[p]** | CO2 | C00011 | Plastid |
| **CoA[c]** | coenzyme A | C00010 | Cytosol |
| **CoA[m]** | coenzyme A | C00010 | Mitochondrion |
| **CoA[p]** | coenzyme A | C00010 | Plastid |
| **Conalc[c]** | coniferyl alcohol | C00590 | Cytosol |
| **Conald[c]** | coniferyl aldehyde | C02666 | Cytosol |
| **Coum-Alc[c]** | coumaryl-alcohol | C02646 | Cytosol |
| **CoumAld[c]** | coumaraldehyde | C05608 | Cytosol |
| **Cys[c]** | L-cysteine | C00097 | Cytosol |
| **Cys[p]** | L-cysteine | C00097 | Plastid |
| **cysttn[p]** | cystathionine | C02291 | Plastid |
| **Cyto-Oxi[m]** | Cytochromes-C-Oxidized | none | Mitochondrion |
| **Cyto-Red[m]** | Cytochromes-C-Reduced | none | Mitochondrion |
| **D-ery4P[c]** | D-erythrose-4-phosphate | C00279 | Cytosol |
| **D-ery4P[p]** | D-erythrose-4-phosphate | C00279 | Plastid |
| **D-eryigP[p]** | D-erythro-imidazole-glycerol-phosphate | C04666 | Plastid |
| **D-Glu-d-Lac6P[c]** | D-glucono-&delta;-lactone-6-phosphate | C01236 | Cytosol |
| **D-Glu-d-Lac6P[p]** | D-glucono-&delta;-lactone-6-phosphate | C01236 | Plastid |
| **DHAP[c]** | dihydroxy-acetone-phosphate | C00111 | Cytosol |
| **DHAP[p]** | dihydroxy-acetone-phosphate | C00111 | Plastid |
| **Dhooro[c]** | dihydroorotate | C00337 | Cytosol |
| **DHooro[c]** | dihydroorotate | C00337 | Cytosol |
| **Dhooro[m]** | dihydroorotate | C00337 | Mitochondrion |
| **DHooro[m]** | dihydroorotate | C00337 | Mitochondrion |
| **DHooro[p]** | dihydroorotate | C00337 | Plastid |
| **D-myo3mP[c]** | D-myo-inositol (3)-monophosphate | C04006 | Cytosol |
| **D-Sed-17bP[p]** | D-sedoheptulose-1,7-bisphosphate | C00447 | Plastid |
| **D-Sed-7P[c]** | D-sedoheptulose-7-phosphate | C00281 | Cytosol |
| **D-Sed-7P[p]** | D-sedoheptulose-7-phosphate | C00281 | Plastid |
| **Ethanol[c]** | ethanol | C00469 | Cytosol |
| **Ethanol[e]** | ethanol | C00469 | Extracellular |
| **Fer-CoA[c]** | feruloyl-CoA | C00406 | Cytosol |
| **Formate[c]** | formate | C00058 | Cytosol |
| **Formate[m]** | formate | C00058 | Mitochondrion |
| **Formate[p]** | formate | C00058 | Plastid |
| **Fruct[c]** | fructose | C00095 | Cytosol |
| **Fruct16bP[c]** | fructose-1,6-bisphosphate | C00354 | Cytosol |
| **Fruct16bP[p]** | fructose-1,6-bisphosphate | C00354 | Plastid |
| **Fruct26bP[c]** | fructose-2,6-bisphosphate | C00665 | Cytosol |
| **Fruct26bP[p]** | fructose-2,6-bisphosphate | C00665 | Plastid |
| **Fruct6P[c]** | fructose-6-phosphate | C00085 | Cytosol |
| **Fruct6P[p]** | fructose-6-phosphate | C00085 | Plastid |
| **Fumr[c]** | fumarate | C00122 | Cytosol |
| **Fumr[m]** | fumarate | C00122 | Mitochondrion |
| **Fumr[p]** | fumarate | C00122 | Plastid |
| **G3P[c]** | D-glyceraldehyde-3-phosphate | C00661 | Cytosol |
| **G3P[p]** | D-glyceraldehyde-3-phosphate | C00661 | Plastid |
| **GABA[c]** | 4-aminobutyrate | C00334 | Cytosol |
| **GABA[m]** | 4-aminobutyrate | C00334 | Mitochondrion |
| **GDP[c]** | GDP | C00035 | Cytosol |
| **GDP[p]** | GDP | C00035 | Plastid |
| **Gln[c]** | L-glutamine | C00064 | Cytosol |
| **Gln[e]** | L-glutamine | C00064 | Extracellular |
| **Gln[p]** | L-glutamine | C00064 | Plastid |
| **Glu[c]** | L-glutamate | C00025 | Cytosol |
| **Glu[m]** | L-glutamate | C00025 | Mitochondrion |
| **Glu[p]** | L-glutamate | C00025 | Plastid |
| **glucan[p]** | Large-branched-glucans | none | Plastid |
| **Glucuronate[c]** | glucuronate | C00191 | Cytosol |
| **GludiS[p]** | glutathione disulfide | C00127 | Plastid |
| **Gluta[p]** | glutathione | C00051 | Plastid |
| **Gly[c]** | glycine | C00037 | Cytosol |
| **Gly[m]** | glycine | C00037 | Mitochondrion |
| **Gly[p]** | glycine | C00037 | Plastid |
| **Glycerate[c]** | glycerate | C00258 | Cytosol |
| **Glycerate[p]** | glycerate | C00258 | Plastid |
| **Glycoald[c]** | glycolaldehyde | C00266 | Cytosol |
| **Glycolate[c]** | glycolate | C00160 | Cytosol |
| **Glycolate[p]** | glycolate | C00160 | Plastid |
| **Glyoxylate[c]** | glyoxylate | C00048 | Cytosol |
| **Glyoxylate[p]** | glyoxylate | C00048 | Plastid |
| **GTP[c]** | GTP | C00044 | Cytosol |
| **GTP[p]** | GTP | C00044 | Plastid |
| **H+[c]** | H+ | C00080 | Cytosol |
| **H+[e]** | H+ | C00080 | Extracellular |
| **H+[m]** | H+ | C00080 | Mitochondrion |
| **H+[p]** | H+ | C00080 | Plastid |
| **H2O[c]** | H2O | C00001 | Cytosol |
| **H2O[e]** | H2O | C00001 | Extracellular |
| **H2O[m]** | H2O | C00001 | Mitochondrion |
| **H2O[p]** | H2O | C00001 | Plastid |
| **H2O2[c]** | H2O2 | C00027 | Cytosol |
| **H2O2[m]** | H2O2 | C00027 | Mitochondrion |
| **H2O2[p]** | H2O2 | C00027 | Plastid |
| **HCO3[c]** | Bicarbonate | C00288 | Cytosol |
| **HCO3[p]** | Bicarbonate | C00288 | Plastid |
| **His[c]** | L-histidine | C00388 | Cytosol |
| **His[p]** | L-histidine | C00388 | Plastid |
| **His-al[p]** | histidinal | C01929 | Plastid |
| **His-ol[p]** | histidinol | C00860 | Plastid |
| **HomoCys[c]** | L-homocysteine | C00155 | Cytosol |
| **HomoCys[p]** | L-homocysteine | C00155 | Plastid |
| **HomoSer[p]** | homoserine | C00263 | Plastid |
| **Hydpyr[c]** | hydroxypyruvate | C00168 | Cytosol |
| **iaP[p]** | imidazole acetol-phosphate | C01267 | Plastid |
| **Ile[c]** | L-isoleucine | C00407 | Cytosol |
| **Ile[p]** | L-isoleucine | C00407 | Plastid |
| **IMP[p]** | inosine-5'-phosphate | C00130 | Plastid |
| **ind3Ace-GP[p]** | indole-3-glycerol-phosphate | C03506 | Plastid |
| **Indole[p]** | indole | C00463 | Plastid |
| **Isocit[c]** | isocitrate | C00451 | Cytosol |
| **Isocit[m]** | isocitrate | C00451 | Mitochondrion |
| **L-23-DHDC[p]** | L-2,3-dihydrodipicolinate | C03340 | Plastid |
| **Lactate[c]** | L-lactate | C00186 | Cytosol |
| **Lactate[e]** | L-lactate | C00186 | Extracellular |
| **L-Asp-sAld[p]** | L-aspartate-semialdehyde | C00441 | Plastid |
| **Leu[c]** | L-leucine | C00123 | Cytosol |
| **Leu[p]** | L-leucine | C00123 | Plastid |
| **L-Glut-5P[c]** | L-glutamate-5-phosphate | C03287 | Cytosol |
| **L-Glut-5P[p]** | L-glutamate-5-phosphate | C03287 | Plastid |
| **L-Glut-gsAld[c]** | L-glutamate-gamma-semialdehyde | C01165 | Cytosol |
| **L-Glut-gsAld[p]** | L-glutamate-gamma-semialdehyde | C01165 | Plastid |
| **L-HisP[p]** | L-histidinol-phosphate | C01100 | Plastid |
| **LLdapime[p]** | L,L-diaminopimelate | C00666 | Plastid |
| **Lole-CoA[c]** | Linoleic CoA | C02050 | Cytosol |
| **Lolet[c]** | Linoleic acid | C01595 | Cytosol |
| **Lolet[p]** | Linoleic acid | C01595 | Plastid |
| **Loln-CoA[c]** | Linolenic CoA | C16162 | Cytosol |
| **Lolnt[c]** | Linolenic acid | C06427 | Cytosol |
| **Lolnt[p]** | Linolenic acid | C06427 | Plastid |
| **Lys[c]** | L-lysine | C00047 | Cytosol |
| **Lys[p]** | L-lysine | C00047 | Plastid |
| **Malate[c]** | malate | C00149 | Cytosol |
| **Malate[m]** | malate | C00149 | Mitochondrion |
| **Malate[p]** | malate | C00149 | Plastid |
| **Mal-CoA[p]** | Malonyl-CoA | C00083 | Plastid |
| **Mal-sAld[c]** | Malonate semialdehyde | C00222 | Cytosol |
| **Met[c]** | L-methionine | C00073 | Cytosol |
| **Met[p]** | L-methionine | C00073 | Plastid |
| **m-Ino[c]** | myo-inositol | C00137 | Cytosol |
| **mPime[p]** | meso-diaminopimelate | C00680 | Plastid |
| **N10FormTHF[c]** | N10-formyl-THF | C00234 | Cytosol |
| **N10FormTHF[m]** | N10-formyl-THF | C00234 | Mitochondrion |
| **N10FormTHF[p]** | N10-formyl-THF | C00234 | Plastid |
| **N5PAnth[p]** | N-(5'-phosphoribosyl)-anthranilate | C04302 | Plastid |
| **NAceGlutP[c]** | N-acetylglutamyl-phosphate | C04133 | Cytosol |
| **N-Ace-L-glutt[c]** | N-acetyl-L-glutamate | C00624 | Cytosol |
| **NAce-L-Orn[c]** | N-acetyl-L-ornithine | C00437 | Cytosol |
| **NAD+[c]** | NAD+ | C00003 | Cytosol |
| **NAD+[m]** | NAD+ | C00003 | Mitochondrion |
| **NAD+[p]** | NAD+ | C00003 | Plastid |
| **NADH[c]** | NADH | C00004 | Cytosol |
| **NADH[m]** | NADH | C00004 | Mitochondrion |
| **NADH[p]** | NADH | C00004 | Plastid |
| **NADP+[c]** | NADP+ | C00006 | Cytosol |
| **NADP+[m]** | NADP+ | C00006 | Mitochondrion |
| **NADP+[p]** | NADP+ | C00006 | Plastid |
| **NADPH[c]** | NADPH | C00005 | Cytosol |
| **NADPH[m]** | NADPH | C00005 | Mitochondrion |
| **NADPH[p]** | NADPH | C00005 | Plastid |
| **N-A-L-gluttsemald[c]** | N-acetyl-L-glutamate 5-semialdehyde | C01250 | Cytosol |
| **N-CarbL-Asp[p]** | N-carbamoyl-L-aspartate | C00438 | Plastid |
| **O2[c]** | O2 | C00007 | Cytosol |
| **O2[e]** | O2 | C00007 | Extracellular |
| **O2[m]** | O2 | C00007 | Mitochondrion |
| **O2[p]** | O2 | C00007 | Plastid |
| **O5P[p]** | orotidine-5'-phosphate | C01103 | Plastid |
| **OAA[c]** | oxaloacetate | C00036 | Cytosol |
| **OAA[m]** | oxaloacetate | C00036 | Mitochondrion |
| **OAA[p]** | oxaloacetate | C00036 | Plastid |
| **O-Ace-L-ser[p]** | O-acetyl-L-serine | C00979 | Plastid |
| **Ole-CoA[c]** | Oleic CoA | C00510 | Cytosol |
| **Olet[c]** | Oleic acid | C00712 | Cytosol |
| **Olet[p]** | Oleic acid | C00712 | Plastid |
| **O-P-L-hmser[p]** | O-phospho-L-homoserine | C01102 | Plastid |
| **Orn[c]** | L-ornithine | C00077 | Cytosol |
| **Orot[c]** | orotate | C00295 | Cytosol |
| **Orot[m]** | orotate | C00295 | Mitochondrion |
| **Orot[p]** | orotate | C00295 | Plastid |
| **oxiferr[p]** | Oxidized-ferredoxins | none | Plastid |
| **p-Abenz[c]** | p-aminobenzoate | C00568 | Cytosol |
| **p-Abenz[m]** | p-aminobenzoate | C00568 | Mitochondrion |
| **p-Abenz[p]** | p-aminobenzoate | C00568 | Plastid |
| **Palm-CoA[c]** | Palmityl-CoA | C00154 | Cytosol |
| **Palmt[c]** | Palmitic acid | C00249 | Cytosol |
| **Palmt[p]** | Palmitic acid | C00249 | Plastid |
| **PEP[c]** | phosphoenolpyruvate | C00074 | Cytosol |
| **PEP[p]** | phosphoenolpyruvate | C00074 | Plastid |
| **Phe[c]** | L-phenylalanine | C00079 | Cytosol |
| **Phe[p]** | L-phenylalanine | C00079 | Plastid |
| **photon[e]** | photon | none | Extracellular |
| **photon[p]** | photon | none | Plastid |
| **pi[c]** | Orthophosphate | C00009 | Cytosol |
| **pi[e]** | Orthophosphate | C00009 | Extracellular |
| **pi[m]** | Orthophosphate | C00009 | Mitochondrion |
| **pi[p]** | Orthophosphate | C00009 | Plastid |
| **ppi[c]** | Pyrophosphate | C00013 | Cytosol |
| **ppi[m]** | Pyrophosphate | C00013 | Mitochondrion |
| **ppi[p]** | Pyrophosphate | C00013 | Plastid |
| **Pr-AMP[p]** | phosphoribosyl-AMP | C02741 | Plastid |
| **Pr-ATP[p]** | phosphoribosyl-ATP | C02739 | Plastid |
| **Pr-BulSylFormP[p]** | phosphoribulosylformimino-AICAR-P | C04916 | Plastid |
| **Prep[p]** | prephenate | C00254 | Plastid |
| **Pr-FormCarb[p]** | phosphoribosyl-formamido-carboxamide | C04734 | Plastid |
| **Pr-FormCarbP[p]** | phosphoribosylformiminoAICAR-phosphate | C04896 | Plastid |
| **Pro[c]** | L-proline | C00148 | Cytosol |
| **Pro[p]** | L-proline | C00148 | Plastid |
| **PropCoA[c]** | Propanoyl-CoA | C00100 | Cytosol |
| **PRpi[p]** | 5-Phospho-alpha-D-ribose | C00119 | Plastid |
| **Pyr[c]** | pyruvate | C00022 | Cytosol |
| **Pyr[m]** | pyruvate | C00022 | Mitochondrion |
| **Pyr[p]** | pyruvate | C00022 | Plastid |
| **pyrdeh1[m]** | Pyruvate-dehydrogenase-lipoate | C15972 | Mitochondrion |
| **pyrdeh1[p]** | Pyruvate-dehydrogenase-lipoate | C15972 | Plastid |
| **pyrdeh2[m]** | Pyruvate-dehydrogenase-acetylDHlipoyl | C16255 | Mitochondrion |
| **pyrdeh2[p]** | Pyruvate-dehydrogenase-acetylDHlipoyl | C16255 | Plastid |
| **pyrdeh3[m]** | Pyruvate-dehydrogenase-dihydrolipoate | C15973 | Mitochondrion |
| **pyrdeh3[p]** | Pyruvate-dehydrogenase-dihydrolipoate | C15973 | Plastid |
| **Pyrr5Carb[c]** | pyrroline5-carboxylate | C03912 | Cytosol |
| **Pyrr5Carb[p]** | pyrroline5-carboxylate | C03912 | Plastid |
| **Q[m]** | Ubiquinones | C00399 | Mitochondrion |
| **QH2[m]** | Ubiquinols | C00390 | Mitochondrion |
| **Quinate[c]** | quinate | C00296 | Cytosol |
| **R5P[c]** | D-ribose-5-phosphate | C00117 | Cytosol |
| **R5P[p]** | D-ribose-5-phosphate | C00117 | Plastid |
| **redferr[p]** | Reduced-ferredoxins | none | Plastid |
| **Ru5P[c]** | D-ribulose-5-phosphate | C00199 | Cytosol |
| **Ru5P[p]** | D-ribulose-5-phosphate | C00199 | Plastid |
| **RuBP[p]** | D-ribulose-1,5-bisphosphate | C01182 | Plastid |
| **S-Ade-L-H[c]** | S-adenosyl-L-homocysteine | C00021 | Cytosol |
| **S-Ade-L-meth[c]** | S-adenosyl-L-methionine | C00019 | Cytosol |
| **Ser[c]** | L-serine | C00065 | Cytosol |
| **Ser[m]** | L-serine | C00065 | Mitochondrion |
| **Ser[p]** | L-serine | C00065 | Plastid |
| **SGly3P[c]** | Glycerol-3-phosphate | C00093 | Cytosol |
| **Shikimate[c]** | shikimate | C00493 | Cytosol |
| **Shikimate[p]** | shikimate | C00493 | Plastid |
| **Shikm3P[p]** | shikimate-3-phosphate | C03175 | Plastid |
| **SinaAld[c]** | sinapaldehyde | C05610 | Cytosol |
| **SinapAlc[c]** | sinapyl-alcohol | C02325 | Cytosol |
| **SMLM[c]** | S-methyl-L-methionine | C03172 | Cytosol |
| **Starch[e]** | Starch | C00369 | Extracellular |
| **Starch[p]** | Starch | C00369 | Plastid |
| **Stea-CoA[c]** | stearoyl-CoA | C00412 | Cytosol |
| **Steat[c]** | Stearic acid | C01530 | Cytosol |
| **Steat[p]** | Stearic acid | C01530 | Plastid |
| **Succ[c]** | succinate | C00042 | Cytosol |
| **Succ[e]** | succinate | C00042 | Extracellular |
| **Succ[m]** | succinate | C00042 | Mitochondrion |
| **Suc-CoA[m]** | succinyl-CoA | C00091 | Mitochondrion |
| **SuccsAld[m]** | succinate semialdehyde | C00232 | Mitochondrion |
| **Sucr6P[c]** | sucrose-6-phosphate | C02591 | Cytosol |
| **Sucrose[c]** | sucrose | C00089 | Cytosol |
| **Sucrose[e]** | sucrose | C00089 | Extracellular |
| **sulfate[c]** | sulfate | C00059 | Cytosol |
| **sulfate[e]** | sulfate | C00059 | Extracellular |
| **sulfate[p]** | sulfate | C00059 | Plastid |
| **sulfide[p]** | hydrogen sulfide | C00283 | Plastid |
| **sulfite[p]** | sulfite | C00094 | Plastid |
| **TAG[c]** | Triacylglycerides | none | Cytosol |
| **t-Cinn[c]** | trans-cinnamate | C00423 | Cytosol |
| **Thdpico[p]** | tetrahydrodipicolinate | C03972 | Plastid |
| **THF[c]** | tetrahydrofolate | C00101 | Cytosol |
| **THF[m]** | tetrahydrofolate | C00101 | Mitochondrion |
| **THF[p]** | tetrahydrofolate | C00101 | Plastid |
| **Thr[c]** | L-threonine | C00188 | Cytosol |
| **Thr[p]** | L-threonine | C00188 | Plastid |
| **Trp[c]** | L-tryptophan | C00078 | Cytosol |
| **Trp[p]** | L-tryptophan | C00078 | Plastid |
| **Tyr[c]** | L-tyrosine | C00082 | Cytosol |
| **Tyr[p]** | L-tyrosine | C00082 | Plastid |
| **UDP[c]** | UDP | C00015 | Cytosol |
| **UDP[p]** | UDP | C00015 | Plastid |
| **UDP-Gal[c]** | UDP-galactose | C00052 | Cytosol |
| **UDP-Glc[c]** | UDP-D-glucose | C00029 | Cytosol |
| **UDP-Glucur[c]** | UDP-D-glucuronate | C00167 | Cytosol |
| **UDP-L-arab[c]** | UDP-L-arabinose | C00935 | Cytosol |
| **UDP-Xyl[c]** | UDP-D-xylose | C00190 | Cytosol |
| **UMP[p]** | UMP | C00105 | Plastid |
| **UTP[c]** | UTP | C00075 | Cytosol |
| **UTP[p]** | UTP | C00075 | Plastid |
| **Val[c]** | L-valine | C00183 | Cytosol |
| **Val[p]** | L-valine | C00183 | Plastid |
| **Xu5P[c]** | D-xylulose-5-phosphate | C00231 | Cytosol |
| **Xu5P[p]** | D-xylulose-5-phosphate | C00231 | Plastid |
