# Supplementary material for: Metabolic and transcriptional regulatory mechanisms underlying the anoxic adaptation of rice coleoptile
Source: AoB Plants. 2014 Jun 3;6:plu026. doi: 10.1093/aobpla/plu026 (PMC4077593; doi:10.1093/aobpla/plu026)
Supplement: Additional Information [file supp_plu026_plu026supp_data2.doc]

**Supplemental File S2**

# Combined in silico metabolic flux sampling and microarray data analysis reveals key transcriptional mechanisms in anoxic adaptation of rice coleoptile

Meiyappan Lakshmanan, Bijayalaxmi Mohanty, Sun-Hyung Lim, Sun-Hwa H3 and Dong-Yup Lee

Department of Chemical and Biomolecular Engineering, National University of Singapore, Singapore.

**Sampling histograms of various central metabolic reactions in rice model**

In all figures, the red and blue lines indicate the distribution of anaerobic and aerobic fluxes, respectively. The x-axis represents attainable flux values and y-axis represents probability distribution

**Cytosolic Glycolysis**

**
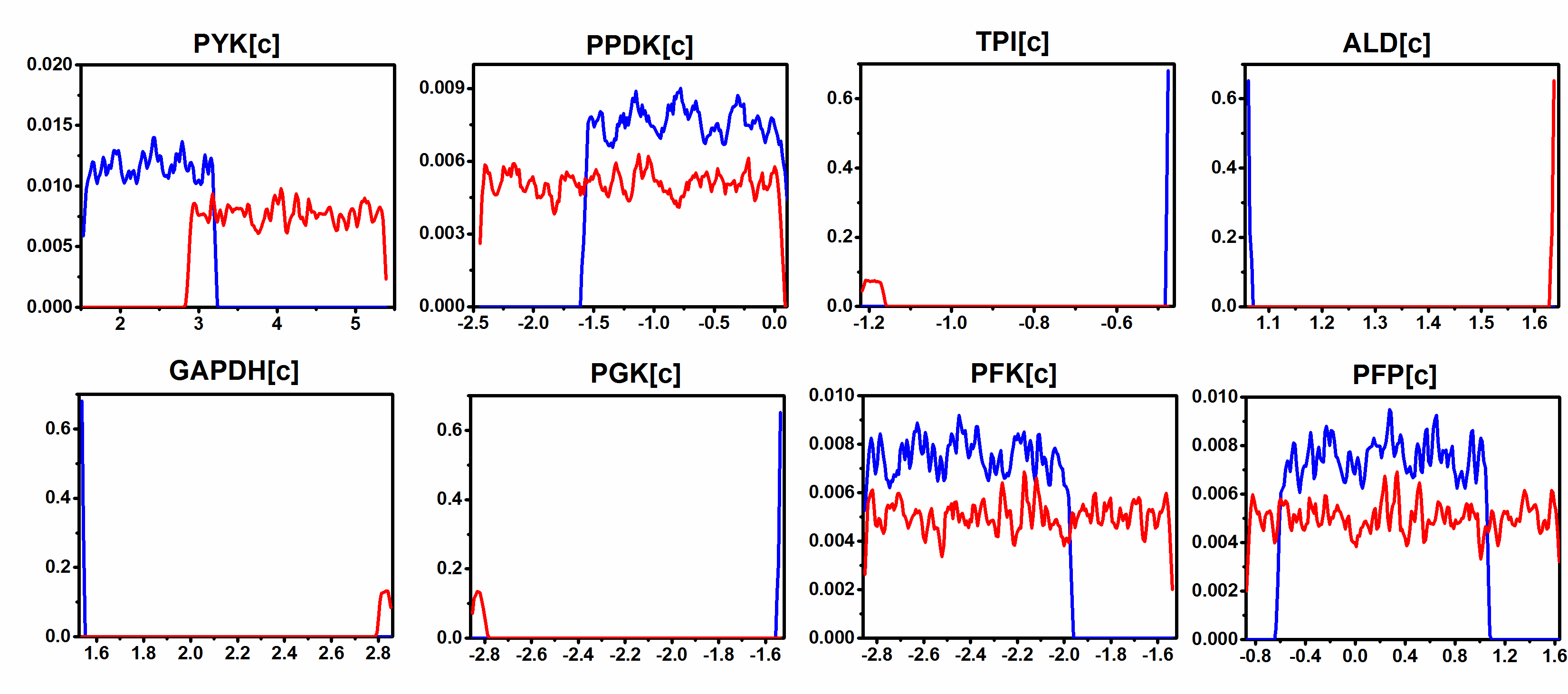
**

**Plastidic Glycolysis**

**
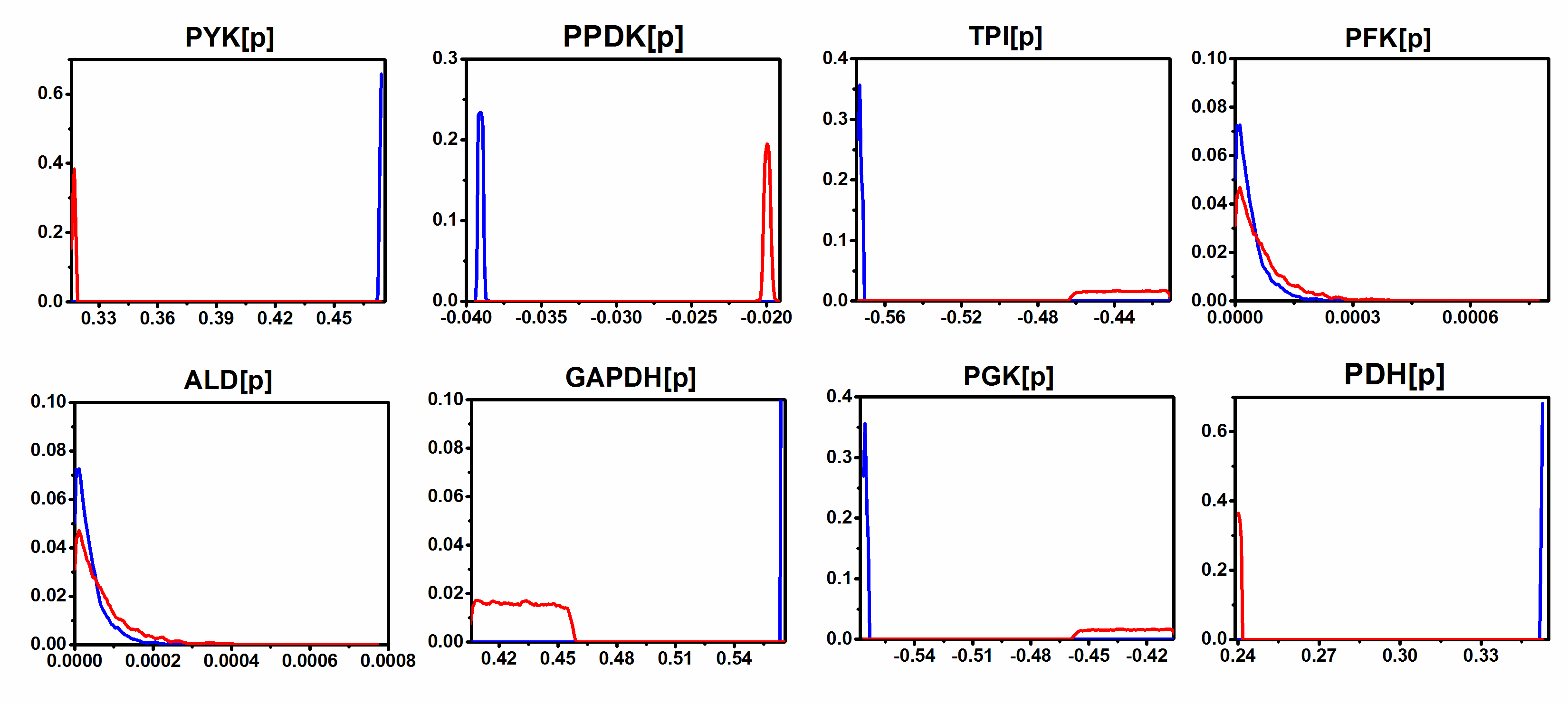
**

**Oxidative Phosphorylation**

**
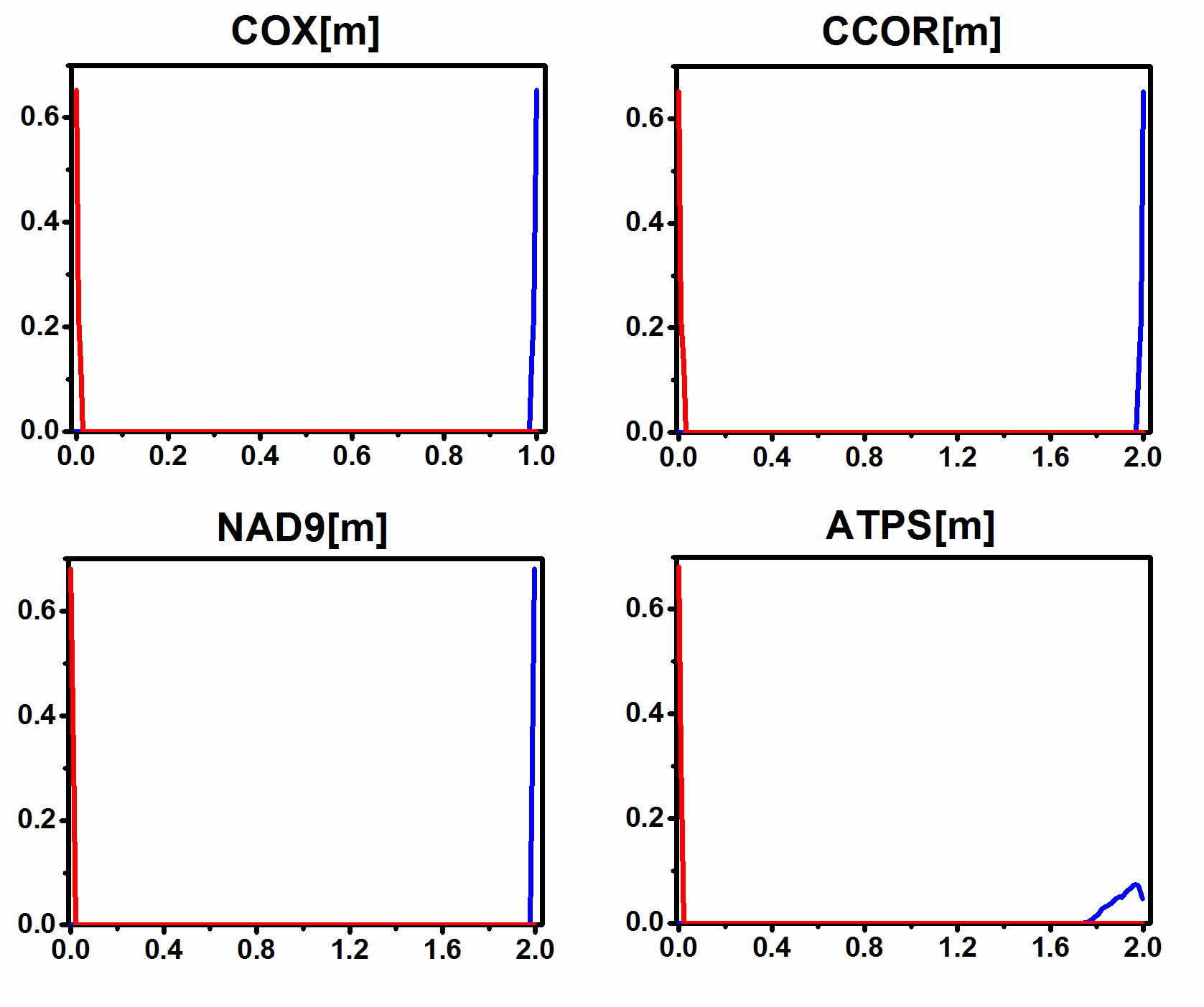
**

**Fermentation**

**
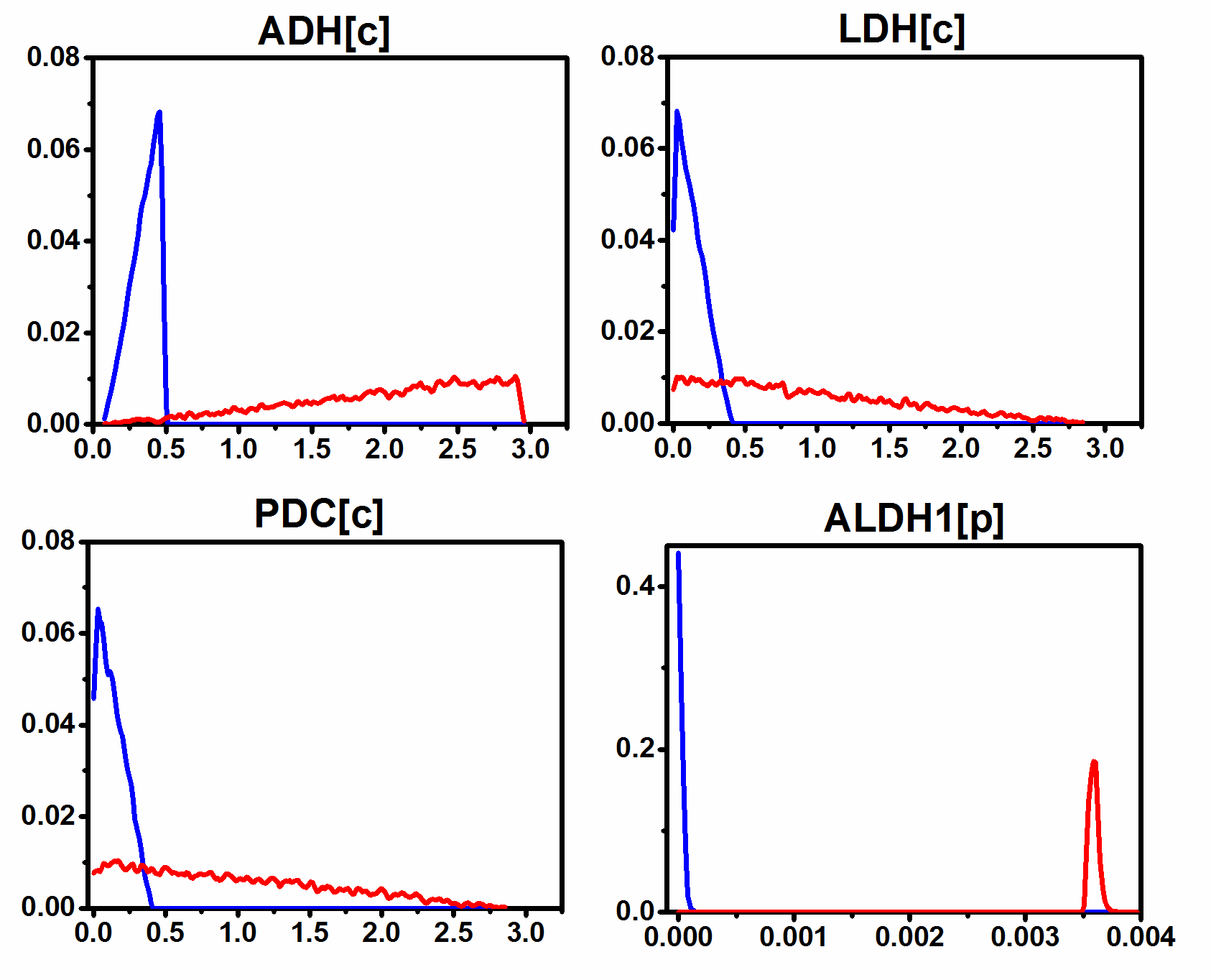
**

**TCA Cycle**

**
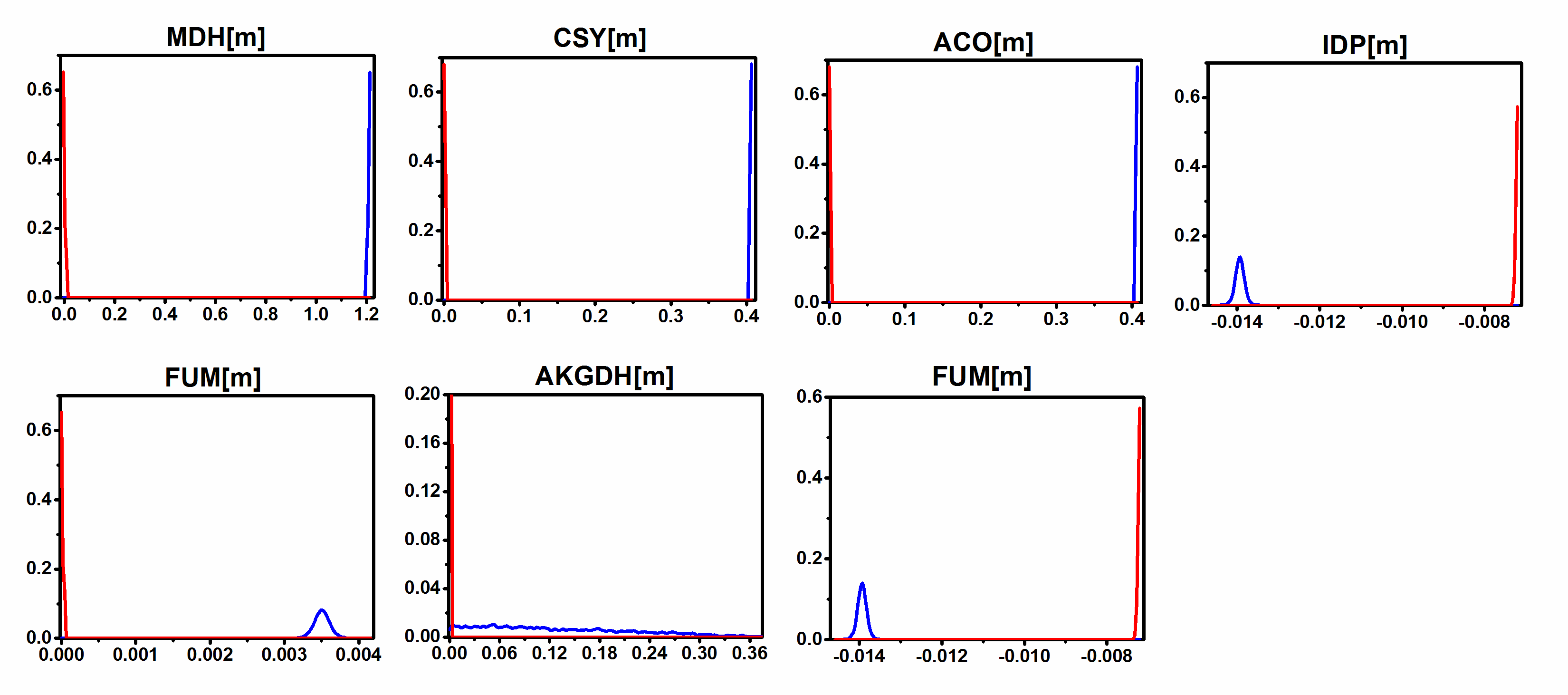
**
