# Supplementary material for: Metabolic and transcriptional regulatory mechanisms underlying the anoxic adaptation of rice coleoptile
Source: AoB Plants. 2014 Jun 3;6:plu026. doi: 10.1093/aobpla/plu026 (PMC4077593; doi:10.1093/aobpla/plu026)
Supplement: Additional Information [file supp_plu026_plu026supp_data3.doc]

**Supplemental File S3**

# Combined in silico metabolic flux sampling and microarray data analysis reveals key transcriptional mechanisms in anoxic adaptation of rice coleoptile

Meiyappan Lakshmanan, Bijayalaxmi Mohanty, Sun-Hyung Lim, Sun-Hwa H3 and Dong-Yup Lee

Department of Chemical and Biomolecular Engineering, National University of Singapore, Singapore.

**List of rice central metabolic reactions and their flux differences, p-values, expression patterns of corresponding gene locuses and type of regulation**

| **Reaction** | **Reaction name** | **Subsystem** | **Mean flux difference (aerobic-anaerobic)** | **Z-score** | **p-value** | **Corresponding down-regulated genes** | **Corresponding up-regulated** | **Down-regulated/Up-regulated** | **How regulated?** |
| --- | --- | --- | --- | --- | --- | --- | --- | --- | --- |
| **MDH[c]** | Malate dehydrogenase | Glycolysis/Gluconeogenesis | 1.497E+00 | 9.965E+03 | 0.000 | LOC_Os10g33800 |  | 1/0 | Down-regulated |
| **ALD[c]** | Fructose-bisphosphate aldolase | Glycolysis/Gluconeogenesis | -2.983E-01 | 2.053E+04 | 0.000 |  | LOC_Os06g40640 | 0/1 | Up-regulated |
| **PGK[c]** | Phosphoglycerate kinase | Glycolysis/Gluconeogenesis | -8.902E-01 | 8.347E+03 | 0.000 |  | LOC_Os02g07260; LOC_Os06g45710 | 0/2 | Up-regulated |
| **GAPDH[c]** | Glyceraldehyde 3-phosphate dehydrogenase (phosphorylating) | Glycolysis/Gluconeogenesis | -8.902E-01 | 8.347E+03 | 0.000 |  | LOC_Os02g38920 | 0/1 | Up-regulated |
| **TPI[c]** | Triosephosphate isomerase | Glycolysis/Gluconeogenesis | -5.919E-01 | 4.563E+03 | 0.000 |  | LOC_Os01g62420 | 0/1 | Up-regulated |
| **PYK[c]** | Pyruvate kinase | Glycolysis/Gluconeogenesis | -1.179E+00 | 1.991E+02 | 0.000 |  | LOC_Os01g16960 | 0/1 | Up-regulated |
| **PPC[c]** | Phosphoenolpyruvate carboxylase | Glycolysis/Gluconeogenesis | 8.120E-03 | 2.829E+01 | 0.000 | LOC_Os09g14670; LOC_Os08g27840; LOC_Os02g14770 | LOC_Os01g02050 | 3/1 | Down-regulated |
| **PCKA[c]** | Phosphoenolpyruvate carboxykinase (ATP) | Glycolysis/Gluconeogenesis | 1.814E-05 | 9.571E+01 | 0.000 |  | LOC_Os10g13700; LOC_Os04g50208 | 0/2 | Up-regulated |
| **PFK1[c]** | 6-phosphofructokinase | Glycolysis/Gluconeogenesis | -1.579E-01 | 4.668E+01 | 0.000 | LOC_Os09g24910 | LOC_Os04g39420; LOC_Os05g44922 | 1/2 | Up-regulated |
| **PPDK2[c]** | Pyruvate phosphate dikinase | Glycolysis/Gluconeogenesis | -2.992E-01 | 5.205E+01 | 0.000 |  | LOC_Os03g31750 | 0/1 | Up-regulated |
| **PFP[c]** | Diphosphate--fructose-6-phosphate 1-phosphotransferase | Glycolysis/Gluconeogenesis | -1.404E-01 | 1.871E+01 | 0.000 | LOC_Os02g48360; LOC_Os06g22060 | LOC_Os06g13810; LOC_Os08g25720 | 2/2 (D) | Metabolic regulation |
| **PGLYCM[c]** | Phosphoglycerate mutase | Glycolysis/Gluconeogenesis | -1.404E-01 | 1.507E-01 | 0.880 |  |  |  |  |
| **ENO1[c]** | Phosphopyruvate hydratase | Glycolysis/Gluconeogenesis | 7.889E+00 | 1.507E-01 | 0.880 |  |  |  |  |
| **PYK[p]** | Pyruvate kinase | Glycolysis/Gluconeogenesis | 2.793E-01 | 4.532E+03 | 0.000 | LOC_Os01g47080 | LOC_Os07g08340; LOC_Os10g42100; LOC_Os11g05110; LOC_Os12g05110 | 1/4 (U) | Metabolic regulation |
| **PPDK1[p]** | Pyruvate phosphate dikinase | Glycolysis/Gluconeogenesis | 2.925E-02 | 7.078E+02 | 0.000 |  |  |  |  |
| **PGI[p]** | Glucose-6-phosphate isomerase | Glycolysis/Gluconeogenesis | 4.294E-03 | 1.721E+02 | 0.000 |  |  |  |  |
| **TPI[p]** | Triosephosphate isomerase | Glycolysis/Gluconeogenesis | 2.862E-01 | 8.567E+02 | 0.000 | LOC_Os09g36450 |  | 1/0 | Down-regulated |
| **GAPDH[p]** | Glyceraldehyde 3-phosphate dehydrogenase (phosphorylating) | Glycolysis/Gluconeogenesis | 2.818E-01 | 8.682E+02 | 0.000 |  |  |  |  |
| **PGK[p]** | Phosphoglycerate kinase | Glycolysis/Gluconeogenesis | 2.818E-01 | 8.682E+02 | 0.000 |  |  |  |  |
| **PPS[p]** | Pyruvate,water dikinase | Glycolysis/Gluconeogenesis | -1.514E-05 | 9.084E+01 | 0.000 |  |  |  |  |
| **PFK1[p]** | 6-phosphofructokinase | Glycolysis/Gluconeogenesis | -2.103E-05 | 8.997E+01 | 0.000 | LOC_Os10g26570 |  | 1/0 (D) | Metabolic regulation |
| **ALD[p]** | Fructose-bisphosphate aldolase | Glycolysis/Gluconeogenesis | -2.103E-05 | 8.997E+01 | 0.000 |  | LOC_Os08g02700; LOC_Os11g07020 | 0/2 | Up-regulated |
| **FBP2[p]** | Fructose-bisphosphatase | Glycolysis/Gluconeogenesis | -1.285E-05 | 9.203E+01 | 0.000 |  |  |  |  |
| **PFK2[p]** | 6-phosphofructokinase | Glycolysis/Gluconeogenesis | -1.285E-05 | 9.203E+01 | 0.000 |  |  |  |  |
| **ENO1[p]** | Phosphopyruvate hydratase | Glycolysis/Gluconeogenesis | 6.688E+00 | 4.328E-01 | 0.665 |  |  |  |  |
| **PGLYCM[p]** | Phosphoglycerate mutase | Glycolysis/Gluconeogenesis | 6.688E+00 | 4.328E-01 | 0.665 |  |  |  |  |
| **ALDH1[p]** | aldehyde dehydrogenase | Fermentation | -3.569E-03 | 3.169E+02 | 0.000 | LOC_Os06g15990; LOC_Os11g08300 |  |  | Up-regulated |
| **ACS[p]** | acetate--CoA ligase | Fermentation | -1.147E-03 | 1.732E+02 | 0.000 |  | LOC_Os04g33190 | 0/1 | Up-regulated |
| **ADH[c]** | alcohol dehydrogenase | Fermentation | -1.595E+00 | 2.327E+02 | 0.000 |  | LOC_Os11g10510; LOC_Os11g10520 | 0/2 | Up-regulated |
| **PDC[c]** | pyruvate decarboxylase | Fermentation | -8.284E-01 | 1.168E+02 | 0.000 |  | LOC_Os01g06660; LOC_Os05g39310; LOC_Os03g18220; LOC_Os05g39320 | 0/4 | Up-regulated |
| **LDH[c]** | L-lactate dehydrogenase | Fermentation | -7.313E-01 | 1.184E+02 | 0.000 |  |  |  |  |
| **COX[m]** | Cytochrome c oxidase (complex IV) | Oxidative phosphorylation | 1.259E+00 | 3.596E+05 | 0.000 | LOC_Os01g42650; LOC_Os03g27290 |  | 2/0 | Down-regulated |
| **CCOR[m]** | Q-cytochrome c oxidoreductase (complex III) | Oxidative phosphorylation | 2.519E+00 | 3.596E+05 | 0.000 | LOC_Os02g33730; LOC_Os11g06340 | LOC_Os08g14860 | 2/1 | Down-regulated |
| **NAD9[m]** | NADH-coenzyme Q oxidoreductase (complex I) | Oxidative phosphorylation | 2.514E+00 | 3.444E+04 | 0.000 | LOC_Os01g61410; LOC_Os07g37730; LOC_Os05g45730; LOC_Os10g42540; LOC_Os03g50540 |  | 5/0 | Down-regulated |
| **ATPS[m]** | ATP synthase (complex V) | Oxidative phosphorylation | 3.785E+00 | 3.329E+03 | 0.000 | LOC_Os07g31300; LOC_Os08g37320; LOC_Os10g17280 |  | 3/0 | Down-regulated |
| **TKT2[p]** | Transketolase | Pentose Phosphate Pathway | 5.099E-03 | 1.355E+03 | 0.000 | LOC_Os04g19740; LOC_Os06g04270 |  | 2/0 | Down-regulated |
| **RPI[p]** | Ribose 5-phosphate epimerase | Pentose Phosphate Pathway | 4.311E-03 | 1.365E+03 | 0.000 | LOC_Os07g08030 |  | 1/0 | Down-regulated |
| **RPE[p]** | ribulose phosphate 3-epimerase | Pentose Phosphate Pathway | 4.315E-03 | 5.305E+02 | 0.000 | LOC_Os03g07300 |  | 1/0 | Down-regulated |
| **TKT1[p]** | Transketolase | Pentose Phosphate Pathway | 7.843E-04 | 3.189E+02 | 0.000 | LOC_Os04g19740; LOC_Os06g04270 |  | 2/0 | Down-regulated |
| **TALA[p]** | Transaldolase | Pentose Phosphate Pathway | 7.843E-04 | 3.189E+02 | 0.000 | LOC_Os01g70170 |  | 1/0 | Down-regulated |
| **PDHE1[p]** | Pyruvate dehydrogenase (lipoamide) | Pyruvate dehydrogenase complex | 2.036E-01 | 9.284E+03 | 0.000 | LOC_Os12g42230 | LOC_Os03g44300 | 1/1 |  |
| **PDHE3[p]** | dihydrolipoyl dehydrogenase | Pyruvate dehydrogenase complex | 2.036E-01 | 9.284E+03 | 0.000 |  | LOC_Os12g08170 | 0/1 |  |
| **PDHE2[p]** | Dihydrolipoamide S-acetyltransferase | Pyruvate dehydrogenase complex | 2.036E-01 | 9.284E+03 | 0.000 |  | LOC_Os01g23610 | 0/1 |  |
| **PDHE1[m]** | Pyruvate dehydrogenase (lipoamide) | Pyruvate dehydrogenase complex | 5.116E-01 | 1.503E+04 | 0.000 | LOC_Os08g42410; LOC_Os06g13720; LOC_Os02g50620 |  | 3/0 | Down-regulated |
| **PDHE2[m]** | Dihydrolipoamide S-acetyltransferase | Pyruvate dehydrogenase complex | 5.116E-01 | 1.503E+04 | 0.000 | LOC_Os06g30460; LOC_Os06g01630 |  | 2/0 | Down-regulated |
| **PDHE3[m]** | dihydrolipoyl dehydrogenase | Pyruvate dehydrogenase complex | 5.116E-01 | 1.503E+04 | 0.000 |  |  | 0/0 |  |
| **FK[c]** | Fructokinase | Sucrose metabolism | -3.788E-02 | 1.855E+04 | 0.000 | LOC_Os08g02120 | LOC_Os01g66940 | 1/1 (U) | Up-regulated |
| **SUS[c]** | Sucrose synthase | Sucrose metabolism | -3.853E-02 | 1.373E+04 | 0.000 |  | LOC_Os04g17650; LOC_Os04g24430; LOC_Os07g42490; LOC_Os03g28330; LOC_Os06g09450 | 5/0 | Up-regulated |
| **UGP[c]** | UTP--glucose-1-phosphate uridylyltransferase | Sucrose metabolism | -1.321E-01 | 2.322E+04 | 0.000 | LOC_Os02g02560 |  | 1/0 | Metabolic regulation |
| **IPP[p]** | Inorganic pyrophosphatase | Sucrose metabolism | 1.594E-01 | 3.557E+03 | 0.000 |  |  |  |  |
| **EPI[c]** | Glucose-6 phosphate 1-epimerase | Sucrose metabolism | -2.604E-01 | 1.506E+04 | 0.000 | LOC_Os04g56290 | LOC_Os01g46950; LOC_Os08g14330 | 1/2 | Up-regulated |
| **PGI[c]** | Glucose-6-phosphate isomerase | Sucrose metabolism | -2.604E-01 | 6.272E+03 | 0.000 |  | LOC_Os03g56460; LOC_Os06g14510 | 0/2 | Up-regulated |
| **CIN[c]** | Beta-fructofuranosidase | Sucrose metabolism | 1.807E-03 | 1.706E+02 | 0.000 | LOC_Os02g01590; LOC_Os04g45290; LOC_Os02g33110; LOC_Os11g07440; LOC_Os01g22900) |  | 5/0 | Down-regulated |
| **MDH[m]** | Malate dehydrogenase | TCA Cycle | 1.521E+00 | 1.720E+04 | 0.000 | LOC_Os05g49880 |  | 1/0 | Down-regulated |
| **CSY[m]** | Citrate synthase | TCA Cycle | 5.116E-01 | 1.591E+04 | 0.000 | LOC_Os02g10070 |  | 1/0 | Down-regulated |
| **ACO1[m]** | isocitrate hydrolyase | TCA Cycle | 5.115E-01 | 1.199E+04 | 0.000 |  |  |  |  |
| **ACO2[m]** | citrate hydrolyase | TCA Cycle | 5.115E-01 | 1.199E+04 | 0.000 |  |  |  |  |
| **IDP[m]** | Isocitrate dehydrogenase (NADP+) | TCA Cycle | 5.115E-01 | 1.069E+04 | 0.000 | LOC_Os01g14580 | LOC_Os02g38200 | 1/1 |  |
| **FUM[m]** | Fumarate hydratase | TCA Cycle | 1.030E-02 | 2.479E+02 | 0.000 | LOC_Os03g21950 |  | 1/0 | Down-regulated |
| **SDH[m]** | Succinate-Q oxidoreductase (complex II) | TCA Cycle | 4.392E-03 | 1.882E+02 | 0.000 | LOC_Os07g04240 |  | 1/0 | Down-regulated |
| **AKGDH[m]** | 2-oxoglutarate dehydrogenase | TCA Cycle | 1.655E-01 | 1.374E+02 | 0.000 | LOC_Os04g32020 |  | 1/0 | Down-regulated |
| **SUCLG[m]** | Succinate--CoA ligase (ADP-forming) | TCA Cycle | 1.655E-01 | 1.374E+02 | 0.000 |  |  |  |  |
| **IDH[m]** | Isocitrate dehydrogenase (NAD+) | TCA Cycle | 2.007E-05 | 1.000E+02 | 0.000 |  | LOC_Os02g38200 | 0/1 |  |
