# Supplementary material for: Metabolic and transcriptional regulatory mechanisms underlying the anoxic adaptation of rice coleoptile
Source: AoB Plants. 2014 Jun 3;6:plu026. doi: 10.1093/aobpla/plu026 (PMC4077593; doi:10.1093/aobpla/plu026)
Supplement: Additional Information [file supp_plu026_plu026supp_data4.doc]

**Supplemental File S4**

# Combined in silico metabolic flux sampling and microarray data analysis reveals key transcriptional mechanisms in anoxic adaptation of rice coleoptile

Meiyappan Lakshmanan, Bijayalaxmi Mohanty, Sun-Hyung Lim, Sun-Hwa H3 and Dong-Yup Lee

Department of Chemical and Biomolecular Engineering, National University of Singapore, Singapore.

**List of genes in negative sets**

**Negative set1 (Randomly selected non-anoxia specific genes from rice coleoptile microarray data)**

**LOC_Os04g51780**

**LOC_Os11g03760**

**LOC_Os05g50660**

**LOC_Os02g34510**

**LOC_Os01g53840**

**LOC_Os03g61140**

**LOC_Os09g34000**

**LOC_Os01g33000**

**LOC_Os01g42250**

**LOC_Os06g11370**

**LOC_Os12g10650**

**LOC_Os03g24860**

**LOC_Os02g22090**

**LOC_Os02g53490**

**LOC_Os03g14800**

**LOC_Os02g35860**

**LOC_Os01g53670**

**LOC_Os06g33340**

**LOC_Os03g05280**

**LOC_Os03g45340**

**LOC_Os12g06890**

**LOC_Os09g24440**

**LOC_Os04g49660**

**LOC_Os01g08410**

**LOC_Os11g07916**

**LOC_Os01g56140**

**Negative set2 (Drought responsive genes)**

**LOC_Os03g21620**

**LOC_Os08g20020**

**LOC_Os04g58310**

**LOC_Os06g10530**

**LOC_Os02g53410**

**LOC_Os02g08320**

**LOC_Os10g09850**

**LOC_Os04g19740**

**LOC_Os04g32080**

**LOC_Os01g16920**

**LOC_Os12g06010**

**LOC_Os03g47120**

**LOC_Os03g02050**

**LOC_Os05g32710**

**LOC_Os10g35050**

**LOC_Os01g59100**

**LOC_Os12g05330**

**LOC_Os01g53650**

**LOC_Os01g45460**

**LOC_Os07g07240**

**LOC_Os01g73110**

**LOC_Os01g44250**

**LOC_Os06g02040**

**LOC_Os01g73110**

**LOC_Os01g44250**

**LOC_Os06g02040**
