# Supplementary material for: Metabolic and transcriptional regulatory mechanisms underlying the anoxic adaptation of rice coleoptile
Source: AoB Plants. 2014 Jun 3;6:plu026. doi: 10.1093/aobpla/plu026 (PMC4077593; doi:10.1093/aobpla/plu026)
Supplement: Additional Information [file supp_plu026_plu026supp_data5.doc]

**Supplemental File S5**

# Combined in silico metabolic flux sampling and microarray data analysis reveals key transcriptional mechanisms in anoxic adaptation of rice coleoptile

Meiyappan Lakshmanan, Bijayalaxmi Mohanty, Sun-Hyung Lim, Sun-Hwa H3 and Dong-Yup Lee

Department of Chemical and Biomolecular Engineering, National University of Singapore, Singapore.

**Potential cis-elements identified in the promoter sequences of randomly selected genes not affected by anoxia during germination of rice.**

| **Cis-elements** | **Motifs** | **Associated TFs** | **% (TIC), e-value** |
| --- | --- | --- | --- |
| AT-hook/PE1-like | AAAAAGTGA  AAAAATAT | MYB (PF1)  MYB (PF1) | 62 (13.56), 2e-004  58 (14.30), 6e-005 |
| GT-element-like | GTAGTTTAC  TGGTTCGT | MYB (GT-1)  MYB (GT-1) | 69 (12.16), 4e-004  50 (11.78), 2e-004 |
| GARE-like | AATAACAAAA  CTTTTGTA | Myb (R1, R2R3)  Myb (R1, R2R3) | 62 (12.92), 8e-005  58 (15.28), 5e-005 |
| MYB-box-like | TTTGGTTTT  AATAACAAAA  ATGTGGAT | MYB (R2R3)  MYB (R2R3)  MYB (R2R3, MCB1/2) | 54 (16.05), 1e-004  58 (15.28), 5e-005  54 (12.51), 3e-004 |
| AS-1/ocs-like | AAATTTGA  AGAAATAAAG | bZIP (Gr. D, I, S)  bZIP | 62 (12.85), 3e-004  54 (14.28), 9e-004 |
| GCN4 motif | AATAACAAAA | bZIP (RISBZ1) | 58 (15.28), 5e-005 |
| AAAAG/CTTTT-element-like | CTTTTGTA  AAAAAGTGA  AGAAATAAAG | DOF (DOF1/4/11)  DOF (DOF1/4/11) | 62 (12.92), 6e-005  62 (13.56), 2e-004  54 (14.28), 9e-004 |
| Alfin1 binding site | AACTCCAC | Alfin 1 (PHD-zinc finger) | 58 (13.82), 1e-004 |
| DBP-binding site-like | AAAAATAT | DBP | 58 (14.30), 6e-005 |

**Potential cis-elements identified in the promoters of up-regulated genes associated with drought response in rice.**

| **Cis-elements** | **Motifs** | **Associated TFs** | **% (TIC), e-value** |
| --- | --- | --- | --- |
| AT-hook/PE1-like | ATTTTTAGA | MYB (PF1) | 62 (13.71), 4e-004 |
| GT-element-like | GTTGGTCG  TTGTGCTC | MYB (GT-1)  MYB (GT-1/GT-3b) | 65 (11.53), 3e-004  50 (12.83), 3e-005 |
| MYB-box-like | ACACCAAA  CAAAACCA | MYB (R2R3) (MYB 15)  MYB (R2R3) (MYB 2, MYB 80) | 65 (11.34), 2e-004  58 (11.75), 4e-004 |
| AS-1/ocs-like | ACGTGACA | bZIP (Gr. D, I, S) | 58 (12.33), 8e-005 |
| ABRE-like/G-box-like | GCGGGCCAA  AACATGGC  GCGAAGCT  CTCACCGT  AGCCGCTTC  CCTGCCAT  AGGACGAG  CTCCACCCG  ACTCGCAC ACGTGACA  GGGACGCGT | bZIP (Gr. A)  bZIP (Gr. A)  bZIP (Gr. A)  bZIP (Gr. A)  bZIP (Gr. A)  bZIP (Gr. A)  bZIP (Gr. A)  bZIP (Gr. A)(DPBF1, DPBF2)  bZIP (TRAB1)  bZIP (TRAB1, OSBZ8)  bZIP (TRAB1) | 84 (11.36), 3e-003  81 (11.70), 9e-005  69 (11.27), 0e+000  65 (12.82), 2e-004  62 (13.39), 2e-004  62 (12.89), 8e-004  58 (12.58), 2e-004  58 (13.74), 4e-004  62 (12.64), 2e-004  58 (12.33), 8e-005  54 (14.29), 1e-004 |
| ABRE-like | ACACCAAA | ABI3 | 65 (11.34), 2e-004 |
| ABRE-like | CTCACCGT  CTCCACCCG | ABI4  ABI4 | 65 (12.82), 2e-004  58 (13.74), 4e-004 |
| GCC-box-like | GGCGGCGGC | ERF (I, IV, VII, X) | 66 (17.16), 2e-005 |
